# Functional Microbiomics Reveals Alterations of the Gut Microbiome and Host Co‐Metabolism in Patients With Alcoholic Hepatitis

**DOI:** 10.1002/hep4.1537

**Published:** 2020-06-19

**Authors:** Bei Gao, Yi Duan, Sonja Lang, Dinesh Barupal, Tsung‐Chin Wu, Luis Valdiviez, Bryan Roberts, Ying Yng Choy, Tong Shen, Gregory Byram, Ying Zhang, Sili Fan, Benjamin Wancewicz, Yan Shao, Kevin Vervier, Yanhan Wang, Rongrong Zhou, Lu Jiang, Shilpa Nath, Rohit Loomba, Juan G. Abraldes, Ramon Bataller, Xin M. Tu, Peter Stärkel, Trevor D. Lawley, Oliver Fiehn, Bernd Schnabl

**Affiliations:** ^1^ Department of Medicine University of California San Diego La Jolla CA; ^2^ Department of Medicine VA San Diego Healthcare System San Diego CA; ^3^ West Coast Metabolomics Center University of California Davis Davis CA; ^4^ Division of Mathematics University of California San Diego San Diego CA; ^5^ Department of Cell and Regenerative Biology University of Wisconsin‐Madison Madison WI; ^6^ Host‐Microbiota Interactions Laboratory Wellcome Sanger Institute Wellcome Genome Campus Hinxton United Kingdom; ^7^ Department of Medicine University of Alberta Edmonton AB Canada; ^8^ Division of Gastroenterology, Hepatology and Nutrition Department of Medicine Pittsburgh Liver Research Center University of Pittsburgh Medical Center Pittsburgh PA; ^9^ Department of Biostatistics and Bioinformatics Department of Family Medicine and Public Health University of California San Diego San Diego CA; ^10^ St. Luc University Hospital Université Catholique de Louvain Brussels Belgium

## Abstract

Alcohol‐related liver disease is a major public health burden, and the gut microbiota is an important contributor to disease pathogenesis. The aim of the present study is to characterize functional alterations of the gut microbiota and test their performance for short‐term mortality prediction in patients with alcoholic hepatitis. We integrated shotgun metagenomics with untargeted metabolomics to investigate functional alterations of the gut microbiota and host co‐metabolism in a multicenter cohort of patients with alcoholic hepatitis. Profound changes were found in the gut microbial composition, functional metagenome, serum, and fecal metabolomes in patients with alcoholic hepatitis compared with nonalcoholic controls. We demonstrate that in comparison with single omics alone, the performance to predict 30‐day mortality was improved when combining microbial pathways with respective serum metabolites in patients with alcoholic hepatitis. The area under the receiver operating curve was higher than 0.85 for the tryptophan, isoleucine, and methionine pathways as predictors for 30‐day mortality, but achieved 0.989 for using the urea cycle pathway in combination with serum urea, with a bias‐corrected prediction error of 0.083 when using leave‐one‐out cross validation. *Conclusion:* Our study reveals changes in key microbial metabolic pathways associated with disease severity that predict short‐term mortality in our cohort of patients with alcoholic hepatitis.

## Graphical abstract

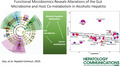

**Abbreviations**
AUC: area under the curve
AUROC: area under the receiver operating characteristic curve
AH_c: patients with alcoholic hepatitis with cirrhosis
AUD_nc: patients with alcohol use disorder without cirrhosis
AUD_c: patients with alcohol use disorder with cirrhosis
Ctrl: control
FIB‐4: Fibrosis‐4 index
G1: controls
G2: patients with alcohol use disorder without cirrhosis
G3: patients with alcohol use disorder with cirrhosis
G4: patients with alcoholic hepatitis without cirrhosis
G5: patients with alcoholic hepatitis with cirrhosis
INR: international normalized ratio
LDA: linear discriminant analysis
LEfSe: LDA effect size
MaAsLin2: multivariate association with linear models
MELD: Model for End‐Stage Liver Disease
N.S.: not significant
PWY: pathway

Alcohol‐related liver disease is a major health care burden and a leading cause of morbidity and mortality worldwide.^(^ ^1^ ^)^ The individual susceptibility of patients with alcohol use disorder to liver disease is highly variable. Some patients develop alcoholic hepatitis, a severe manifestation of alcohol‐related liver disease with a short‐term mortality of about 40%‐50%^(^ ^2^ ^)^ Pharmacologic treatment options for patients with alcoholic hepatitis include corticosteroids and pentoxifylline; however, they only provide a minimal survival benefit.^(^ ^3^ ^)^ The risk of progression from liver steatosis to more advanced disease is affected by many factors, including the duration and extent of alcohol misuse, gender, and genetic factors.^(^ ^1^ ^)^ Recent studies showed that gut microbiota, a potentially modifiable factor, plays an important role in alcohol‐related liver disease.^(^ ^4^, ^5^, ^6^ ^)^

Colonization of mice with fecal material from patients with alcoholic hepatitis increased susceptibility to ethanol‐induced liver disease.^(^ ^7^ ^)^ A small pilot study showed that fecal microbiota transplantation from heathy subjects to steroid‐ineligible patients with severe alcoholic hepatitis improved patient survival.^(^ ^8^ ^)^ Therefore, microbiota‐centered therapeutic approaches might be developed to reduce alcohol‐related liver disease.^(^ ^9^ ^)^ Several single‐center studies have analyzed changes in the microbial composition in patients with alcohol‐related liver diseases and alcoholic hepatitis.^(^ ^10^, ^11^, ^12^, ^13^ ^)^ In addition, metabolomic studies have been performed in patients with alcoholic hepatitis.^(^ ^14^, ^15^ ^)^ Recently, an integrated analysis of bile acid homeostasis and gut microbiota composition was performed in patients with alcoholic hepatitis.^(^ ^16^ ^)^ However, other host‐microbiota co‐metabolism and their impact on alcohol‐related liver disease are not well characterized and understood.

In the present study, we investigated alterations in the functional capacities of the gut microbiota by shotgun metagenomic sequencing, and gut microbiome and host co‐metabolism by untargeted metabolomic profiling. We first identified key metabolites through a discovery patient cohort and further validated these metabolites through a validation patient cohort. The goal of the present study is to characterize the metabolic reprogramming of the gut microbiota and host co‐metabolism in a cohort of patients with alcohol‐related liver disease and predict short‐term mortality in patients with alcoholic hepatitis.

## Materials and Methods

### Patient Cohorts

Patient cohorts have been described.^(^ ^17^, ^18^, ^19^, ^20^ ^)^ For this untargeted metabolomics study, serum and fecal samples were collected from a discovery cohort that included 17 nonalcoholic controls, 32 patients with alcohol use disorder, and 13 patients with alcoholic hepatitis for the comparison among three groups to identify key metabolites. These 13 patients with alcoholic hepatitis were selected because they did not receive antibiotics, steroids, or pentoxifylline at the time of specimen collection. As a validation cohort, 141 more serum samples were used from patients with alcoholic hepatitis for correlation analysis and mortality‐prediction model development. For shotgun metagenomic analysis, fecal DNA was extracted from 9 nonalcoholic controls, 41 patients with alcohol use disorder, and 81 patients with alcoholic hepatitis. Nonalcoholic controls are social drinkers who consumed less than 20 g of alcohol per day. Patients were diagnosed as having alcohol use disorder if they fulfilled the Diagnostic and Statistical Manual of Mental Disorders, Fourth Edition, criteria.^(^ ^21^ ^)^ Different stages of liver disease are present in the patients with alcohol use disorder, ranging from steatosis to steatohepatitis, with or without significant fibrosis. Nonalcoholic controls or patients with alcohol use disorder did not take antibiotics or immunosuppressive medication during the 2 months preceding enrollment. Inclusion and exclusion criteria of patients with alcoholic hepatitis are included in the Supporting Information, as reported in our previous publication.^(^ ^17^ ^)^ Patients with alcoholic hepatitis were enrolled in 10 different medical centers in Europe and North America. The clinical picture was consistent with alcoholic hepatitis in all patients. Liver biopsies were done only if indicated as part of routine clinical care for the purpose of alcoholic hepatitis diagnosis. For patients who underwent liver biopsy, the liver histology was in line with the diagnosis of alcoholic hepatitis. The protocol was approved by the Ethics Committee of each participating center. Written informed consent was obtained from each subject. Metabolomic and metagenomic data acquisition and data analysis are included in the Supporting Information.

### Statistical Analysis

Statistical analysis was performed using R statistics software (version 3.5.1). Metabolomics data were normalized using the sum of all identified metabolites to scale each sample. Kruskal‐Wallis test was used to calculate the *P* values in multiple groups, and Mann‐Whitney‐Wilcoxon test was used for the comparison between two groups. Adjusted *P* values were calculated using the Benjamini–Hochberg procedure to control the false discovery rate. Principal component analysis plots and heatmap were generated using MetaboAnalyst 4.0.^(^ ^22^ ^)^ A random forest model was built to predict the 30‐day mortality in patients with alcoholic hepatitis using serum metabolites. Synthetic minority oversampling technique was used to oversample the minor class to obtain balanced data. Extra‐trees classifier was used to select 10 variables from all annotated serum metabolites based on the feature importance. Random forest model was built using the H2O platform (https://www.h2o.ai). The data set was split into training and test data sets (80:20 stratified splits). Stratified 5‐fold cross‐validation was performed on the training set to choose the tuning parameters for the random forest model.

Linear discriminant analysis (LDA) effect size (LEfSe) was performed on the metagenomic data.^(^ ^23^ ^)^ Multivariate association with linear models (MaAsLin2) was used to check the association among the amount of alcohol intake, gender, and metagenomics and metabolomics data. Spearman correlation was conducted to correlate serum metabolites with clinical parameters. Univariate Cox regression model was used to detect associations of serum metabolites with 30‐day mortality. Additionally, a multivariate Cox regression model was performed to adjust for different variables. Patients who were lost to follow‐up were censored at the day they were last seen alive. Maximally selected rank statistic was used to determine the optimal cutoff value that represents the maximum difference of two alcoholic hepatitis groups regarding 30‐day survival.^(^ ^24^ ^)^ Kaplan‐Meier curves, along with log‐rank test, were used to compare 30‐day survival between two groups. To test the diagnostic value of multi‐omics and to improve the performance of the single omics, we modeled the binary 30‐day mortality outcome using a logistic regression with microbial pathways and serum metabolites as the predictors. To be included in the model, serum metabolites need to be either a direct substrate or product for certain microbial pathways. Only models with an area under the receiver operating characteristic curve (AUROC) score larger than 0.85 are discussed in our study. Furthermore, we performed leave‐one‐out cross validation to validate our prediction model in this patient cohort, which uses a single observation from the original sample as the validation data and the remaining observations as the training data.^(^ ^25^ ^)^

## Results

### Patient Cohorts

Untargeted metabolomic analysis was first performed on serum and fecal samples collected from a discovery cohort consisting of 17 nonalcoholic controls, 18 patients with alcohol use disorder without cirrhosis, 3 patients with alcohol use disorder with cirrhosis (Fibrosis‐4 index [FIB‐4] > 3.25), and 6 patients with alcoholic hepatitis with cirrhosis (fibrosis stage F3‐F4). The stage of cirrhosis was assessed using noninvasive assessment of fibrosis, FIB‐4 score in patients with alcohol use disorder, and using liver biopsy in patients with alcoholic hepatitis. The first set of 6 patients with alcoholic hepatitis was biopsied among the 13 patients who did not receive antibiotics, steroids, or pentoxifylline (Supporting Table S1 and Supporting Fig. S1). The median age and body mass index were higher in patients with alcoholic hepatitis, compared with controls or patients with alcohol use disorder. Increased body mass index is likely due to the presence of ascites in 62% of patients with alcoholic hepatitis. Untargeted metabolomic analysis was further performed on serum samples collected from a second set of 141 patients with alcoholic hepatitis (validation cohort) for the purpose of correlation analysis and prediction model development. There was no significant difference in the characteristics between the two sets of patients with alcoholic hepatitis, although the median age for the 141 patients was lower than that of the 13 patients with alcoholic hepatitis (Supporting Tables S1 and S2). The median of the Model for End‐Stage Liver Disease (MELD) score in the first and second set of patients with alcoholic hepatitis was 22 and 24, respectively. In the second set of patients with alcoholic hepatitis, 43% of patients received treatment with steroids, 6% with pentoxifylline, and 27% were treated with antibiotics. Liver biopsy was performed in 54% of the patients in both sets. Based on the liver biopsy, these patients with alcoholic hepatitis were stratified into a noncirrhosis group (F0‐F2) and a cirrhosis group (F3‐F4) for the comparison of metabolomics data.

For shotgun metagenomic analysis, limited by the availability of stool specimens, 9 nonalcoholic controls, 21 patients with alcohol use disorder without cirrhosis (FIB‐4 ≤ 3.25), 4 patients with alcohol use disorder with cirrhosis (FIB‐4 > 3.25), 7 patients with alcoholic hepatitis without cirrhosis (F0‐F2), and 38 patients with cirrhosis (F3‐F4) were evaluated (Supporting Tables S3 and S4). The stage of cirrhosis in patients with alcoholic use disorder was assessed using a noninvasive assessment of fibrosis, the FIB‐4 score. The stage of cirrhosis in patients with alcoholic hepatitis was assessed based on liver biopsy. Liver biopsy was available from 58% of patients with alcoholic hepatitis. At the time of specimen collection, 39% of patients with alcoholic hepatitis received steroids, 9% received pentoxifylline, and 23% of patients were treated with antibiotics. Metabolomic and metagenomic data did not differ significantly among the regions/centers, where patients with alcoholic hepatitis were enrolled (Supporting Fig. S2). In addition, treatment of patients with alcoholic hepatitis with antibiotics (Supporting Fig. S3) or steroids (Supporting Fig. S4) did not significantly affect the metabolomic or metagenomic data.

### Gut Microbiota Composition Is Changed in Patients With Alcoholic Hepatitis

**Fig. 1:**
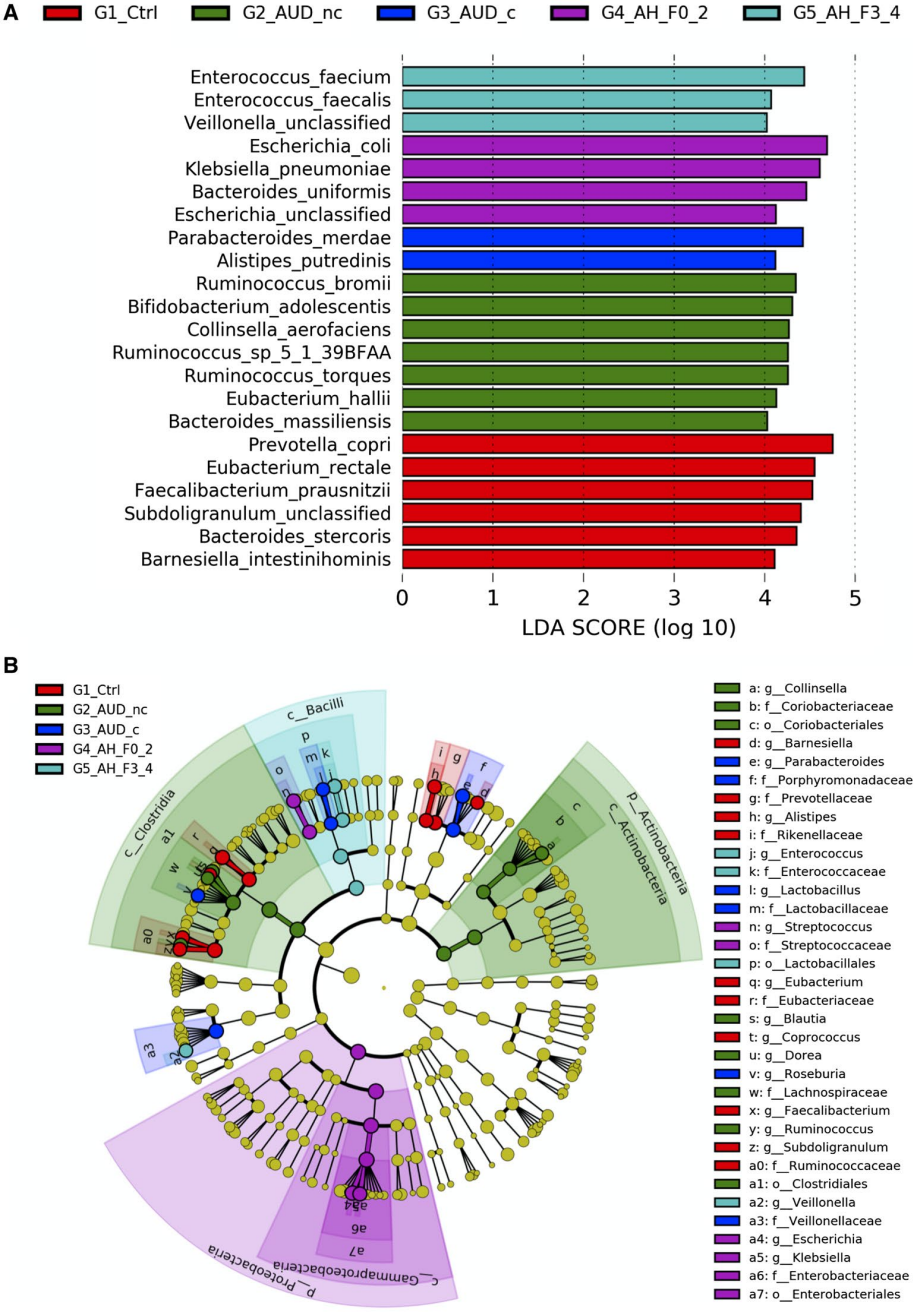
LEfSe analysis of the gut microbial taxonomy. (A) Enriched species (LDA score > 4) in controls, patients with alcohol use disorder without cirrhosis, patients with alcohol use disorder with cirrhosis, patients with alcoholic hepatitis without cirrhosis, and patients with alcoholic hepatitis with cirrhosis. (B) Taxonomic representation of statistically and biologically consistent differences in the five groups. Differences are represented in the color of the most abundant class. Each circle's diameter is in proportion to that taxon's abundance. Abbreviations: G1_Ctrl, controls; G2_AUD_nc, patients with alcohol use disorder without cirrhosis; G3_AUD_c, patients with alcohol use disorder with cirrhosis; G4_AH_F0_2, patients with alcoholic hepatitis without cirrhosis; G5_AH_F3_4, patients with alcoholic hepatitis with cirrhosis.

To determine microbial organisms that are most likely to explain the differences among patients with alcoholic hepatitis, alcohol use disorder, and control individuals, LEfSe was performed based on metagenomic sequencing, along with additional statistical tests for biological consistency and effect relevance.^(^ ^23^ ^)^ LEfSe analysis revealed 6, 7, 2, 4, and 3 bacteria species with LDA score larger than 4.0 in controls, patients with alcohol use disorder without cirrhosis, patients with alcohol use disorder with cirrhosis, patients with alcoholic hepatitis without cirrhosis, and patients with alcoholic hepatitis with cirrhosis, respectively (Fig. 1A). Taxonomic representation of statistically and biologically consistent differences in the five groups was shown in Fig. 1B.

### Alterations in the Functional Metagenome in patients With Alcoholic Hepatitis

**Fig. 2:**
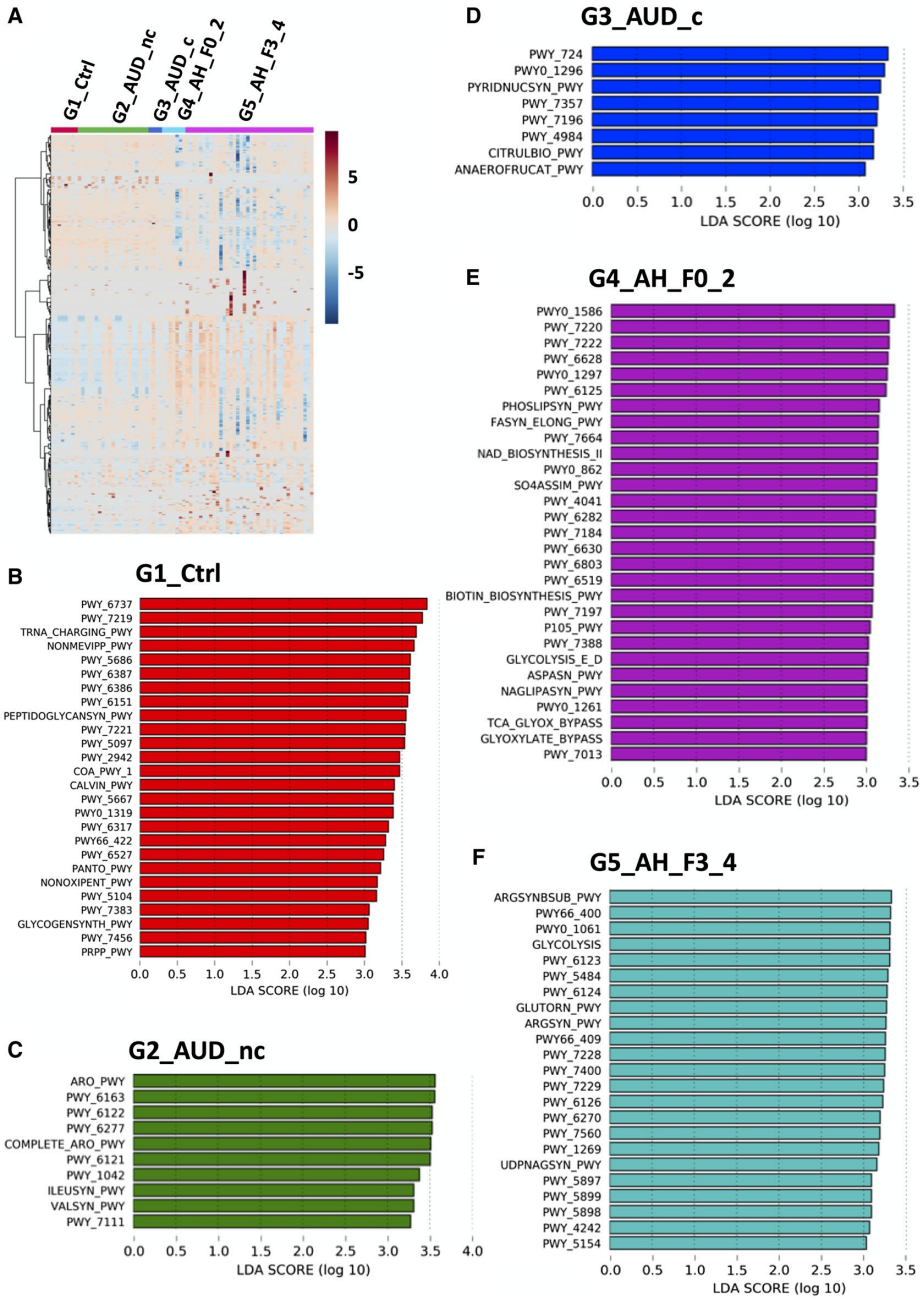
Pathway analysis of the gut metagenome. (A) Hierarchical clustering of microbial pathways in controls, patients with alcohol use disorder without cirrhosis, patients with alcohol use disorder with cirrhosis, patients with alcoholic hepatitis without cirrhosis, and patients with alcoholic hepatitis with cirrhosis. Enriched microbial pathways (LDA score > 3) in controls (B), patients with alcohol use disorder without cirrhosis (C), patients with alcohol use disorder with cirrhosis (D), patients with alcoholic hepatitis without cirrhosis (E), and patients with alcoholic hepatitis patients with cirrhosis (F). Abbreviation: PWY, pathway.

A total of 474 MetaCyc pathways (Supporting Table S5) were detected in the metagenomics analysis. Hierarchical clustering of these pathways showed that the functional metagenome profile of patients with alcoholic hepatitis was different from controls or patients with alcohol use disorder (Fig. 2A). LEfSe analysis showed that 26, 10, 8, 29, and 23 pathways with LDA score larger than 3.0 were found in control subjects, patients with alcohol use disorder without cirrhosis, patients with alcohol use disorder with cirrhosis, patients with alcoholic hepatitis without cirrhosis, and patients with alcoholic hepatitis with cirrhosis, respectively (Fig. 2B‐F). The name and LDA score of these pathways are listed in Supporting Table S6.

In addition to the LEfSe analysis based on the disease status, we performed MaAsLin2 analysis to identify the microbes and microbial pathways that were associated with the alcohol intake. We found nine bacteria and six microbial pathways that were associated with alcohol intake (*P* < 0.05). The results are summarized in Supporting Table S7.

### Serum and Fecal Metabolome are Significantly Different in Alcoholic Hepatitis Patients

**Fig. 3:**
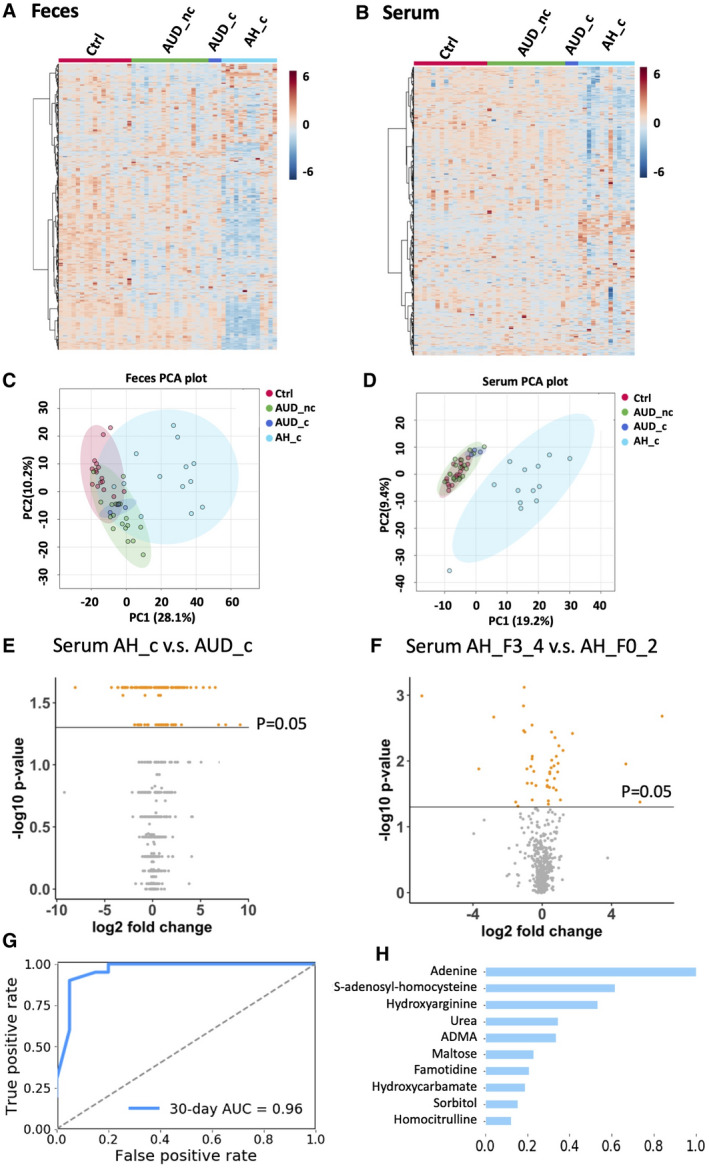
Untargeted metabolomics profiling of fecal and serum samples. Hierarchical clustering of fecal (A) and serum metabolites (B). Principal component analysis of fecal metabolites (C) and serum metabolites (D). (E) Significantly altered serum metabolites in patients with alcoholic hepatitis with cirrhosis (AH_c) compared to patients with alcohol use disorder with cirrhosis (AUD_c). Fold change = AH_c/AUD_c. (F) Significantly altered serum metabolites in patients with alcoholic hepatitis with cirrhosis (AH_F3_4) compared to patients with alcoholic hepatitis without cirrhosis (AH_F0_2). Fold change = AH_F3_4/AH_F0_2. (G) Random forest model for the 30‐day mortality prediction using serum metabolomics data. Alive group, n = 99; deceased group, n = 19. (H) Variable importance. Abbreviations: AH_c, patients with alcoholic hepatitis with cirrhosis; AUD_nc, patients with alcohol use disorder without cirrhosis; AUD_c, patients with alcohol use disorder with cirrhosis; Ctrl, control; AH_F0_2, patients with alcoholic hepatitis without cirrhosis; AH_F3_4, patients with alcoholic hepatitis with cirrhosis. PCA, principal component analysis.

A total of 546 metabolites were annotated in the discovery cohort (Supporting Table S8). Multivariate analysis of the annotated metabolites showed that fecal and serum metabolome in patients with alcoholic hepatitis was separated from control and alcohol use disorder groups, as shown in heatmap and principal component analysis plots (Fig. 3A‐D). Compared to patients with alcohol disorder with cirrhosis, a total of 128 serum metabolites were significantly different (*P* < 0.05) in patients with alcoholic hepatitis with cirrhosis, with 75 increased and 51 decreased (Fig. 3E and Supporting Table S9).

We further performed untargeted metabolomics on serum samples collected from the validation cohort to study the association with 30‐day mortality in patients with alcoholic hepatitis. Compared to patients with alcoholic hepatitis without cirrhosis, a total of 45 metabolites were significantly different in patients with alcoholic hepatitis with cirrhosis (*P* < 0.05), with 26 increased and 19 decreased (Fig. 3F and Supporting Table S10). We built a random forest model to predict the 30‐day mortality in patients with alcoholic hepatitis using serum metabolomics data. The AUROC was 0.96 (Fig. 3G). The variable importance is shown in Fig. 3H. Furthermore, to evaluate the performance of the multi‐omics, we modeled the binary 30‐day mortality outcome using a logistic regression with microbial pathways and serum metabolites as the predictors. Models with AUROC score larger than 0.85 are discussed in the following functional metagenomic part of this study, including tryptophan, isoleucine, methionine, and urea cycle. Spearman correlation of these metabolites with clinical parameters and outcomes are shown in Supporting Fig. S5.

### Microbial‐Dependent Tryptophan Metabolism Is Dysregulated in Alcoholic Hepatitis

**Fig. 4:**
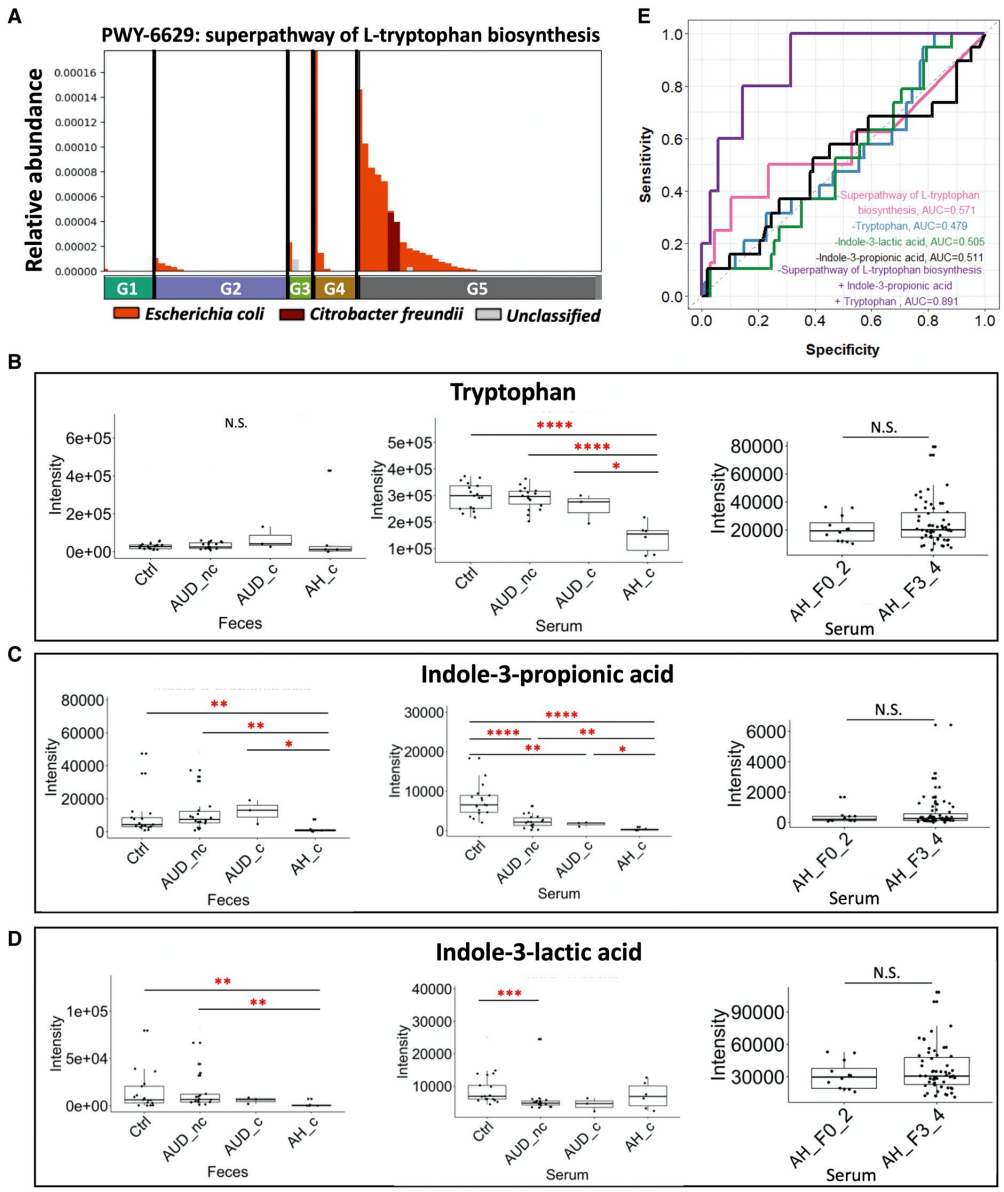
Tryptophan metabolism. (A) Relative abundance of microbial superpathway of L‐tryptophan biosynthesis. G5 versus G2: *P* = 0.039. Fecal and serum level of tryptophan (B), indole‐3‐propionic acid (C), and indole‐3‐lactic acid (D). (E) Area under the curve using different predictors related to tryptophan metabolism. Alive group, n = 38; deceased group, n = 5. *P* > 0.05, *P < 0.05, **P < 0.01, ***P < 0.001, ****P < 0.0001. Abbreviations: AH_F0_2, patients with alcoholic hepatitis without cirrhosis; AH_F3_4, patients with alcoholic hepatitis with cirrhosis; AH_c, patients with alcoholic hepatitis with cirrhosis; AUD_nc, patients with alcohol use disorder without cirrhosis; AUD_c, patients with alcohol use disorder with cirrhosis; Ctrl, control; AUC, area under the curve; G1, controls; G2, patients with alcohol use disorder without cirrhosis; G3, patients with alcohol use disorder with cirrhosis; G4, patients with alcoholic hepatitis without cirrhosis; G5, patients with alcoholic hepatitis with cirrhosis; N.S., not significant.

As an essential aromatic amino acid, tryptophan is the precursor of various other metabolites, such as indole derivatives. Tryptophan metabolism and production of indole derivatives is under the direct control of the gut microbiota.^(^ ^26^ ^)^ As an alternative source to dietary intake, gut microbes have the ability to synthesize tryptophan. In patients with alcoholic hepatitis with cirrhosis, the microbial tryptophan biosynthesis pathway was significantly increased (Fig. 4A). Despite the increase in the functional capacity of microbial tryptophan biosynthesis, metabolomic analysis revealed that serum levels of tryptophan and tryptophan‐derived metabolites, and indole‐3‐propionic acid, were decreased in patients with alcoholic hepatitis with cirrhosis (Fig. 4B). Fecal levels of indole‐3‐propionic acid (Fig. 4C) and indole‐3‐lactic acid (Fig. 4D) were also significantly reduced in the patients with alcoholic hepatitis with cirrhosis. There was no significant difference in the serum level of tryptophan, indole‐3‐propionic acid, and indole‐3‐lactic acid when comparing patients with alcoholic hepatitis without cirrhosis and alcoholic hepatitis patients with cirrhosis (Fig. 4B‐D). To test the diagnostic value of microbial pathways and serum metabolites, we used either serum metabolites or microbial tryptophan biosynthesis pathway alone, or a combination of both serum metabolites and microbial tryptophan biosynthesis pathway, as predictors of 30‐day mortality. The resulting scores for AUROC ranged from 0.479 to 0.571 when using single serum metabolites or microbial tryptophan biosynthesis pathway alone, whereas using a combination of microbial tryptophan biosynthesis, indole‐3‐propionic acid and tryptophan, the AUROC score achieved an AUROC of 0.891 (Fig. 4E). To assess generalizability of our logistic regression analysis results, leave‐one‐out cross validation was performed on this patient cohort. The bias‐corrected prediction error was 0.124 for our multi‐omics model using the leave‐one‐out cross validation method, which is small relative to 0.891. Thus, our prediction results based on the entire sample seem reliable.

### Decreased Serum Isoleucine Is Associated With Increased MELD Score

**Fig. 5:**
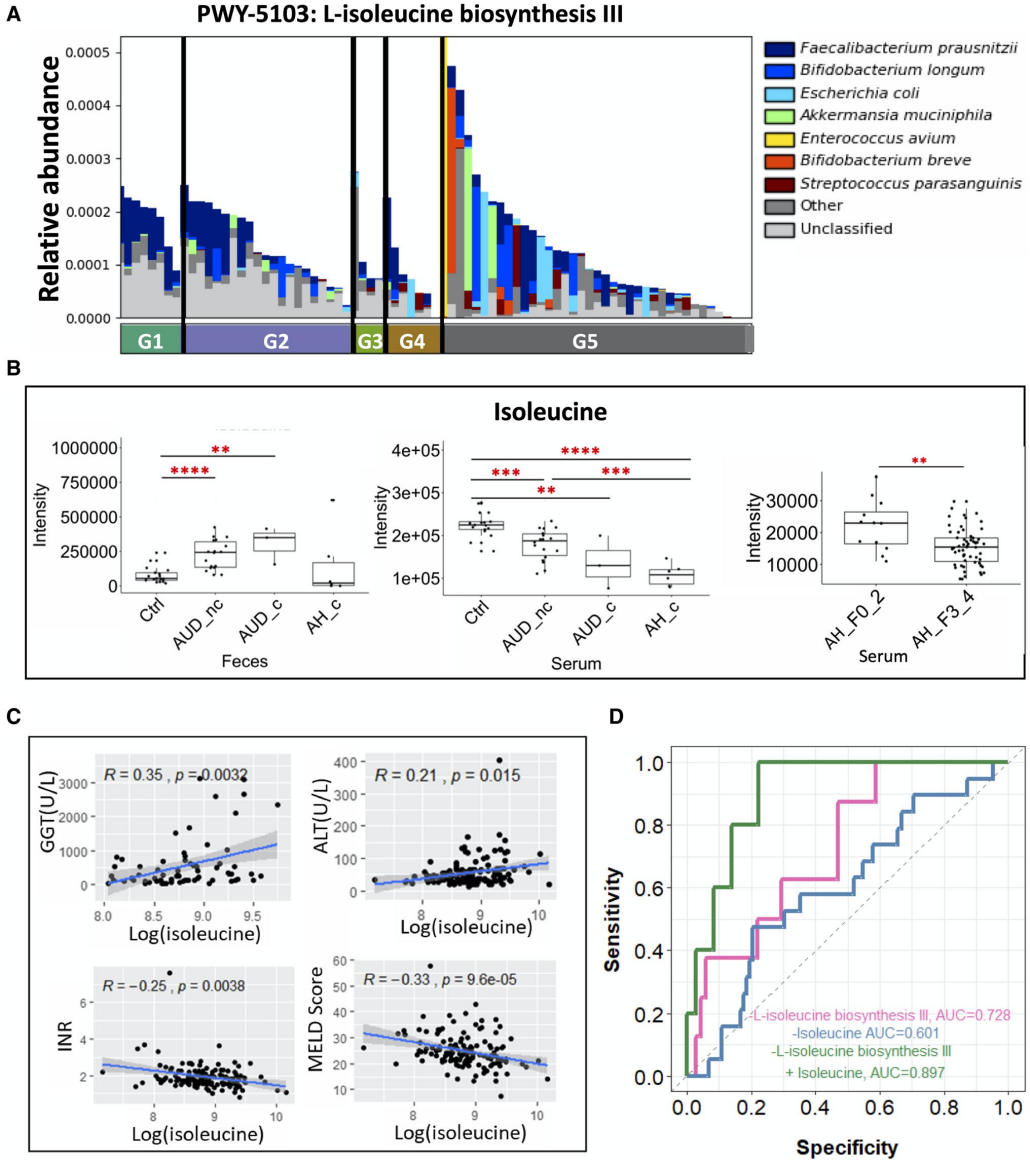
Isoleucine metabolism. (A) Relative abundance of microbial L‐isoleucine biosynthesis III in five groups. (B) Fecal and serum level of isoleucine. (C) Spearman correlation between isoleucine level (log transformation) in the serum of patients with alcoholic hepatitis and gamma‐glutamyl‐transferase, alanine aminotransferase, INR, and MELD score. (D) AUROC using different predictors related the isoleucine metabolism. Alive group, n = 38; deceased group, n = 5. *P < 0.05, **P < 0.01, ***P < 0.001, ****P < 0.0001. Abbreviations: ALT, alanine aminotransferase; GGT, gamma‐glutamyl‐transferase.

In addition to the aromatic amino acids, the gut microbiota are also essential factors for the supply of branch chain amino acids such as isoleucine to the host.^(^ ^27^ ^)^ The microbial isoleucine biosynthesis pathway was not significantly different in the five groups (Fig. 5A), whereas the serum level of isoleucine was decreased in patients with alcoholic hepatitis with cirrhosis in the exploratory cohort (Fig. 5B). In addition, the serum level of isoleucine was significantly decreased in patients with alcoholic hepatitis with cirrhosis compared with patients with alcoholic hepatitis without cirrhosis (Fig. 5B). The serum level of isoleucine was positively correlated with gamma‐glutamyl‐transferase , alanine aminotransferase, and negatively correlated with international normalized ratio (INR) and MELD score (Fig. 5C). Multi‐omics integration of the isoleucine biosynthesis pathway and serum level of isoleucine as a predictor of 30‐day mortality in patients with alcoholic hepatitis achieved an AUROC score of 0.897, which performed better than using single omics data as predictors (Fig. 5D). The bias‐corrected prediction error was 0.107 using the leave‐one‐out cross validation method.

### Increased Serum Methionine Is Associated With Lower 30‐Day Survival

**Fig. 6:**
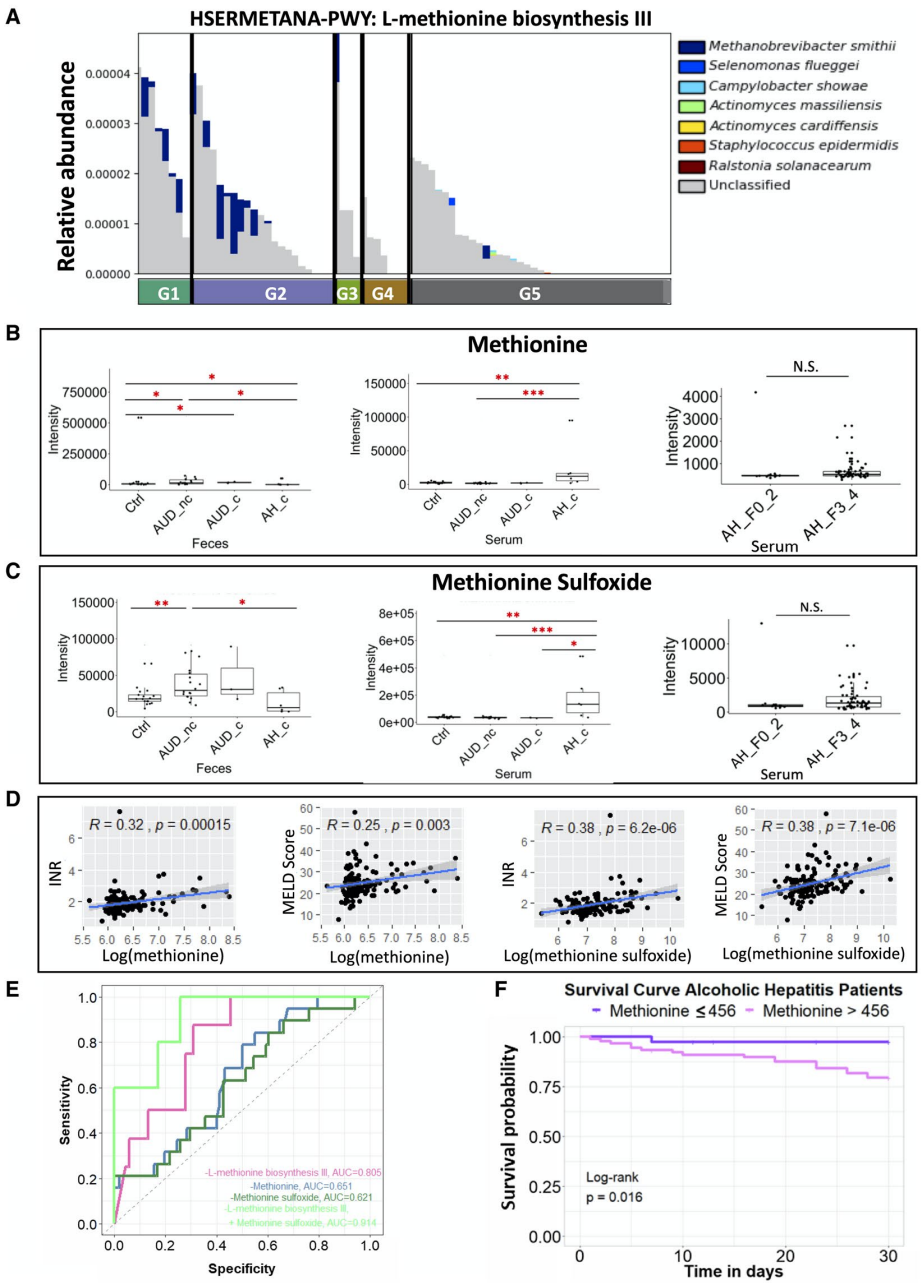
Methionine metabolism. (A) Relative abundance of microbial L‐methionine biosynthesis III in five groups. G5 versus G1: *P* = 0.004; G5 versus G2: *P* = 0.013. Fecal and serum level of methionine (B) and methionine sulfoxide (C). (D) Spearman correlation between methionine level (log transformation) in the serum (left panel) or methionine sulfoxide level (log transformation) in the serum (right panel) of patients with alcoholic hepatitis with INR and MELD score. (E) AUROC using different predictors related to methionine metabolism. Alive group, n = 38; deceased group, n = 5. (F) Kaplan‐Meier curve of 30‐day mortality for patients with alcoholic hepatitis. Patients were grouped according to their serum levels of methionine. Patients lost to follow‐up were censored at the time they were last seen alive. *P* > 0.05, *P < 0.05, **P < 0.01, ***P < 0.001, ****P < 0.0001.

As an essential amino acid containing sulfur, methionine is the precursor of various other metabolites, including quorum‐sensing molecules such as acyl homoserine lactones and autoinducer‐2, which are key molecules for the communication in bacteria.^(^ ^28^ ^)^ In patients with alcoholic hepatitis with cirrhosis, the microbial methionine biosynthesis was reduced (Fig. 6A). Consistently, fecal methionine and methionine sulfoxide were also decreased in patients with alcoholic hepatitis with cirrhosis. Meanwhile, serum methionine and methionine sulfoxide were increased in patients with alcoholic hepatitis with cirrhosis (Fig. 6B,C). In the validation cohort of patients with alcoholic hepatitis, no significant difference was found in the serum level of methionine and methionine sulfoxide between patients with alcoholic hepatitis without cirrhosis and with cirrhosis (Fig. 6B,C). Higher serum level of methionine or methionine sulfoxide was associated with increased INR and MELD score (Fig. 6D). A combination of microbial methionine biosynthesis and serum level of methionine sulfoxide as a predictor for 30‐day mortality achieved an AUROC score of 0.914, with a bias‐corrected prediction error of 0.077 using the leave‐one‐out cross validation method. Again, multi‐omics performed better than the microbial methionine biosynthesis pathway (AUROC = 0.805), serum level of methionine (AUROC = 0.651), or methionine sulfoxide alone (AUROC = 0.621) (Fig. 6E). Using the maximally selected rank method, we found that patients with serum methionine greater than an intensity of 456 had a significantly lower 30‐day survival, compared with patients with serum methionine level lower than or equal to 456, with a hazard ratio of 8.01 under univariate Cox regression analysis and a hazard ratio of 12.09 under multivariate Cox regression when adjusted for MELD score, antibiotics, steroids, and pentoxifylline treatment (Fig. 6F and Supporting Table S11).

### Higher Serum Urea Is Associated With Lower 30‐Day Survival

**Fig. 7:**
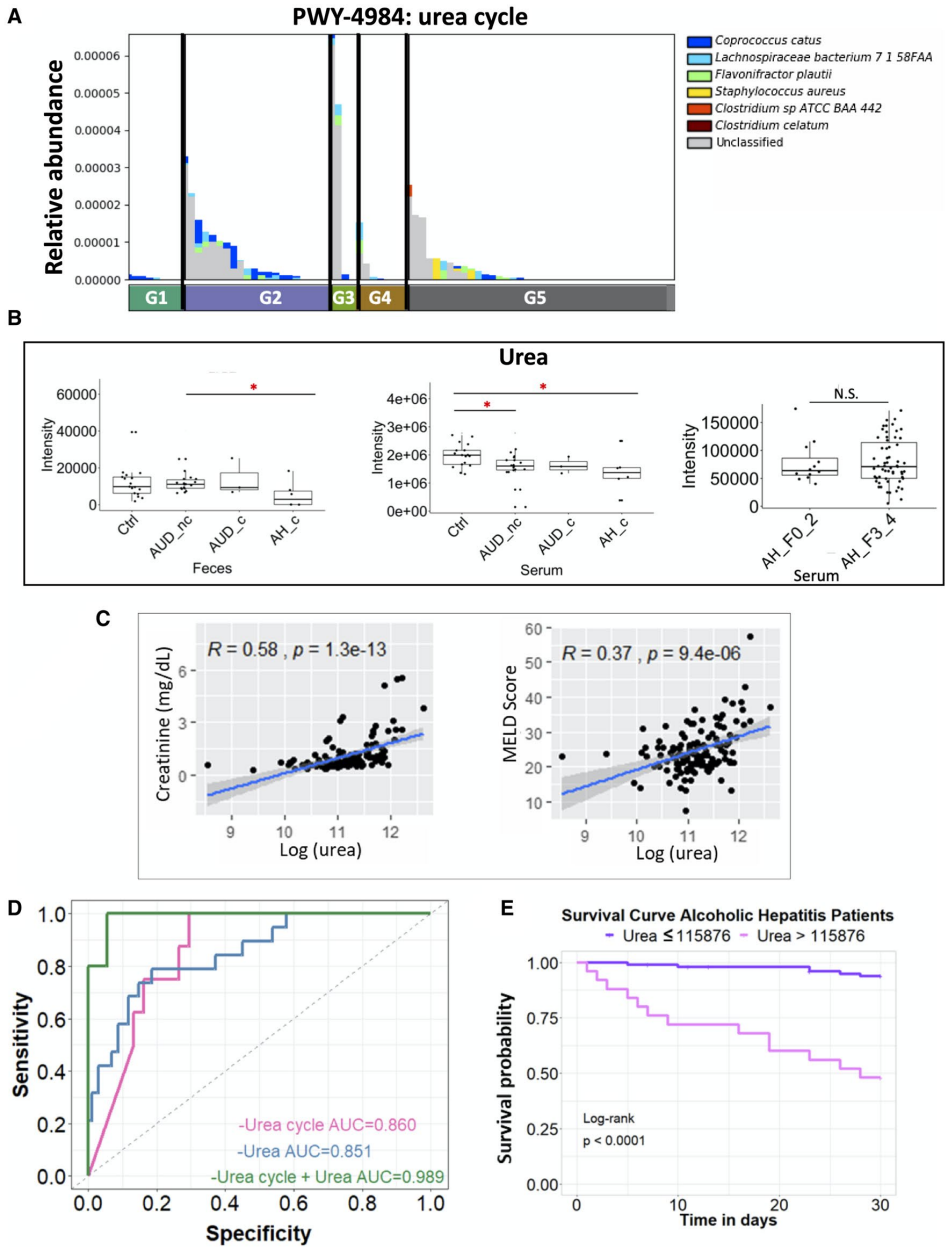
Urea cycle. (A) Relative abundance of microbial urea cycle pathway in five groups. G2 versus G1: *P* = 0.038; G3 versus G1: *P* = 0.022; G5 versus G2: *P* = 0.012; G5 versus G3: *P* = 0.029. (B) Fecal and serum level of urea. (C) Spearman correlation between urea level (log transformation) in the serum of patients with alcoholic hepatitis with creatinine and MELD score. (D) Receiver operating curves with AUC using different predictors related to urea cycle. Alive group, n = 38; deceased group, n = 5. (E) Kaplan‐Meier curve of 30‐day mortality for patients with alcoholic hepatitis. Patients were grouped according to their serum levels of urea. Patients lost to follow‐up were censored at the time they were last seen alive. *P* > 0.05, *P < 0.05, **P < 0.01, ***P < 0.001.

Hepatocytes metabolize ammonia into urea, which is then excreted as waste product into the urine and transported into the intestine to be hydrolyzed by bacterial urease into carbon dioxide and ammonia.^(^ ^28^ ^)^ In patients with alcoholic hepatitis with cirrhosis, microbial urea cycle was significantly increased (Fig. 7A), meanwhile the fecal and serum levels of urea were decreased (Fig. 7B). No significant difference was found in the serum level of urea between patients with alcoholic hepatitis without cirrhosis and with cirrhosis (Fig. 7B). In patients with alcoholic hepatitis, the higher serum level of urea is significantly correlated with elevated creatinine and higher MELD score (Fig. 7C). Integration of microbial urea cycle and the serum level of urea as a predictor for 30‐day mortality achieved an AUROC score of 0.989, with a bias‐corrected prediction error of 0.083 using the leave‐one‐out cross validation method, which was higher than AUROC for microbial urea cycle (0.860) or for the serum level of urea (0.851) (Fig. 7D). Using the maximally selected rank method, we found that patients with serum level of urea greater than an intensity of 115876 had a significantly lower 30‐day survival, compared to those with serum urea level lower than or equal to 115876, with a hazard ratio of 11.92 using univariate Cox regression and 10.72 using multivariate Cox regression when adjusted for MELD score, treatment of antibiotics, steroids, and pentoxifylline (Fig. 7E and Supporting Table S11).

## Discussion

There are several limitations of this study. Most patients had cirrhosis in the alcoholic hepatitis cohort, whereas only three patients with alcohol use disorder had F3‐4 disease. This small sample size is one limitation of this study. In addition, our study lacks additional control cohorts such as patients with alcoholic cirrhosis without hepatitis and patients with cirrhosis without alcoholic hepatitis. Lack of additional controls is another limitation of this study. Although we did not stratify our patients by gender, we performed MaAsLin2 analysis to reveal significant microbes, microbial pathways (Supporting Table S12), and fecal and serum metabolites (Supporting Table S13) associated with gender. Despite these limitations, our study showed that multi‐omics integration is a promising approach to predict the short‐term mortality in patients with alcoholic hepatitis.

As a key metabolic function of the liver, urea synthesis plays a regulatory role in nitrogen homeostasis. Impaired urea cycle in fatty liver disease has been reported previously.^(^ ^29^, ^30^, ^31^, ^32^ ^)^ The capacity of urea synthesis is decreased in patients with compromised liver function but increases in patients with inflammation.^(^ ^33^ ^)^ Interestingly, both mechanisms are involved in alcoholic hepatitis. As a result of two opposite effects, decreased capacity for urea synthesis has been reported in patients with alcoholic hepatitis.^(^ ^33^ ^)^ Consistently, a decrease of urea was observed in the serum samples of patients with alcoholic hepatitis compared with controls in our patient cohort, which is likely due to the decrease in hepatic urea synthesis. In line with the decrease of hepatic urea synthesis in the cohort of patients with alcoholic hepatitis, the contribution of microbial urea synthesis to the total urea pool might increase. Within the cohort of patients with alcoholic hepatitis, increased serum urea correlates with disease outcome, which could reflect its role as biomarker of kidney function. When combining the microbial urea cycle pathway with the serum level of urea to predict the 30‐day mortality in patients with alcoholic hepatitis, the AUROC score is as high as 0.989. Engineering the gut microbiome has been proposed for the treatment of hyperammonemia.^(^ ^34^ ^)^

Although our prediction model was not validated in an independent patient cohort, the results from this patient cohort were validated using the leave‐one‐out cross validation method, and we reported the bias‐corrected prediction error. Our results showed that when integrating microbial pathways with the serum metabolites, the performance of prediction was better than using traditional metabolites alone, with bias‐corrected prediction errors ranging from 0.077 to 0.124 to predict the 30‐day mortality. Our study provides valuable information for the identification of new drug targets and development of personalized therapeutic strategies for patients with alcohol‐related liver disease.

## Supporting information

Fig S1

Fig S2

Fig S3

Fig S4

Fig S5

Supplementary Material
