# Supplementary figures and images for: Functional Microbiomics Reveals Alterations of the Gut Microbiome and Host Co‐Metabolism in Patients With Alcoholic Hepatitis
Source: Hepatol Commun. 2020 Jun 19;4(8):1168–82. doi: 10.1002/hep4.1537 (PMC7395072; doi:10.1002/hep4.1537)

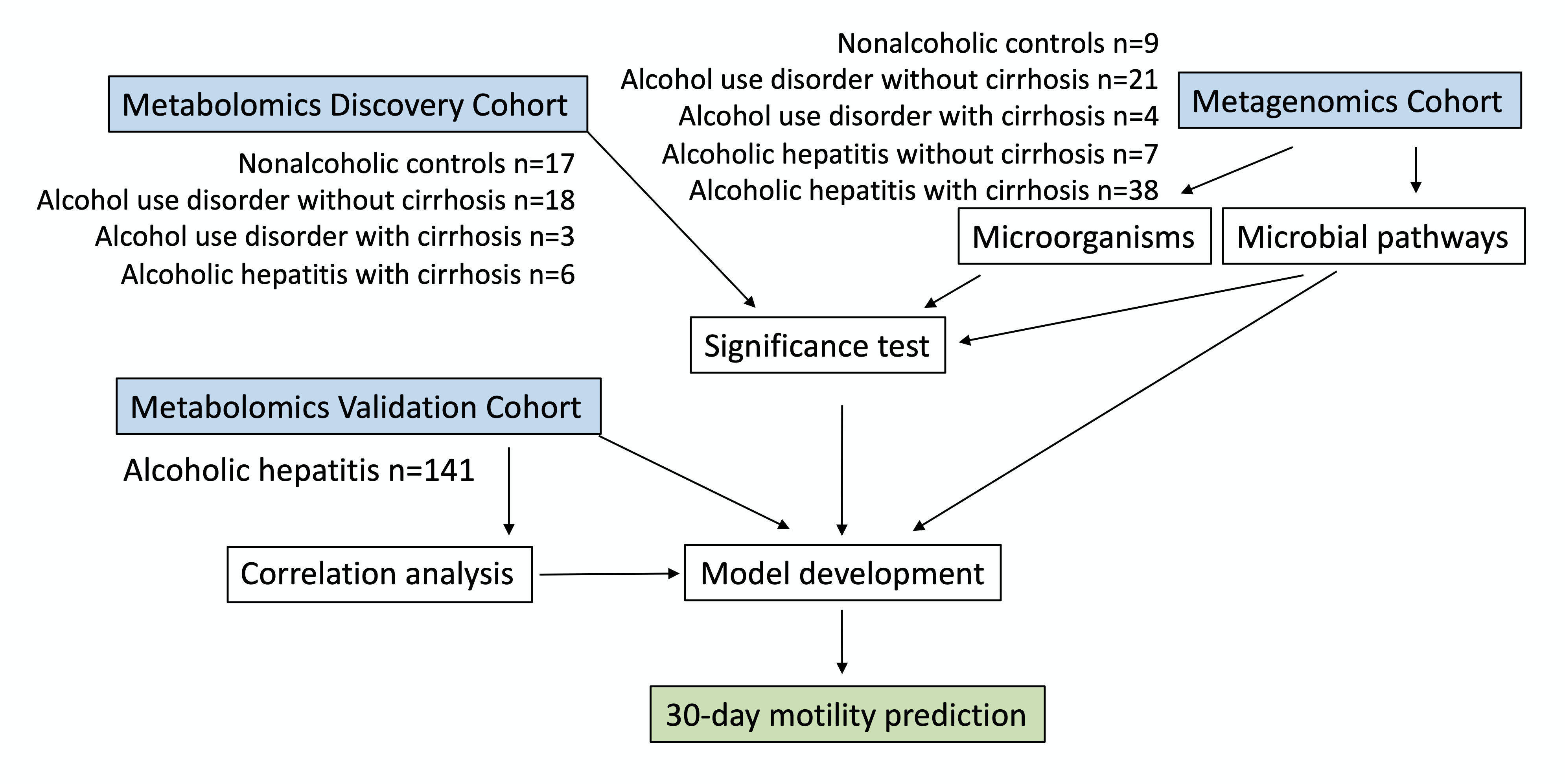

Supplement: Supplementary file 1 — Fig S1 [file HEP4-4-1168-s001.tif]

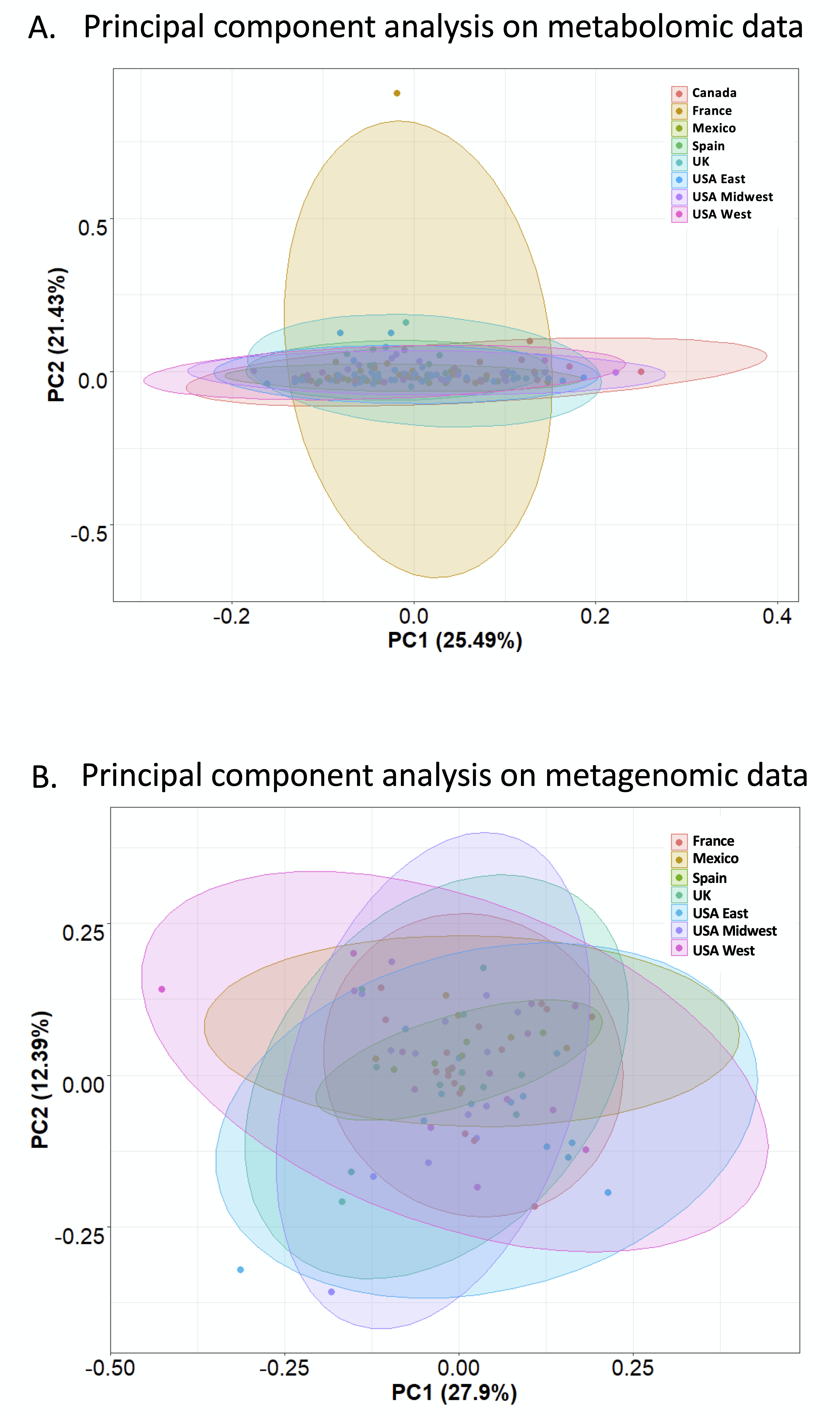

Supplement: Supplementary file 2 — Fig S2 [file HEP4-4-1168-s002.tif]

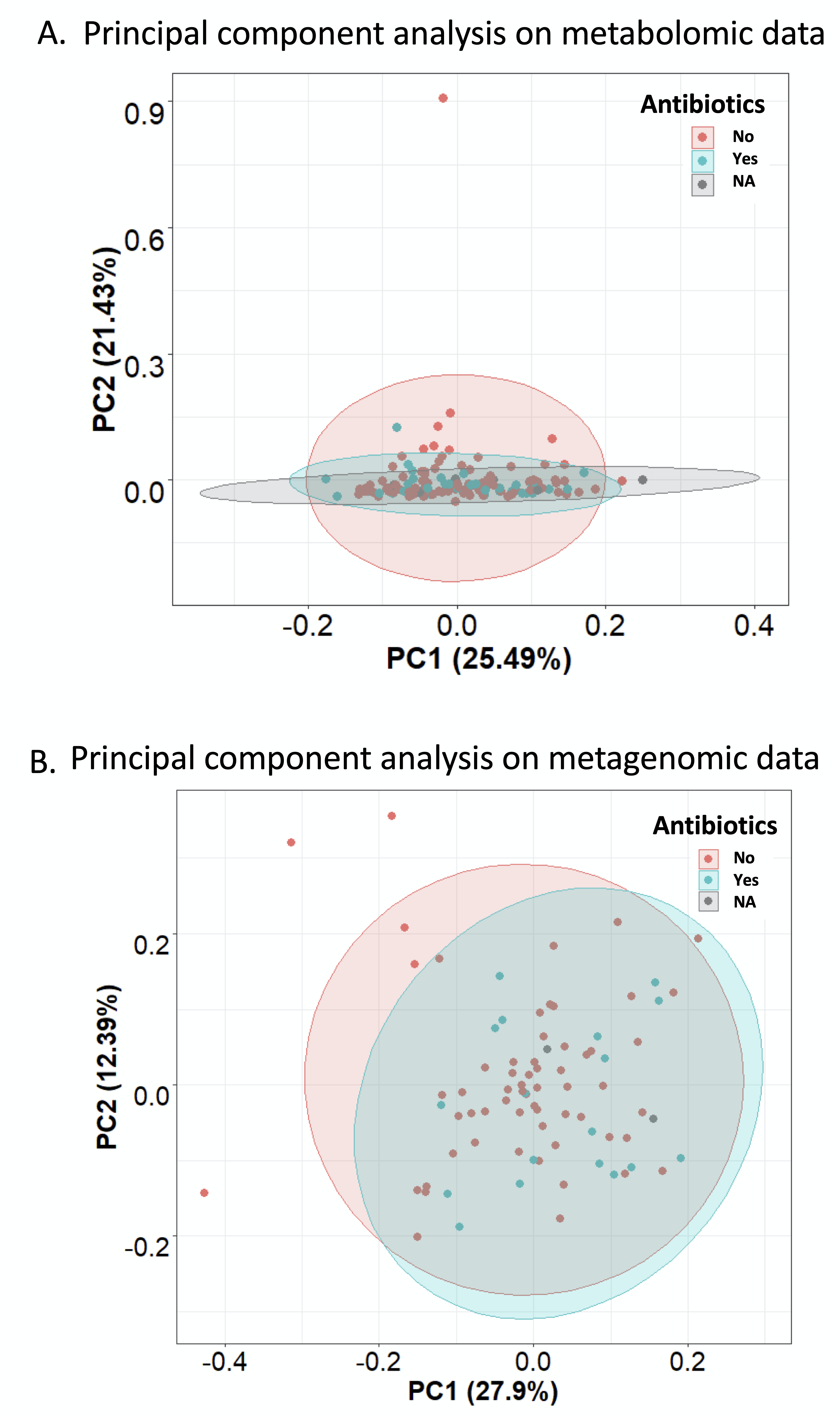

Supplement: Supplementary file 3 — Fig S3 [file HEP4-4-1168-s003.tif]

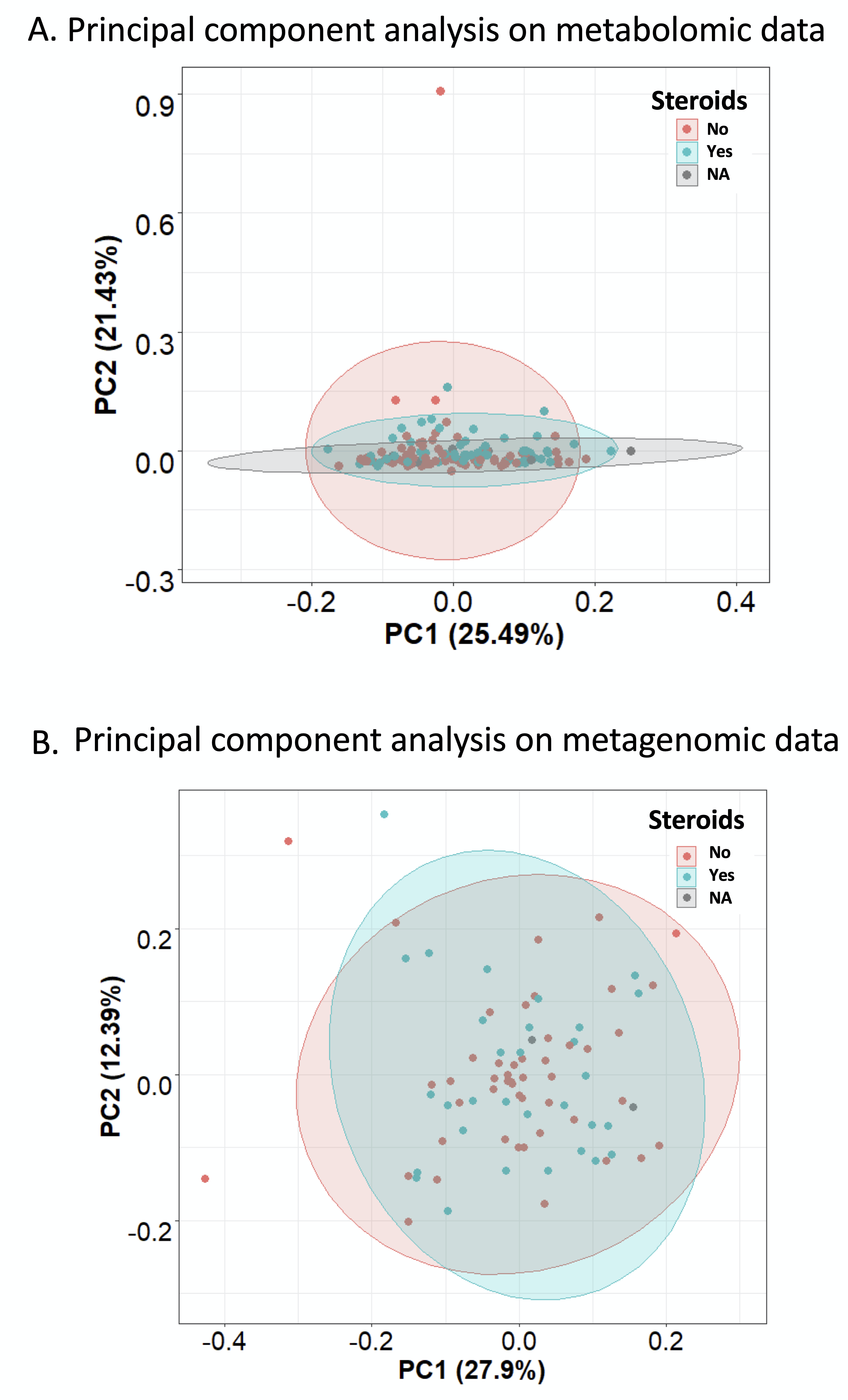

Supplement: Supplementary file 4 — Fig S4 [file HEP4-4-1168-s004.tif]

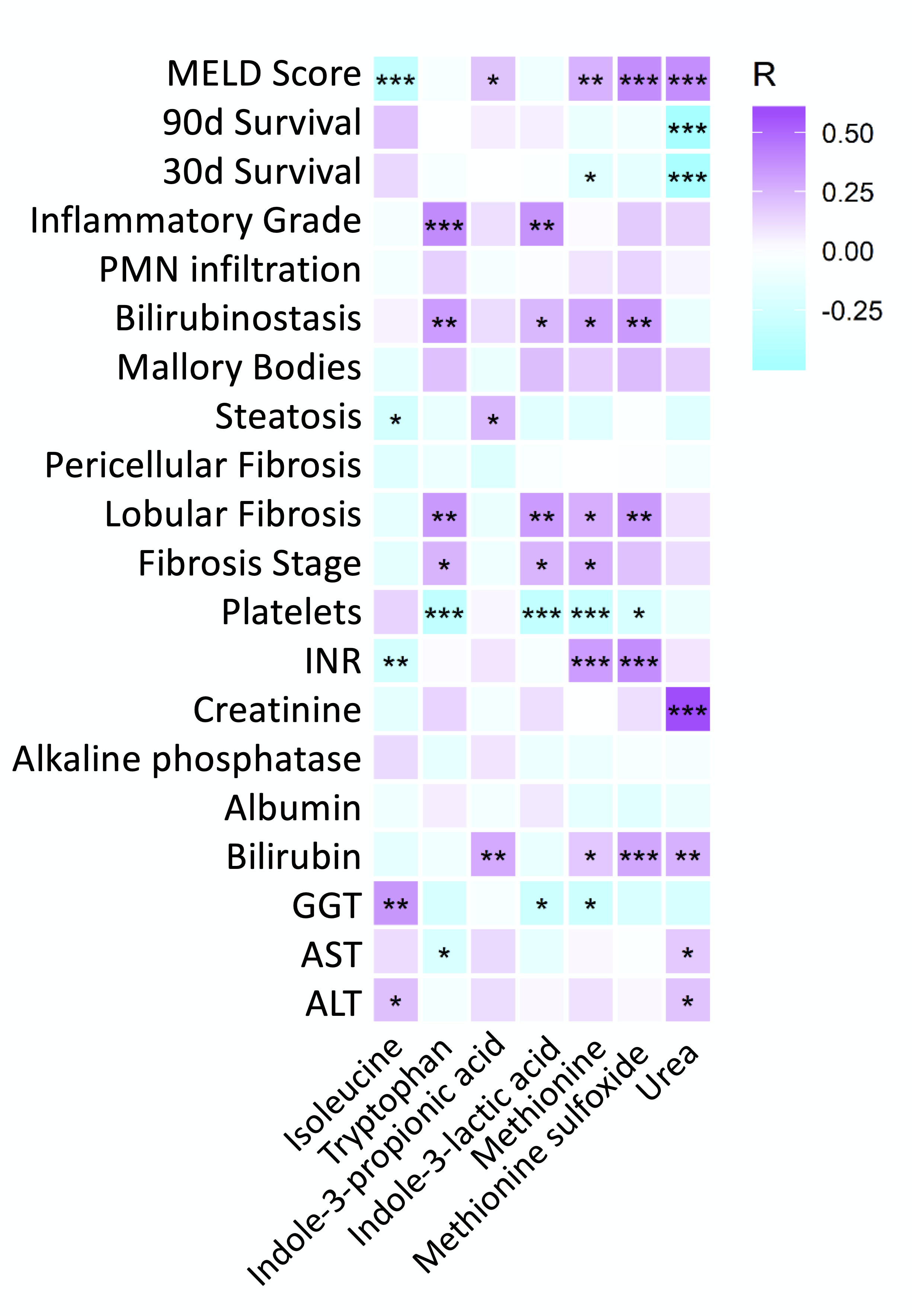

Supplement: Supplementary file 5 — Fig S5 [file HEP4-4-1168-s005.tif]
