# Supplementary material for: Functional Microbiomics Reveals Alterations of the Gut Microbiome and Host Co‐Metabolism in Patients With Alcoholic Hepatitis
Source: Hepatol Commun. 2020 Jun 19;4(8):1168–82. doi: 10.1002/hep4.1537 (PMC7395072; doi:10.1002/hep4.1537)
Supplement: Supplementary file 6 — Supplementary Material [file HEP4-4-1168-s006.docx]

**Supporting Information**

**Functional microbiomics reveals alterations of the gut microbiome and host co-metabolism in alcoholic hepatitis patients**

Table of Contents

[Table S1. Subject characteristics for the metabolomic analysis 2](#_Toc30792117)

[Table S2. Characteristics of alcoholic hepatitis patients for the metabolomic analysis 4](#_Toc30792118)

[Table S3. Subject characteristics for the metagenomic analysis 6](#_Toc30792119)

[Table S4. Characteristics of alcoholic hepatitis patients for the metagenomic analysis 8](#_Toc30792120)

[Table S5. Detected microbial pathways in MetaCyc database 9](#_Toc30792121)

[Table S6. LEfSe analysis of microbial pathways 20](#_Toc30792122)

[Table S7. Association between alcohol intake and microbes and microbial pathways 25](#_Toc30792123)

[Table S8. Annotated metabolites in serum and fecal samples 26](#_Toc30792124)

[Table S9. Significantly altered metabolites in alcoholic hepatitis patients with cirrhosis compared with alcohol use disorder patients with cirrhosis 40](#_Toc30792125)

[Table S10. Significantly different metabolites in alcholic hepatitis patients with cirrhosis compared with alcoholic hepatitis without cirrhosis 43](#_Toc30792126)

[Table S11. High levels of methionine and urea are associated with increased 30-Day mortality in patients with alcoholic hepatitis 45](#_Toc30792127)

[Table S12. Association of gender and microbes and microbial pathways 46](#_Toc30792128)

[Table S13. Association between gender and serum and fecal metabolites 47](#_Toc30792129)

[Supplementary Figure Legends 49](#_Toc30792130)

# Table S1. Subject characteristics for the metabolomic analysis

|  | Non-alcoholic Controls | Alcohol Use Disorder without cirrhosis | Alcohol Use disorder with cirhosis | Alcoholic Hepatitis  First Set | Alcoholic Hepatitis  Second Set | *P-value*  (a) | *P*-value  (b) |
| --- | --- | --- | --- | --- | --- | --- | --- |
| Clinical parameter |  |  |  |  |  |  |  |
| Total n | 17 | 18 | 3 | 13 | 141 |  |  |
| Age, years, n=190 | 39 (27-71) | 39 (27-53) | 47 (37-53) | 58 (40-75) | 49 (26-70) | **01** | **0.010** |
| Body Mass Index (BMI), kg/m², n=167 | 22 (19-29) | 22 (18-25) | 20 (20-24) | 27 (22-37) | 27 (16-45) | **03** | 0.904 |
| Gender (male), n (%), n=191 | 14 (82) | 14 (78) | 2 (67) | 10 (77) | 92 (66) | 0.937 | 0.936 |
|  |  |  |  |  |  |  |  |
| Laboratory parameter |  |  |  |  |  |  |  |
| Albumin (g/dl), n=164 |  | 4.6 (4.2-5.2) | 4.6 (3.9-5.1) | 2.5 (1.8-3.5) | 2.5 (1.3-4.2) | **<01** | 0.701 |
| Alkaline phosphatase (U/l), n=167 |  | 63 (38-101) | 88 (75-99) | 268 (81-1153) | 164 (21-511) | **<01** | 0.177 |
| ALT (U/l), n=169 |  | 26 (11-133) | 77 (46-143) | 63 (28-106) | 44 (15-404) | **08** | 0.090 |
| AST (U/l), n=169 |  | 29 (15-155) | 93 (84-283) | 167 (69-290) | 127 (34-1858) | **<01** | 0.211 |
| Total bilirubin (mg/dl), n=169 |  | 0.4 (0.2-1.1) | 0.6 (0.5-0.7) | 11.1 (3.1-36.2) | 13.8 (2.5-51.8) | **<01** | 0.405 |
| GGT (U/l), n=99 |  | 36 (4-952) | 489 (14-558) | 309 (70-2860) | 257 (33-3145) | **06** | 0.858 |
| Platelet counts (x10 9/l), n=168 |  | 237 (148-434) | 80 (21-163) | 115 (60-350) | 123 (12-447) | **<01** | 0.777 |
| Prothrombin time, s, n=131 |  |  |  | 23 (11-61) | 22 (9-141) |  | 0.949 |
| Creatinine (mg/dl), n=148 |  | 0.7 (0.5-1.2) | 0.8 (0.8-0.8) | 0.9 (0.4-2.0) | 1.0 (1.0-5.5) | 0.374 | 0.884 |
| Sodium (mEq/L), n=148 |  |  |  | 132 (118-138) | 134 (106-148) |  | 0.262 |
| INR, n=168 |  | 1.0 (0.9-1.2) | 0.8 (0.8-0.9) | 1.6 (1.1-2.5) | 1.8 (0.8-7.6) | **<01** | 0.161 |
| FIB-4, n=167 |  | 1.0 (0.4-3.1) | 9.1 (5.4-21.4) | 10.3 (4.2-27.7) | 7.5 (0.7-73.9) | **<01** | 0.406 |
| FIB-4 > 3.25 (F3-F4), n (%) |  | 0 (0) | 3 (100) | 13 (100) | 120 (85) |  |  |

Values are presented as median and range in parentheses ( ). The number of patients for which the respective data was available is indicated in the first column. In blank cells, patients from the respective group were not counted to missing numbers. *P*-value (a): comparison of non-alcohlic controls, alcohol use disorder and alcoholic hepatitis patients first set. *P*-value (b): comparison between alcoholic hepatitis patients first set and second set. Kruskal-Wallis test was used for three group comparision. Mann-Whitney test was used for two group comparision. Tukey and Kramer (Nemenyi) post-hoc test was used for pairwise comparisons. Bold font indicates significance (p-value < 0.05). ALT, alanine aminotransferase; AST, aspartate aminotransferase; BMI, body mass index; GGT, gamma-glutamyl-transferase; INR, international normalized ratio.

Pairwise multiple comparisons (only significant p-values were listed). Age: AH_c v.s. AUD_nc: p-value=01, AH_c v.s. Control: p-value=0.010; BMI: AH_c v.s. AUD_nc: p-value=02, AH_c v.s. Control: p-value=0.027; Albumin: AH_c v.s. AUD_c: p-value=0.031, AH_c v.s. AUC_nc: p-value<01; Alkaline phosphatase: AH_c v.s. AUD_nc: p-value<01; ALT: AH_c v.s. AUD_nc: p-value=0.024; AST: AH_c v.s AUD_nc: p-value<01; Total bilirubin: AH_c v.s. AUD_nc: p-value<01; GGT: AH_c v.s. AUD_nc: p-value=04; Platelet counts: AH_c v.s. AUD_nc: p-value<01; INR: AH_c v.s. AUD_c: p-value<01, AH_c v.s. AUD_nc: p-value<01; FIB_4: AH_c v.s. AUD_nc: p-value<01, AUD_c v.s. AUD_nc: p-value=0.018.

# Table S2. Characteristics of alcoholic hepatitis patients for the metabolomic analysis

| Treatment at admission | First Set n=13 | | | | Second Set n=141 | |
| --- | --- | --- | --- | --- | --- | --- |
| Steroids, n (%) | n=13 | 0 (0) | | | n=134 | 58 (43) |
| Pentoxifylline, n (%) | n=13 | 0 (0) | | | n=116 | 7 (6) |
| Steroids and pentoxifylline both, n (%) | n=13 | 0 (0) | | | n=116 | 1 (1) |
| Antibiotics, n (%) | n=13 | 0 (0) | | | n=134 | 36 (27) |
| Proton pump inhibitors, n (%) | n=9 | 1 (11) | | | n=65 | 8 (12) |
| Infection at admission, n (%) | n=12 | 1 (8) | | | n=104 | 23 (22) |
|  |  |  | | |  |  |
| Clinical scores and outcome |  |  | | |  |  |
| Model for end-stage liver disease (MELD) | n=12 | 22 (12-30) | | | n=135 | 24 (8-58) |
| MELD>21, n (%) | n=12 | 7 (58) | | | n=135 | 102 (76) |
| 30 day mortality, n (%) | n=12 | 0 (0) | | | n=120 | 19 (16) |
| 90 day mortality, n (%) | n=8 | 2 (25) | | | n=92 | 33 (36) |
|  |  |  | | |  |  |
| Histology |  |  | | |  |  |
| Liver biopsy available, n (%) | n=13 |  | 7 (54) | | n=138 | 74 (54) |
| Stage of fibrosis, n (%) | n=7 | 0 | | 0 (0) | n=71 | 1 (1) |
|  |  | 1 | | 0 (0) |  | 2 (3) |
|  |  | 2 | | 1 (14) |  | 9 (13) |
|  |  | 3 | | 1 (14) |  | 15 (21) |
|  |  | 4 | | 5 (72) |  | 44 (62) |
| Lobular fibrosis, n (%) | n=7 | 0 | | 0 (0) | n=70 | 5 (7) |
|  |  | 1 | | 1 (14) |  | 11 (16) |
|  |  | 2 | | 2 (29) |  | 5 (7) |
|  |  | 3 | | 4 (57) |  | 49 (70) |
| Pericellular fibrosis, n (%) | n=7 | 0 | | 2 (29) | n=71 | 17 (24) |
|  |  | 1 | | 5 (71) |  | 54 (76) |
| Grade of steatosis, n (%) | n=7 | 1 | | 3 (43) | n=72 | 25 (35) |
|  |  | 2 | | 3 (43) |  | 19 (26) |
|  |  | 3 | | 1 (14) |  | 28 (39) |
| Mallory bodies, n (%) | n=7 | 0 | | 1 (14) | n=70 | 13 (19) |
|  |  | 1 | | 6 (86) |  | 57 (81) |
| Bilirubinostasis, n (%) | n=6 | 0 | | 1 (17) | n=71 | 26 (37) |
|  |  | 1 | | 4 (66) |  | 28 (39) |
|  |  | 2 | | 0 (0) |  | 4 (6) |
|  |  | 3 | | 1 (17) |  | 13 (18) |
| Ballooning, n (%) | n=7 | 0 | | 7 (100) | n=70 | 42 (60) |
|  |  | 1 | | 0 (0) |  | 28 (40) |
| Giant mitochondria, n (%) | n=5 | 0 | | 5 (100) | n=71 | 54 (76) |
|  |  | 1 | | 0 (0) |  | 17 (24) |
| PMN infiltration, n (%) | n=7 | 0 | | 1 (14) | n=71 | 17 (24) |
|  |  | 1 | | 4 (57) |  | 32 (45) |
|  |  | 2 | | 2 (29) |  | 22 (31) |
| Inflammatory grade, n (%) | n=7 | 0 | | 0 (0) | n=72 | 18 (25) |
|  |  | 1 | | 6 (86) |  | 47 (65) |
|  |  | 2 | | 1 (14) |  | 7 (10) |

Clinical characteristics of alcoholic hepatitis patients. Values are presented as median and range in brackets. The total number of patients for which the respective data was available is indicated on the left in the colume of each dataset. In blank cells, patients from the respective group were not counted to missing numbers. Fibrosis stage, 0 no fibrosis, 1 portal fibrosis, 2 expansive periportal fibrosis, 3 bridging fibrosis, 4 cirrhosis. Lobular fibrosis, 0 no fibrosis, 1 zone 3 (centrilobular) fibrosis, 2 zone 2+3 (midzonal) fibrosis, 3 panlobular fibrosis. Pericellular fibrosis, 0 absent, 1 present. Steatosis, 1 mild < 33%, 2 moderate < 33-66%, 3 marked > 66%. Mallory bodies, 0 absent, 1 present. Bilirubinostasis, 0 no, 1 hepato-canalicular, 2 cholangiolar, 3 both. Ballooning, 0 occasional hepatocellular, 1 marked hepatocellular, 2 none present. Megamitochondria, 0 absent, 1 present. PMN infiltration, 0 no, 1 mild, 2 severe. Inflammation, 0 no, 1 mild, 2 severe. PMN, polymorphonuclear infiltration. MELD: model for end-stage liver disease.

# Table S3. Subject characteristics for the metagenomic analysis

|  | G1: Non-alcoholic Controls | G2: Alcohol Use Disorder without cirrhosis | G3: Alcohol Use Disorder with cirrhosis | G4: Alcoholic Hepatitis without cirrhosis | G5: Alcoholic Hepatitis with cirrhosis | *P*-value |
| --- | --- | --- | --- | --- | --- | --- |
| Clinical parameter |  |  |  |  |  |  |
| Total n | 9 | 21 | 4 | 7 | 38 |  |
| Age (years), n=79 | 51 (27-71) | 42 (27-63) | 50 (37-58) | 53 (48-61) | 51 (30-75) | **0.013** |
| Body Mass Index (BMI), kg/m², n=70 | 23 (19-29) | 22 (18-25) | 22 (18-24) | 28 (21-32) | 26 (19-41) | **<01** |
| Male gender, n (%), n=79 | 7 (78) | 17(81) | 3 (75) | 5 (71) | 26 (68) | 0.877 |
|  |  |  |  |  |  |  |
| Laboratory parameter |  |  |  |  |  |  |
| Albumin (g/dl), n=65 |  | 4.5 (4.1-5.2) | 4.3 (3.1-5.1) | 2.9 (2.0-3.8) | 2.3 (1.3-4.1) | **<01** |
| Alkaline phosphatase (U/l), n=49 |  | 54 (33-68) | 225 (225-225) | 206 (84-456) | 176 (21-418) | **<01** |
| ALT (U/l), n=70 |  | 28 (11-150) | 62 (43-143) | 61 (20-216) | 45 (15-127) | **<01** |
| AST (U/l), n=70 |  | 31 (15-155) | 94 (84-283) | 132 (74-283) | 130 (41-406) | **<01** |
| Total bilirubin (mg/dl), n=70 |  | 0.5 (0.2-1.1) | 0.65 (0.5-1.0) | 20.1 (3.1-27.5) | 16.5 (3.5-38.6) | **<01** |
| GGT (U/l), n=52 |  | 38 (4-952) | 318 (14-558) | 173 (33-3632) | 132 (48-830) | **<01** |
| Platelet counts (x10 9/l), n=68 |  | 223 (141-434) | 122 (21-256) | 141 (21-194) | 114 (22-333) | **<01** |
| Prothrombin time, s, n=41 |  |  |  | 17 (11-60) | 22 (15-61) | 0.146 |
| Creatinine (mg/dl), n=70 |  | 0.7 (0.5-1.2) | 0.8 (0.7-0.9) | 1.0 (0.8-5.6) | 0.8 (0.3-8.1) | **<01** |
| Sodium (mEq/L), n=45 |  |  |  | 134 (122-137) | 132 (118-208) | 0.937 |
| INR, n=70 |  | 0.9 (0.9-1.2) | 0.9 (0.9-1.0) | 1.4 (1.0-3.5) | 1.7 (1.3-3.7) | **<01** |
| FIB-4, n=68 |  | 1.2 (0.4-3.1) | 7.2 (3.3-21.4) | 7.3 (4.2-42.8) | 10.6 (1.4-66.3) | **<01** |
| FIB-4 > 3.25 (F3-F4), n (%) |  | 0 (0) | 4 (100) | 7 (100) | 33 (87) |  |

Values are presented as median and range in brackets. The total number of patients for which the respective data was available is indicated in the first column. In blank cells, patients from the respective group were not counted to missing numbers. Kruskal-Wallis test was used for three group comparision. Tukey and Kramer (Nemenyi) post-hoc test was used for pairwise comparisons. Mann-Whitney test was used for two group comparision. Bold font indicates significance (*P*-value < 0.05). ALT, alanine aminotransferase; AST, aspartate aminotransferase; INR, international normalized ratio; GGT, gamma-glutamyl-transferase; BMI, body mass index.

Pairwise multiple comparisons (only significant p-values were listed). Age: G2 v.s. G4: p-value=0.046, G2 v.s. G5: p-value=0.014; BMI: G2 v.s. G4: p-value=0.031, G2 v.s. G5: p-value<01; Albumin: G1 v.s. G4: p-value<01, G1 v.s. G5: p-value<01, G2 v.s. G4: p-value=0.024, G2 v.s. G5: p-value<01; Alkaline phosphatase: G1 v.s. G5: p-value<0.01, G2 v.s. G4: p-value=0.049; G2 v.s. G5: p-value<01; ALT: G1 v.s. G2: p-value<01; G1 v.s. G4: p-value=0.02; G1 v.s. G5: p-value<01; AST: G1 v.s. G2: p-value=02; G1 v.s. G4: p-value=06, G1 v.s. G5: p-value<01; Total bilirubin: G1 v.s. G2: p-value<01, G1 v.s. G3: p-value<01, G1 v.s. G5: p-value=0.013; G2 v.s. G4: p-value=03; G2 v.s. G5: p-value<01; GGT: G1 v.s. G2: p-value<01, G1 v.s. G3: p-value=0.01, G1 v.s. G4: p-value=07, G2 v.s. G5: p-value<01; Platelet counts: G1 v.s. G2: p-value<01; G1 v.s. G4: p-value<01; G1 v.s. G5: p-value<01; Creatinine: G1 v.s. G2: p-value<01, G1 v.s. G3: p-value=0.03, G1 v.s. G5: p-value < 01; INR: G1 v.s. G2: p-value<01, G1 v.s. G3: p-value<01, G1 v.s. G5: p-value=0.016, G2 v.s. G4: p-value=0.018, G2 v.s. G5: p-value<01, G3 v.s. G5: p-value=08; FIB-4: G1 v.s. G2: p-value<01, G1 v.s. G5: p-value=02, G2 v.s. G3: p-value=0.041, G2 v.s. G4: p-value<01; G2 v.s. G5: p-value<01.

# Table S4. Characteristics of alcoholic hepatitis patients for the metagenomic analysis

| Treatment at admission | | |  |  | | Histology |  |  |
| --- | --- | --- | --- | --- | --- | --- | --- | --- |
| Steroids, n (%), n=79 | | |  | 31 (39) | | Pericellular fibrosis, n (%), n=45 | 0 | 11 (24) |
| Pentoxifyllin, n (%), n=65 | | |  | 6 (9) | |  | 1 | 34 (76) |
| Steroids and pentoxifylline, n (%), n=65 | | |  | 1 (2) | | Grade of steatosis, n (%), n=46 | 1 | 19 (41) |
| Antibiotics, n (%), n=79 | | |  | 18 (23) | |  | 2 | 14 (31) |
| Proton pump inhibitors, n (%), n=42 | | |  | 5 (12) | |  | 3 | 13 (28) |
| Infection at admission, n (%), n=68 | | |  | 13 (19) | | Mallory bodies, n (%), n=44 | 0 | 6 (14) |
|  | | |  |  | |  | 1 | 38 (86) |
| Clinical scores and outcome | | |  |  | | Bilirubinostasis, n (%), n=44 | 0 | 14 (32) |
| Model for end-stage liver disease (MELD), n=79 | | | | | 24 (12-46) |  | 1 | 21 (48) |
| MELD>21, n (%), n=79 | | |  | 62 (78) | |  | 2 | 2 (4) |
| 30 day mortality rate, n (%), n=76 | |  | | | 8 (11) |  | 3 | 7 (16) |
| 90 day mortality rate, n (%), n=56 | |  | | | 13 (23) | Ballooning, n (%), n=45 | 0 | 24 (53) |
|  | |  | | |  |  | 1 | 21 (47) |
| Histology | |  | | |  | Giant mitochondria, n (%), n=41 | 0 | 35 (85) |
| Liver biopsy available, n (%), n=81 | | | | | 47 (58) |  | 1 | 6 (15) |
| Stage of fibrosis, n (%), n=45 | | 0 | | | 2 (5) | PMN infiltration, n (%), n=45 | 0 | 10 (22) |
|  | | 1 | | | 0 (0) |  | 1 | 21 (47) |
|  | | 2 | | | 5 (11) |  | 2 | 14 (31) |
|  | | 3 | | | 6 (13) | Inflammatory grade, n (%), n=46 | 0 | 11 (24) |
|  | | 4 | | | 32 (71) |  | 1 | 26 (56) |
| Lobular fibrosis, n (%), n=44 | 0 | | | | 4 (9) |  | 2 | 9 (20) |
|  | 1 | | | | 5 (11) |  |  |  |
|  | 2 | | | | 2 (5) |  |  |  |
|  | 3 | | | | 33 (75) |  |  |  |

Clinical characteristics of 81 alcoholic hepatitis patients. Values are presented as median and range in brackets. The total number of patients for which the respective data was available is indicated in the first column. In blank cells, patients from the respective group were not counted to missing numbers. Fibrosis stage, 0 no fibrosis, 1 portal fibrosis, 2 expansive periportal fibrosis, 3 bridging fibrosis, 4 cirrhosis. Lobular fibrosis, 0 no fibrosis, 1 zone 3 (centrilobular) fibrosis, 2 zone 2+3 (midzonal) fibrosis, 3 panlobular fibrosis. Pericellular fibrosis, 0 absent, 1 present. Steatosis, 1 mild < 33%, 2 moderate < 33-66%, 3 marked > 66%. Mallory bodies, 0 absent, 1 present. Bilirubinostasis, 0 no, 1 hepato-canalicular, 2 cholangiolar, 3 both. Ballooning, 0 occasional hepatocellular, 1 marked hepatocellular, 2 none present. Megamitochondria, 0 absent, 1 present. PMN infiltration, 0 no, 1 mild, 2 severe. Inflammation, 0 no, 1 mild, 2 severe. PMN, polymorphonuclear infiltration. MELD: model for end-stage liver disease.

| Table S5. Detected microbial pathways in MetaCyc database | |
| --- | --- |
| Pathways | Full name |
| 1CMET2-PWY | 1CMET2-PWY: N10-formyl-tetrahydrofolate biosynthesis |
| 3-HYDROXYPHENYLACETATE-DEGRADATION-PWY | 3-HYDROXYPHENYLACETATE-DEGRADATION-PWY: 4-hydroxyphenylacetate degradation |
| 7ALPHADEHYDROX-PWY | 7ALPHADEHYDROX-PWY: cholate degradation (bacteria, anaerobic) |
| AEROBACTINSYN-PWY | AEROBACTINSYN-PWY: aerobactin biosynthesis |
| ALL-CHORISMATE-PWY | ALL-CHORISMATE-PWY: superpathway of chorismate metabolism |
| ALLANTOINDEG-PWY | ALLANTOINDEG-PWY: superpathway of allantoin degradation in yeast |
| ANAEROFRUCAT-PWY | ANAEROFRUCAT-PWY: homolactic fermentation |
| ANAGLYCOLYSIS-PWY | ANAGLYCOLYSIS-PWY: glycolysis III (from glucose) |
| ARG+POLYAMINE-SYN | ARG+POLYAMINE-SYN: superpathway of arginine and polyamine biosynthesis |
| ARGDEG-PWY | ARGDEG-PWY: superpathway of L-arginine, putrescine, and 4-aminobutanoate degradation |
| ARGININE-SYN4-PWY | ARGININE-SYN4-PWY: L-ornithine de novo biosynthesis |
| ARGORNPROST-PWY | ARGORNPROST-PWY: arginine, ornithine and proline interconversion |
| ARGSYN-PWY | ARGSYN-PWY: L-arginine biosynthesis I (via L-ornithine) |
| ARGSYNBSUB-PWY | ARGSYNBSUB-PWY: L-arginine biosynthesis II (acetyl cycle) |
| ARO-PWY | ARO-PWY: chorismate biosynthesis I |
| ASPASN-PWY | ASPASN-PWY: superpathway of L-aspartate and L-asparagine biosynthesis |
| AST-PWY | AST-PWY: L-arginine degradation II (AST pathway) |
| BIOTIN-BIOSYNTHESIS-PWY | BIOTIN-BIOSYNTHESIS-PWY: biotin biosynthesis I |
| BRANCHED-CHAIN-AA-SYN-PWY | BRANCHED-CHAIN-AA-SYN-PWY: superpathway of branched amino acid biosynthesis |
| CALVIN-PWY | CALVIN-PWY: Calvin-Benson-Bassham cycle |
| CATECHOL-ORTHO-CLEAVAGE-PWY | CATECHOL-ORTHO-CLEAVAGE-PWY: catechol degradation to &beta;-ketoadipate |
| CENTFERM-PWY | CENTFERM-PWY: pyruvate fermentation to butanoate |
| CITRULBIO-PWY | CITRULBIO-PWY: L-citrulline biosynthesis |
| COA-PWY-1 | COA-PWY-1: coenzyme A biosynthesis II (mammalian) |
| COA-PWY | COA-PWY: coenzyme A biosynthesis I |
| COBALSYN-PWY | COBALSYN-PWY: adenosylcobalamin salvage from cobinamide I |
| COLANSYN-PWY | COLANSYN-PWY: colanic acid building blocks biosynthesis |
| COMPLETE-ARO-PWY | COMPLETE-ARO-PWY: superpathway of aromatic amino acid biosynthesis |
| CRNFORCAT-PWY | CRNFORCAT-PWY: creatinine degradation I |
| DAPLYSINESYN-PWY | DAPLYSINESYN-PWY: L-lysine biosynthesis I |
| DENITRIFICATION-PWY | DENITRIFICATION-PWY: nitrate reduction I (denitrification) |
| DENOVOPURINE2-PWY | DENOVOPURINE2-PWY: superpathway of purine nucleotides de novo biosynthesis II |
| DTDPRHAMSYN-PWY | DTDPRHAMSYN-PWY: dTDP-L-rhamnose biosynthesis I |
| ECASYN-PWY | ECASYN-PWY: enterobacterial common antigen biosynthesis |
| ENTBACSYN-PWY | ENTBACSYN-PWY: enterobactin biosynthesis |
| FAO-PWY | FAO-PWY: fatty acid &beta;-oxidation I |
| FASYN-ELONG-PWY | FASYN-ELONG-PWY: fatty acid elongation -- saturated |
| FASYN-INITIAL-PWY | FASYN-INITIAL-PWY: superpathway of fatty acid biosynthesis initiation (E. coli) |
| FERMENTATION-PWY | FERMENTATION-PWY: mixed acid fermentation |
| FOLSYN-PWY | FOLSYN-PWY: superpathway of tetrahydrofolate biosynthesis and salvage |
| FUC-RHAMCAT-PWY | FUC-RHAMCAT-PWY: superpathway of fucose and rhamnose degradation |
| FUCCAT-PWY | FUCCAT-PWY: fucose degradation |
| GALACT-GLUCUROCAT-PWY | GALACT-GLUCUROCAT-PWY: superpathway of hexuronide and hexuronate degradation |
| GALACTARDEG-PWY | GALACTARDEG-PWY: D-galactarate degradation I |
| GALACTUROCAT-PWY | GALACTUROCAT-PWY: D-galacturonate degradation I |
| GLCMANNANAUT-PWY | GLCMANNANAUT-PWY: superpathway of N-acetylglucosamine, N-acetylmannosamine and N-acetylneuraminate degradation |
| GLUCARDEG-PWY | GLUCARDEG-PWY: D-glucarate degradation I |
| GLUCARGALACTSUPER-PWY | GLUCARGALACTSUPER-PWY: superpathway of D-glucarate and D-galactarate degradation |
| GLUCONEO-PWY | GLUCONEO-PWY: gluconeogenesis I |
| GLUCOSE1PMETAB-PWY | GLUCOSE1PMETAB-PWY: glucose and glucose-1-phosphate degradation |
| GLUCUROCAT-PWY | GLUCUROCAT-PWY: superpathway of &beta;-D-glucuronide and D-glucuronate degradation |
| GLUDEG-I-PWY | GLUDEG-I-PWY: GABA shunt |
| GLUTORN-PWY | GLUTORN-PWY: L-ornithine biosynthesis |
| GLYCOCAT-PWY | GLYCOCAT-PWY: glycogen degradation I (bacterial) |
| GLYCOGENSYNTH-PWY | GLYCOGENSYNTH-PWY: glycogen biosynthesis I (from ADP-D-Glucose) |
| GLYCOL-GLYOXDEG-PWY | GLYCOL-GLYOXDEG-PWY: superpathway of glycol metabolism and degradation |
| GLYCOLYSIS-E-D | GLYCOLYSIS-E-D: superpathway of glycolysis and Entner-Doudoroff |
| GLYCOLYSIS-TCA-GLYOX-BYPASS | GLYCOLYSIS-TCA-GLYOX-BYPASS: superpathway of glycolysis, pyruvate dehydrogenase, TCA, and glyoxylate bypass |
| GLYCOLYSIS | GLYCOLYSIS: glycolysis I (from glucose 6-phosphate) |
| GLYOXYLATE-BYPASS | GLYOXYLATE-BYPASS: glyoxylate cycle |
| GOLPDLCAT-PWY | GOLPDLCAT-PWY: superpathway of glycerol degradation to 1,3-propanediol |
| HCAMHPDEG-PWY | HCAMHPDEG-PWY: 3-phenylpropanoate and 3-(3-hydroxyphenyl)propanoate degradation to 2-oxopent-4-enoate |
| HEME-BIOSYNTHESIS-II | HEME-BIOSYNTHESIS-II: heme biosynthesis I (aerobic) |
| HEMESYN2-PWY | HEMESYN2-PWY: heme biosynthesis II (anaerobic) |
| HEXITOLDEGSUPER-PWY | HEXITOLDEGSUPER-PWY: superpathway of hexitol degradation (bacteria) |
| HISDEG-PWY | HISDEG-PWY: L-histidine degradation I |
| HISTSYN-PWY | HISTSYN-PWY: L-histidine biosynthesis |
| HOMOSER-METSYN-PWY | HOMOSER-METSYN-PWY: L-methionine biosynthesis I |
| HSERMETANA-PWY | HSERMETANA-PWY: L-methionine biosynthesis III |
| ILEUSYN-PWY | ILEUSYN-PWY: L-isoleucine biosynthesis I (from threonine) |
| KDO-NAGLIPASYN-PWY | KDO-NAGLIPASYN-PWY: superpathway of (Kdo)2-lipid A biosynthesis |
| KETOGLUCONMET-PWY | KETOGLUCONMET-PWY: ketogluconate metabolism |
| LACTOSECAT-PWY | LACTOSECAT-PWY: lactose and galactose degradation I |
| LEU-DEG2-PWY | LEU-DEG2-PWY: L-leucine degradation I |
| LIPASYN-PWY | LIPASYN-PWY: phospholipases |
| LPSSYN-PWY | LPSSYN-PWY: superpathway of lipopolysaccharide biosynthesis |
| MET-SAM-PWY | MET-SAM-PWY: superpathway of S-adenosyl-L-methionine biosynthesis |
| METHANOGENESIS-PWY | METHANOGENESIS-PWY: methanogenesis from H2 and CO2 |
| METHGLYUT-PWY | METHGLYUT-PWY: superpathway of methylglyoxal degradation |
| METSYN-PWY | METSYN-PWY: L-homoserine and L-methionine biosynthesis |
| NAD-BIOSYNTHESIS-II | NAD-BIOSYNTHESIS-II: NAD salvage pathway II |
| NADSYN-PWY | NADSYN-PWY: NAD biosynthesis II (from tryptophan) |
| NAGLIPASYN-PWY | NAGLIPASYN-PWY: lipid IVA biosynthesis |
| NONMEVIPP-PWY | NONMEVIPP-PWY: methylerythritol phosphate pathway I |
| NONOXIPENT-PWY | NONOXIPENT-PWY: pentose phosphate pathway (non-oxidative branch) |
| OANTIGEN-PWY | OANTIGEN-PWY: O-antigen building blocks biosynthesis (E. coli) |
| ORNARGDEG-PWY | ORNARGDEG-PWY: superpathway of L-arginine and L-ornithine degradation |
| ORNDEG-PWY | ORNDEG-PWY: superpathway of ornithine degradation |
| P105-PWY | P105-PWY: TCA cycle IV (2-oxoglutarate decarboxylase) |
| P108-PWY | P108-PWY: pyruvate fermentation to propanoate I |
| P122-PWY | P122-PWY: heterolactic fermentation |
| P124-PWY | P124-PWY: Bifidobacterium shunt |
| P125-PWY | P125-PWY: superpathway of (R,R)-butanediol biosynthesis |
| P161-PWY | P161-PWY: acetylene degradation |
| P162-PWY | P162-PWY: L-glutamate degradation V (via hydroxyglutarate) |
| P163-PWY | P163-PWY: L-lysine fermentation to acetate and butanoate |
| P164-PWY | P164-PWY: purine nucleobases degradation I (anaerobic) |
| P165-PWY | P165-PWY: superpathway of purines degradation in plants |
| P185-PWY | P185-PWY: formaldehyde assimilation III (dihydroxyacetone cycle) |
| P221-PWY | P221-PWY: octane oxidation |
| P23-PWY | P23-PWY: reductive TCA cycle I |
| P261-PWY | P261-PWY: coenzyme M biosynthesis I |
| P4-PWY | P4-PWY: superpathway of L-lysine, L-threonine and L-methionine biosynthesis I |
| P42-PWY | P42-PWY: incomplete reductive TCA cycle |
| P441-PWY | P441-PWY: superpathway of N-acetylneuraminate degradation |
| P461-PWY | P461-PWY: hexitol fermentation to lactate, formate, ethanol and acetate |
| P562-PWY | P562-PWY: myo-inositol degradation I |
| PANTO-PWY | PANTO-PWY: phosphopantothenate biosynthesis I |
| PANTOSYN-PWY | PANTOSYN-PWY: pantothenate and coenzyme A biosynthesis I |
| PENTOSE-P-PWY | PENTOSE-P-PWY: pentose phosphate pathway |
| PEPTIDOGLYCANSYN-PWY | PEPTIDOGLYCANSYN-PWY: peptidoglycan biosynthesis I (meso-diaminopimelate containing) |
| PHOSLIPSYN-PWY | PHOSLIPSYN-PWY: superpathway of phospholipid biosynthesis I (bacteria) |
| PHOTOALL-PWY | PHOTOALL-PWY: oxygenic photosynthesis |
| POLYAMINSYN3-PWY | POLYAMINSYN3-PWY: superpathway of polyamine biosynthesis II |
| POLYAMSYN-PWY | POLYAMSYN-PWY: superpathway of polyamine biosynthesis I |
| POLYISOPRENSYN-PWY | POLYISOPRENSYN-PWY: polyisoprenoid biosynthesis (E. coli) |
| PPGPPMET-PWY | PPGPPMET-PWY: ppGpp biosynthesis |
| PROPFERM-PWY | PROPFERM-PWY: L-alanine fermentation to propanoate and acetate |
| PROTOCATECHUATE-ORTHO-CLEAVAGE-PWY | PROTOCATECHUATE-ORTHO-CLEAVAGE-PWY: protocatechuate degradation II (ortho-cleavage pathway) |
| PRPP-PWY | PRPP-PWY: superpathway of histidine, purine, and pyrimidine biosynthesis |
| PWY-101 | PWY-101: photosynthesis light reactions |
| PWY-1042 | PWY-1042: glycolysis IV (plant cytosol) |
| PWY-1269 | PWY-1269: CMP-3-deoxy-D-manno-octulosonate biosynthesis I |
| PWY-1861 | PWY-1861: formaldehyde assimilation II (RuMP Cycle) |
| PWY-2201 | PWY-2201: folate transformations I |
| PWY-241 | PWY-241: C4 photosynthetic carbon assimilation cycle, NADP-ME type |
| PWY-2723 | PWY-2723: trehalose degradation V |
| PWY-2941 | PWY-2941: L-lysine biosynthesis II |
| PWY-2942 | PWY-2942: L-lysine biosynthesis III |
| PWY-3001 | PWY-3001: superpathway of L-isoleucine biosynthesis I |
| PWY-3502 | PWY-3502: superpathway of NAD biosynthesis in eukaryotes |
| PWY-3661 | PWY-3661: glycine betaine degradation I |
| PWY-3781 | PWY-3781: aerobic respiration I (cytochrome c) |
| PWY-3801 | PWY-3801: sucrose degradation II (sucrose synthase) |
| PWY-3841 | PWY-3841: folate transformations II |
| PWY-4041 | PWY-4041: gamma-glutamyl cycle |
| PWY-4242 | PWY-4242: pantothenate and coenzyme A biosynthesis III |
| PWY-4321 | PWY-4321: L-glutamate degradation IV |
| PWY-4361 | PWY-4361: S-methyl-5-thio-&alpha;-D-ribose 1-phosphate degradation |
| PWY-4702 | PWY-4702: phytate degradation I |
| PWY-4981 | PWY-4981: L-proline biosynthesis II (from arginine) |
| PWY-4984 | PWY-4984: urea cycle |
| PWY-5004 | PWY-5004: superpathway of L-citrulline metabolism |
| PWY-5005 | PWY-5005: biotin biosynthesis II |
| PWY-5022 | PWY-5022: 4-aminobutanoate degradation V |
| PWY-5028 | PWY-5028: L-histidine degradation II |
| PWY-5030 | PWY-5030: L-histidine degradation III |
| PWY-5044 | PWY-5044: purine nucleotides degradation I (plants) |
| PWY-5067 | PWY-5067: glycogen biosynthesis II (from UDP-D-Glucose) |
| PWY-5079 | PWY-5079: L-phenylalanine degradation III |
| PWY-5080 | PWY-5080: very long chain fatty acid biosynthesis I |
| PWY-5083 | PWY-5083: NAD/NADH phosphorylation and dephosphorylation |
| PWY-5088 | PWY-5088: L-glutamate degradation VIII (to propanoate) |
| PWY-5097 | PWY-5097: L-lysine biosynthesis VI |
| PWY-5100 | PWY-5100: pyruvate fermentation to acetate and lactate II |
| PWY-5101 | PWY-5101: L-isoleucine biosynthesis II |
| PWY-5103 | PWY-5103: L-isoleucine biosynthesis III |
| PWY-5104 | PWY-5104: L-isoleucine biosynthesis IV |
| PWY-5121 | PWY-5121: superpathway of geranylgeranyl diphosphate biosynthesis II (via MEP) |
| PWY-5129 | PWY-5129: sphingolipid biosynthesis (plants) |
| PWY-5136 | PWY-5136: fatty acid &beta;-oxidation II (peroxisome) |
| PWY-5138 | PWY-5138: unsaturated, even numbered fatty acid &beta;-oxidation |
| PWY-5154 | PWY-5154: L-arginine biosynthesis III (via N-acetyl-L-citrulline) |
| PWY-5173 | PWY-5173: superpathway of acetyl-CoA biosynthesis |
| PWY-5177 | PWY-5177: glutaryl-CoA degradation |
| PWY-5179 | PWY-5179: toluene degradation V (aerobic) (via toluene-cis-diol) |
| PWY-5180 | PWY-5180: toluene degradation I (aerobic) (via o-cresol) |
| PWY-5181 | PWY-5181: toluene degradation III (aerobic) (via p-cresol) |
| PWY-5182 | PWY-5182: toluene degradation II (aerobic) (via 4-methylcatechol) |
| PWY-5188 | PWY-5188: tetrapyrrole biosynthesis I (from glutamate) |
| PWY-5189 | PWY-5189: tetrapyrrole biosynthesis II (from glycine) |
| PWY-5198 | PWY-5198: factor 420 biosynthesis |
| PWY-5265 | PWY-5265: peptidoglycan biosynthesis II (staphylococci) |
| PWY-5304 | PWY-5304: superpathway of sulfur oxidation (Acidianus ambivalens) |
| PWY-5306 | PWY-5306: superpathway of thiosulfate metabolism (Desulfovibrio sulfodismutans) |
| PWY-5345 | PWY-5345: superpathway of L-methionine biosynthesis (by sulfhydrylation) |
| PWY-5347 | PWY-5347: superpathway of L-methionine biosynthesis (transsulfuration) |
| PWY-5367 | PWY-5367: petroselinate biosynthesis |
| PWY-5384 | PWY-5384: sucrose degradation IV (sucrose phosphorylase) |
| PWY-5415 | PWY-5415: catechol degradation I (meta-cleavage pathway) |
| PWY-5417 | PWY-5417: catechol degradation III (ortho-cleavage pathway) |
| PWY-5431 | PWY-5431: aromatic compounds degradation via &beta;-ketoadipate |
| PWY-5464 | PWY-5464: superpathway of cytosolic glycolysis (plants), pyruvate dehydrogenase and TCA cycle |
| PWY-5484 | PWY-5484: glycolysis II (from fructose 6-phosphate) |
| PWY-5505 | PWY-5505: L-glutamate and L-glutamine biosynthesis |
| PWY-5509 | PWY-5509: adenosylcobalamin biosynthesis from cobyrinate a,c-diamide I |
| PWY-5514 | PWY-5514: UDP-N-acetyl-D-galactosamine biosynthesis II |
| PWY-561 | PWY-561: superpathway of glyoxylate cycle and fatty acid degradation |
| PWY-5651 | PWY-5651: L-tryptophan degradation to 2-amino-3-carboxymuconate semialdehyde |
| PWY-5656 | PWY-5656: mannosylglycerate biosynthesis I |
| PWY-5659 | PWY-5659: GDP-mannose biosynthesis |
| PWY-5667 | PWY-5667: CDP-diacylglycerol biosynthesis I |
| PWY-5675 | PWY-5675: nitrate reduction V (assimilatory) |
| PWY-5676 | PWY-5676: acetyl-CoA fermentation to butanoate II |
| PWY-5686 | PWY-5686: UMP biosynthesis |
| PWY-5690 | PWY-5690: TCA cycle II (plants and fungi) |
| PWY-5692 | PWY-5692: allantoin degradation to glyoxylate II |
| PWY-5695 | PWY-5695: urate biosynthesis/inosine 5'-phosphate degradation |
| PWY-5705 | PWY-5705: allantoin degradation to glyoxylate III |
| PWY-5723 | PWY-5723: Rubisco shunt |
| PWY-5747 | PWY-5747: 2-methylcitrate cycle II |
| PWY-5791 | PWY-5791: 1,4-dihydroxy-2-naphthoate biosynthesis II (plants) |
| PWY-5837 | PWY-5837: 1,4-dihydroxy-2-naphthoate biosynthesis I |
| PWY-5838 | PWY-5838: superpathway of menaquinol-8 biosynthesis I |
| PWY-5840 | PWY-5840: superpathway of menaquinol-7 biosynthesis |
| PWY-5845 | PWY-5845: superpathway of menaquinol-9 biosynthesis |
| PWY-5850 | PWY-5850: superpathway of menaquinol-6 biosynthesis I |
| PWY-5855 | PWY-5855: ubiquinol-7 biosynthesis (prokaryotic) |
| PWY-5856 | PWY-5856: ubiquinol-9 biosynthesis (prokaryotic) |
| PWY-5857 | PWY-5857: ubiquinol-10 biosynthesis (prokaryotic) |
| PWY-5860 | PWY-5860: superpathway of demethylmenaquinol-6 biosynthesis I |
| PWY-5861 | PWY-5861: superpathway of demethylmenaquinol-8 biosynthesis |
| PWY-5862 | PWY-5862: superpathway of demethylmenaquinol-9 biosynthesis |
| PWY-5863 | PWY-5863: superpathway of phylloquinol biosynthesis |
| PWY-5870 | PWY-5870: ubiquinol-8 biosynthesis (eukaryotic) |
| PWY-5871 | PWY-5871: ubiquinol-9 biosynthesis (eukaryotic) |
| PWY-5872 | PWY-5872: ubiquinol-10 biosynthesis (eukaryotic) |
| PWY-5873 | PWY-5873: ubiquinol-7 biosynthesis (eukaryotic) |
| PWY-5896 | PWY-5896: superpathway of menaquinol-10 biosynthesis |
| PWY-5897 | PWY-5897: superpathway of menaquinol-11 biosynthesis |
| PWY-5898 | PWY-5898: superpathway of menaquinol-12 biosynthesis |
| PWY-5899 | PWY-5899: superpathway of menaquinol-13 biosynthesis |
| PWY-5910 | PWY-5910: superpathway of geranylgeranyldiphosphate biosynthesis I (via mevalonate) |
| PWY-5913 | PWY-5913: TCA cycle VI (obligate autotrophs) |
| PWY-5918 | PWY-5918: superpathay of heme biosynthesis from glutamate |
| PWY-5920 | PWY-5920: superpathway of heme biosynthesis from glycine |
| PWY-5941 | PWY-5941: glycogen degradation II (eukaryotic) |
| PWY-5971 | PWY-5971: palmitate biosynthesis II (bacteria and plants) |
| PWY-5973 | PWY-5973: cis-vaccenate biosynthesis |
| PWY-5989 | PWY-5989: stearate biosynthesis II (bacteria and plants) |
| PWY-5994 | PWY-5994: palmitate biosynthesis I (animals and fungi) |
| PWY-6071 | PWY-6071: superpathway of phenylethylamine degradation |
| PWY-6113 | PWY-6113: superpathway of mycolate biosynthesis |
| PWY-6121 | PWY-6121: 5-aminoimidazole ribonucleotide biosynthesis I |
| PWY-6122 | PWY-6122: 5-aminoimidazole ribonucleotide biosynthesis II |
| PWY-6123 | PWY-6123: inosine-5'-phosphate biosynthesis I |
| PWY-6124 | PWY-6124: inosine-5'-phosphate biosynthesis II |
| PWY-6125 | PWY-6125: superpathway of guanosine nucleotides de novo biosynthesis II |
| PWY-6126 | PWY-6126: superpathway of adenosine nucleotides de novo biosynthesis II |
| PWY-6138 | PWY-6138: CMP-N-acetylneuraminate biosynthesis I (eukaryotes) |
| PWY-6147 | PWY-6147: 6-hydroxymethyl-dihydropterin diphosphate biosynthesis I |
| PWY-6151 | PWY-6151: S-adenosyl-L-methionine cycle I |
| PWY-6163 | PWY-6163: chorismate biosynthesis from 3-dehydroquinate |
| PWY-6165 | PWY-6165: chorismate biosynthesis II (archaea) |
| PWY-6167 | PWY-6167: flavin biosynthesis II (archaea) |
| PWY-6168 | PWY-6168: flavin biosynthesis III (fungi) |
| PWY-6174 | PWY-6174: mevalonate pathway II (archaea) |
| PWY-6182 | PWY-6182: superpathway of salicylate degradation |
| PWY-6185 | PWY-6185: 4-methylcatechol degradation (ortho cleavage) |
| PWY-621 | PWY-621: sucrose degradation III (sucrose invertase) |
| PWY-622 | PWY-622: starch biosynthesis |
| PWY-6263 | PWY-6263: superpathway of menaquinol-8 biosynthesis II |
| PWY-6270 | PWY-6270: isoprene biosynthesis I |
| PWY-6277 | PWY-6277: superpathway of 5-aminoimidazole ribonucleotide biosynthesis |
| PWY-6282 | PWY-6282: palmitoleate biosynthesis I (from (5Z)-dodec-5-enoate) |
| PWY-6284 | PWY-6284: superpathway of unsaturated fatty acids biosynthesis (E. coli) |
| PWY-6285 | PWY-6285: superpathway of fatty acids biosynthesis (E. coli) |
| PWY-6305 | PWY-6305: putrescine biosynthesis IV |
| PWY-6307 | PWY-6307: L-tryptophan degradation X (mammalian, via tryptamine) |
| PWY-6309 | PWY-6309: L-tryptophan degradation XI (mammalian, via kynurenine) |
| PWY-6313 | PWY-6313: serotonin degradation |
| PWY-6317 | PWY-6317: galactose degradation I (Leloir pathway) |
| PWY-6318 | PWY-6318: L-phenylalanine degradation IV (mammalian, via side chain) |
| PWY-6353 | PWY-6353: purine nucleotides degradation II (aerobic) |
| PWY-6385 | PWY-6385: peptidoglycan biosynthesis III (mycobacteria) |
| PWY-6386 | PWY-6386: UDP-N-acetylmuramoyl-pentapeptide biosynthesis II (lysine-containing) |
| PWY-6387 | PWY-6387: UDP-N-acetylmuramoyl-pentapeptide biosynthesis I (meso-diaminopimelate containing) |
| PWY-6396 | PWY-6396: superpathway of 2,3-butanediol biosynthesis |
| PWY-6435 | PWY-6435: 4-hydroxybenzoate biosynthesis V |
| PWY-6467 | PWY-6467: Kdo transfer to lipid IVA III (Chlamydia) |
| PWY-6470 | PWY-6470: peptidoglycan biosynthesis V (&beta;-lactam resistance) |
| PWY-6471 | PWY-6471: peptidoglycan biosynthesis IV (Enterococcus faecium) |
| PWY-6507 | PWY-6507: 4-deoxy-L-threo-hex-4-enopyranuronate degradation |
| PWY-6519 | PWY-6519: 8-amino-7-oxononanoate biosynthesis I |
| PWY-6527 | PWY-6527: stachyose degradation |
| PWY-6531 | PWY-6531: mannitol cycle |
| PWY-6545 | PWY-6545: pyrimidine deoxyribonucleotides de novo biosynthesis III |
| PWY-6549 | PWY-6549: L-glutamine biosynthesis III |
| PWY-6562 | PWY-6562: norspermidine biosynthesis |
| PWY-6588 | PWY-6588: pyruvate fermentation to acetone |
| PWY-6590 | PWY-6590: superpathway of Clostridium acetobutylicum acidogenic fermentation |
| PWY-6595 | PWY-6595: superpathway of guanosine nucleotides degradation (plants) |
| PWY-6596 | PWY-6596: adenosine nucleotides degradation I |
| PWY-6598 | PWY-6598: sciadonate biosynthesis |
| PWY-6606 | PWY-6606: guanosine nucleotides degradation II |
| PWY-6608 | PWY-6608: guanosine nucleotides degradation III |
| PWY-6609 | PWY-6609: adenine and adenosine salvage III |
| PWY-6612 | PWY-6612: superpathway of tetrahydrofolate biosynthesis |
| PWY-6628 | PWY-6628: superpathway of L-phenylalanine biosynthesis |
| PWY-6629 | PWY-6629: superpathway of L-tryptophan biosynthesis |
| PWY-6630 | PWY-6630: superpathway of L-tyrosine biosynthesis |
| PWY-6660 | PWY-6660: 2-heptyl-3-hydroxy-4(1H)-quinolone biosynthesis |
| PWY-6662 | PWY-6662: superpathway of quinolone and alkylquinolone biosynthesis |
| PWY-6676 | PWY-6676: superpathway of sulfide oxidation (phototrophic sulfur bacteria) |
| PWY-6690 | PWY-6690: cinnamate and 3-hydroxycinnamate degradation to 2-oxopent-4-enoate |
| PWY-6700 | PWY-6700: queuosine biosynthesis |
| PWY-6703 | PWY-6703: preQ0 biosynthesis |
| PWY-6708 | PWY-6708: ubiquinol-8 biosynthesis (prokaryotic) |
| PWY-6731 | PWY-6731: starch degradation III |
| PWY-6737 | PWY-6737: starch degradation V |
| PWY-6748 | PWY-6748: nitrate reduction VII (denitrification) |
| PWY-6749 | PWY-6749: CMP-legionaminate biosynthesis I |
| PWY-6763 | PWY-6763: salicortin biosynthesis |
| PWY-6785 | PWY-6785: hydrogen production VIII |
| PWY-6803 | PWY-6803: phosphatidylcholine acyl editing |
| PWY-6823 | PWY-6823: molybdenum cofactor biosynthesis |
| PWY-6837 | PWY-6837: fatty acid beta-oxidation V (unsaturated, odd number, di-isomerase-dependent) |
| PWY-6859 | PWY-6859: all-trans-farnesol biosynthesis |
| PWY-6876 | PWY-6876: isopropanol biosynthesis |
| PWY-6891 | PWY-6891: thiazole biosynthesis II (Bacillus) |
| PWY-6892 | PWY-6892: thiazole biosynthesis I (E. coli) |
| PWY-6895 | PWY-6895: superpathway of thiamin diphosphate biosynthesis II |
| PWY-6897 | PWY-6897: thiamin salvage II |
| PWY-6901 | PWY-6901: superpathway of glucose and xylose degradation |
| PWY-6936 | PWY-6936: seleno-amino acid biosynthesis |
| PWY-6969 | PWY-6969: TCA cycle V (2-oxoglutarate:ferredoxin oxidoreductase) |
| PWY-6981 | PWY-6981: chitin biosynthesis |
| PWY-7003 | PWY-7003: glycerol degradation to butanol |
| PWY-7007 | PWY-7007: methyl ketone biosynthesis |
| PWY-7013 | PWY-7013: L-1,2-propanediol degradation |
| PWY-7031 | PWY-7031: protein N-glycosylation (bacterial) |
| PWY-7039 | PWY-7039: phosphatidate metabolism, as a signaling molecule |
| PWY-7046 | PWY-7046: 4-coumarate degradation (anaerobic) |
| PWY-7094 | PWY-7094: fatty acid salvage |
| PWY-7111 | PWY-7111: pyruvate fermentation to isobutanol (engineered) |
| PWY-7115 | PWY-7115: C4 photosynthetic carbon assimilation cycle, NAD-ME type |
| PWY-7117 | PWY-7117: C4 photosynthetic carbon assimilation cycle, PEPCK type |
| PWY-7118 | PWY-7118: chitin degradation to ethanol |
| PWY-7165 | PWY-7165: L-ascorbate biosynthesis VI (engineered pathway) |
| PWY-7184 | PWY-7184: pyrimidine deoxyribonucleotides de novo biosynthesis I |
| PWY-7187 | PWY-7187: pyrimidine deoxyribonucleotides de novo biosynthesis II |
| PWY-7196 | PWY-7196: superpathway of pyrimidine ribonucleosides salvage |
| PWY-7197 | PWY-7197: pyrimidine deoxyribonucleotide phosphorylation |
| PWY-7198 | PWY-7198: pyrimidine deoxyribonucleotides de novo biosynthesis IV |
| PWY-7199 | PWY-7199: pyrimidine deoxyribonucleosides salvage |
| PWY-7200 | PWY-7200: superpathway of pyrimidine deoxyribonucleoside salvage |
| PWY-7204 | PWY-7204: pyridoxal 5'-phosphate salvage II (plants) |
| PWY-7208 | PWY-7208: superpathway of pyrimidine nucleobases salvage |
| PWY-7209 | PWY-7209: superpathway of pyrimidine ribonucleosides degradation |
| PWY-7210 | PWY-7210: pyrimidine deoxyribonucleotides biosynthesis from CTP |
| PWY-7211 | PWY-7211: superpathway of pyrimidine deoxyribonucleotides de novo biosynthesis |
| PWY-7218 | PWY-7218: photosynthetic 3-hydroxybutanoate biosynthesis (engineered) |
| PWY-7219 | PWY-7219: adenosine ribonucleotides de novo biosynthesis |
| PWY-7220 | PWY-7220: adenosine deoxyribonucleotides de novo biosynthesis II |
| PWY-7221 | PWY-7221: guanosine ribonucleotides de novo biosynthesis |
| PWY-7222 | PWY-7222: guanosine deoxyribonucleotides de novo biosynthesis II |
| PWY-7224 | PWY-7224: purine deoxyribonucleosides salvage |
| PWY-7228 | PWY-7228: superpathway of guanosine nucleotides de novo biosynthesis I |
| PWY-7229 | PWY-7229: superpathway of adenosine nucleotides de novo biosynthesis I |
| PWY-7234 | PWY-7234: inosine-5'-phosphate biosynthesis III |
| PWY-7235 | PWY-7235: superpathway of ubiquinol-6 biosynthesis (eukaryotic) |
| PWY-7237 | PWY-7237: myo-, chiro- and scillo-inositol degradation |
| PWY-7242 | PWY-7242: D-fructuronate degradation |
| PWY-7245 | PWY-7245: superpathway NAD/NADP - NADH/NADPH interconversion (yeast) |
| PWY-724 | PWY-724: superpathway of L-lysine, L-threonine and L-methionine biosynthesis II |
| PWY-7254 | PWY-7254: TCA cycle VII (acetate-producers) |
| PWY-7268 | PWY-7268: NAD/NADP-NADH/NADPH cytosolic interconversion (yeast) |
| PWY-7269 | PWY-7269: NAD/NADP-NADH/NADPH mitochondrial interconversion (yeast) |
| PWY-7270 | PWY-7270: L-methionine salvage cycle II (plants) |
| PWY-7279 | PWY-7279: aerobic respiration II (cytochrome c) (yeast) |
| PWY-7282 | PWY-7282: 4-amino-2-methyl-5-phosphomethylpyrimidine biosynthesis (yeast) |
| PWY-7283 | PWY-7283: wybutosine biosynthesis |
| PWY-7286 | PWY-7286: 7-(3-amino-3-carboxypropyl)-wyosine biosynthesis |
| PWY-7288 | PWY-7288: fatty acid &beta;-oxidation (peroxisome, yeast) |
| PWY-7290 | PWY-7290: Escherichia coli serotype O86 O-antigen biosynthesis |
| PWY-7294 | PWY-7294: xylose degradation IV |
| PWY-7315 | PWY-7315: dTDP-N-acetylthomosamine biosynthesis |
| PWY-7316 | PWY-7316: dTDP-N-acetylviosamine biosynthesis |
| PWY-7323 | PWY-7323: superpathway of GDP-mannose-derived O-antigen building blocks biosynthesis |
| PWY-7328 | PWY-7328: superpathway of UDP-glucose-derived O-antigen building blocks biosynthesis |
| PWY-7337 | PWY-7337: 10-cis-heptadecenoyl-CoA degradation (yeast) |
| PWY-7338 | PWY-7338: 10-trans-heptadecenoyl-CoA degradation (reductase-dependent, yeast) |
| PWY-7345 | PWY-7345: superpathway of anaerobic sucrose degradation |
| PWY-7357 | PWY-7357: thiamin formation from pyrithiamine and oxythiamine (yeast) |
| PWY-7371 | PWY-7371: 1,4-dihydroxy-6-naphthoate biosynthesis II |
| PWY-7373 | PWY-7373: superpathway of demethylmenaquinol-6 biosynthesis II |
| PWY-7374 | PWY-7374: 1,4-dihydroxy-6-naphthoate biosynthesis I |
| PWY-7383 | PWY-7383: anaerobic energy metabolism (invertebrates, cytosol) |
| PWY-7384 | PWY-7384: anaerobic energy metabolism (invertebrates, mitochondrial) |
| PWY-7385 | PWY-7385: 1,3-propanediol biosynthesis (engineered) |
| PWY-7388 | PWY-7388: octanoyl-[acyl-carrier protein] biosynthesis (mitochondria, yeast) |
| PWY-7389 | PWY-7389: superpathway of anaerobic energy metabolism (invertebrates) |
| PWY-7391 | PWY-7391: isoprene biosynthesis II (engineered) |
| PWY-7392 | PWY-7392: taxadiene biosynthesis (engineered) |
| PWY-7399 | PWY-7399: methylphosphonate degradation II |
| PWY-7400 | PWY-7400: L-arginine biosynthesis IV (archaebacteria) |
| PWY-7409 | PWY-7409: phospholipid remodeling (phosphatidylethanolamine, yeast) |
| PWY-7411 | PWY-7411: superpathway of phosphatidate biosynthesis (yeast) |
| PWY-7431 | PWY-7431: aromatic biogenic amine degradation (bacteria) |
| PWY-7446 | PWY-7446: sulfoglycolysis |
| PWY-7456 | PWY-7456: mannan degradation |
| PWY-7527 | PWY-7527: L-methionine salvage cycle III |
| PWY-7528 | PWY-7528: L-methionine salvage cycle I (bacteria and plants) |
| PWY-7539 | PWY-7539: 6-hydroxymethyl-dihydropterin diphosphate biosynthesis III (Chlamydia) |
| PWY-7546 | PWY-7546: diphthamide biosynthesis (eukaryotes) |
| PWY-7560 | PWY-7560: methylerythritol phosphate pathway II |
| PWY-7619 | PWY-7619: juniperonate biosynthesis |
| PWY-7663 | PWY-7663: gondoate biosynthesis (anaerobic) |
| PWY-7664 | PWY-7664: oleate biosynthesis IV (anaerobic) |
| PWY-821 | PWY-821: superpathway of sulfur amino acid biosynthesis (Saccharomyces cerevisiae) |
| PWY-841 | PWY-841: superpathway of purine nucleotides de novo biosynthesis I |
| PWY-922 | PWY-922: mevalonate pathway I |
| PWY0-1061 | PWY0-1061: superpathway of L-alanine biosynthesis |
| PWY0-1241 | PWY0-1241: ADP-L-glycero-&beta;-D-manno-heptose biosynthesis |
| PWY0-1261 | PWY0-1261: anhydromuropeptides recycling |
| PWY0-1277 | PWY0-1277: 3-phenylpropanoate and 3-(3-hydroxyphenyl)propanoate degradation |
| PWY0-1296 | PWY0-1296: purine ribonucleosides degradation |
| PWY0-1297 | PWY0-1297: superpathway of purine deoxyribonucleosides degradation |
| PWY0-1298 | PWY0-1298: superpathway of pyrimidine deoxyribonucleosides degradation |
| PWY0-1319 | PWY0-1319: CDP-diacylglycerol biosynthesis II |
| PWY0-1338 | PWY0-1338: polymyxin resistance |
| PWY0-1415 | PWY0-1415: superpathway of heme biosynthesis from uroporphyrinogen-III |
| PWY0-1479 | PWY0-1479: tRNA processing |
| PWY0-1533 | PWY0-1533: methylphosphonate degradation I |
| PWY0-1586 | PWY0-1586: peptidoglycan maturation (meso-diaminopimelate containing) |
| PWY0-162 | PWY0-162: superpathway of pyrimidine ribonucleotides de novo biosynthesis |
| PWY0-166 | PWY0-166: superpathway of pyrimidine deoxyribonucleotides de novo biosynthesis (E. coli) |
| PWY0-321 | PWY0-321: phenylacetate degradation I (aerobic) |
| PWY0-41 | PWY0-41: allantoin degradation IV (anaerobic) |
| PWY0-42 | PWY0-42: 2-methylcitrate cycle I |
| PWY0-781 | PWY0-781: aspartate superpathway |
| PWY0-845 | PWY0-845: superpathway of pyridoxal 5'-phosphate biosynthesis and salvage |
| PWY0-862 | PWY0-862: (5Z)-dodec-5-enoate biosynthesis |
| PWY0-881 | PWY0-881: superpathway of fatty acid biosynthesis I (E. coli) |
| PWY1F-823 | PWY1F-823: leucopelargonidin and leucocyanidin biosynthesis |
| PWY1G-0 | PWY1G-0: mycothiol biosynthesis |
| PWY3DJ-35471 | PWY3DJ-35471: L-ascorbate biosynthesis IV |
| PWY3O-19 | PWY3O-19: ubiquinol-6 biosynthesis from 4-hydroxybenzoate (eukaryotic) |
| PWY3O-355 | PWY3O-355: stearate biosynthesis III (fungi) |
| PWY490-3 | PWY490-3: nitrate reduction VI (assimilatory) |
| PWY4FS-7 | PWY4FS-7: phosphatidylglycerol biosynthesis I (plastidic) |
| PWY4FS-8 | PWY4FS-8: phosphatidylglycerol biosynthesis II (non-plastidic) |
| PWY4LZ-257 | PWY4LZ-257: superpathway of fermentation (Chlamydomonas reinhardtii) |
| PWY66-367 | PWY66-367: ketogenesis |
| PWY66-375 | PWY66-375: leukotriene biosynthesis |
| PWY66-388 | PWY66-388: fatty acid &alpha;-oxidation III |
| PWY66-389 | PWY66-389: phytol degradation |
| PWY66-391 | PWY66-391: fatty acid &beta;-oxidation VI (peroxisome) |
| PWY66-398 | PWY66-398: TCA cycle III (animals) |
| PWY66-399 | PWY66-399: gluconeogenesis III |
| PWY66-400 | PWY66-400: glycolysis VI (metazoan) |
| PWY66-409 | PWY66-409: superpathway of purine nucleotide salvage |
| PWY66-422 | PWY66-422: D-galactose degradation V (Leloir pathway) |
| PWY6666-2 | PWY6666-2: dopamine degradation |
| PWYG-321 | PWYG-321: mycolate biosynthesis |
| PYRIDNUCSAL-PWY | PYRIDNUCSAL-PWY: NAD salvage pathway I |
| PYRIDNUCSYN-PWY | PYRIDNUCSYN-PWY: NAD biosynthesis I (from aspartate) |
| PYRIDOXSYN-PWY | PYRIDOXSYN-PWY: pyridoxal 5'-phosphate biosynthesis I |
| REDCITCYC | REDCITCYC: TCA cycle VIII (helicobacter) |
| RHAMCAT-PWY | RHAMCAT-PWY: L-rhamnose degradation I |
| RIBOSYN2-PWY | RIBOSYN2-PWY: flavin biosynthesis I (bacteria and plants) |
| RUMP-PWY | RUMP-PWY: formaldehyde oxidation I |
| SALVADEHYPOX-PWY | SALVADEHYPOX-PWY: adenosine nucleotides degradation II |
| SER-GLYSYN-PWY | SER-GLYSYN-PWY: superpathway of L-serine and glycine biosynthesis I |
| SO4ASSIM-PWY | SO4ASSIM-PWY: sulfate reduction I (assimilatory) |
| SULFATE-CYS-PWY | SULFATE-CYS-PWY: superpathway of sulfate assimilation and cysteine biosynthesis |
| TCA-GLYOX-BYPASS | TCA-GLYOX-BYPASS: superpathway of glyoxylate bypass and TCA |
| TCA | TCA: TCA cycle I (prokaryotic) |
| TEICHOICACID-PWY | TEICHOICACID-PWY: teichoic acid (poly-glycerol) biosynthesis |
| THISYN-PWY | THISYN-PWY: superpathway of thiamin diphosphate biosynthesis I |
| THISYNARA-PWY | THISYNARA-PWY: superpathway of thiamin diphosphate biosynthesis III (eukaryotes) |
| THREOCAT-PWY | THREOCAT-PWY: superpathway of L-threonine metabolism |
| THRESYN-PWY | THRESYN-PWY: superpathway of L-threonine biosynthesis |
| TRNA-CHARGING-PWY | TRNA-CHARGING-PWY: tRNA charging |
| TRPSYN-PWY | TRPSYN-PWY: L-tryptophan biosynthesis |
| UBISYN-PWY | UBISYN-PWY: superpathway of ubiquinol-8 biosynthesis (prokaryotic) |
| UDPNACETYLGALSYN-PWY | UDPNACETYLGALSYN-PWY: UDP-N-acetyl-D-glucosamine biosynthesis II |
| UDPNAGSYN-PWY | UDPNAGSYN-PWY: UDP-N-acetyl-D-glucosamine biosynthesis I |
| UNINTEGRATED | UNINTEGRATED |
| UNMAPPED | UNMAPPED |
| URDEGR-PWY | URDEGR-PWY: superpathway of allantoin degradation in plants |
| URSIN-PWY | URSIN-PWY: ureide biosynthesis |
| VALDEG-PWY | VALDEG-PWY: L-valine degradation I |
| VALSYN-PWY | VALSYN-PWY: L-valine biosynthesis |

| Table S6. LEfSe analysis of microbial pathways | | | |  |
| --- | --- | --- | --- | --- |
| Pathways | Full name | Group | LDA score log(10) | P-value |
| PWY-6167 | PWY-6167: flavin biosynthesis II (archaea) | G1_Ctrl | 2.39961964 | 0523558 |
| PWY-7286 | PWY-7286: 7-(3-amino-3-carboxypropyl)-wyosine biosynthesis | G1_Ctrl | 2.42778228 | 0079778 |
| CENTFERM-PWY | CENTFERM-PWY: pyruvate fermentation to butanoate | G1_Ctrl | 2.50373424 | 1.20E-05 |
| METHANOGENESIS-PWY | METHANOGENESIS-PWY: methanogenesis from H2 and CO2 | G1_Ctrl | 2.5396742 | 9.92E-05 |
| PWY-6590 | PWY-6590: superpathway of Clostridium acetobutylicum acidogenic fermentation | G1_Ctrl | 2.54185353 | 1.18E-05 |
| PWY-5030 | PWY-5030: L-histidine degradation III | G1_Ctrl | 2.69729301 | 0227636 |
| PWY-5198 | PWY-5198: factor 420 biosynthesis | G1_Ctrl | 2.7439508 | 8.02E-05 |
| P42-PWY | P42-PWY: incomplete reductive TCA cycle | G1_Ctrl | 2.79980925 | 0.02223199 |
| PWY-5177 | PWY-5177: glutaryl-CoA degradation | G1_Ctrl | 2.87388274 | 0089249 |
| RUMP-PWY | RUMP-PWY: formaldehyde oxidation I | G1_Ctrl | 2.92251443 | 0.01004966 |
| HSERMETANA-PWY | HSERMETANA-PWY: L-methionine biosynthesis III | G1_Ctrl | 2.99687926 | 004294 |
| PRPP-PWY | PRPP-PWY: superpathway of histidine, purine, and pyrimidine biosynthesis | G1_Ctrl | 3909396 | 0094423 |
| PWY-7456 | PWY-7456: mannan degradation | G1_Ctrl | 3.01784754 | 0112685 |
| GLYCOGENSYNTH  -PWY | GLYCOGENSYNTH-PWY: glycogen biosynthesis I (from ADP-D-Glucose) | G1_Ctrl | 3.05162223 | 0136756 |
| PWY-7383 | PWY-7383: anaerobic energy metabolism (invertebrates, cytosol) | G1_Ctrl | 3.06294833 | 4.18E-05 |
| PWY-5104 | PWY-5104: L-isoleucine biosynthesis IV | G1_Ctrl | 3.16099156 | 1.24E-07 |
| NONOXIPENT-PWY | NONOXIPENT-PWY: pentose phosphate pathway (non-oxidative branch) | G1_Ctrl | 3.17175489 | 0862465 |
| PANTO-PWY | PANTO-PWY: phosphopantothenate biosynthesis I | G1_Ctrl | 3.21694583 | 0.03107456 |
| PWY-6527 | PWY-6527: stachyose degradation | G1_Ctrl | 3.25601491 | 0021298 |
| PWY66-422 | PWY66-422: D-galactose degradation V (Leloir pathway) | G1_Ctrl | 3.28314846 | 1.05E-05 |
| PWY-6317 | PWY-6317: galactose degradation I (Leloir pathway) | G1_Ctrl | 3.31930743 | 1.79E-05 |
| PWY0-1319 | PWY0-1319: CDP-diacylglycerol biosynthesis II | G1_Ctrl | 3.38392495 | 3.37E-05 |
| PWY-5667 | PWY-5667: CDP-diacylglycerol biosynthesis I | G1_Ctrl | 3.38453573 | 3.37E-05 |
| CALVIN-PWY | CALVIN-PWY: Calvin-Benson-Bassham cycle | G1_Ctrl | 3.39848142 | 2.65E-07 |
| COA-PWY-1 | COA-PWY-1: coenzyme A biosynthesis II (mammalian) | G1_Ctrl | 3.46888185 | 4.89E-05 |
| PWY-2942 | PWY-2942: L-lysine biosynthesis III | G1_Ctrl | 3.47131355 | 0271014 |
| PWY-5097 | PWY-5097: L-lysine biosynthesis VI | G1_Ctrl | 3.5373714 | 1.37E-05 |
| PWY-7221 | PWY-7221: guanosine ribonucleotides de novo biosynthesis | G1_Ctrl | 3.54324508 | 0802459 |
| PEPTIDOGLYCANSYN-PWY | PEPTIDOGLYCANSYN-PWY: peptidoglycan biosynthesis I (meso-diaminopimelate containing) | G1_Ctrl | 3.55590217 | 3.21E-06 |
| PWY-6151 | PWY-6151: S-adenosyl-L-methionine cycle I | G1_Ctrl | 3.57725928 | 2.33E-06 |
| PWY-6386 | PWY-6386: UDP-N-acetylmuramoyl-pentapeptide biosynthesis II (lysine-containing) | G1_Ctrl | 3.60196829 | 7.77E-07 |
| PWY-6387 | PWY-6387: UDP-N-acetylmuramoyl-pentapeptide biosynthesis I (meso-diaminopimelate containing) | G1_Ctrl | 3.60244733 | 5.74E-07 |
| PWY-5686 | PWY-5686: UMP biosynthesis | G1_Ctrl | 3.61421955 | 1.04E-08 |
| NONMEVIPP-PWY | NONMEVIPP-PWY: methylerythritol phosphate pathway I | G1_Ctrl | 3.66284275 | 3.65E-08 |
| TRNA-CHARGING-PWY | TRNA-CHARGING-PWY: tRNA charging | G1_Ctrl | 3.69095772 | 4.07E-10 |
| PWY-7219 | PWY-7219: adenosine ribonucleotides de novo biosynthesis | G1_Ctrl | 3.77466325 | 6.03E-05 |
| PWY-6737 | PWY-6737: starch degradation V | G1_Ctrl | 3.83625769 | 3.06E-10 |
| P164-PWY | P164-PWY: purine nucleobases degradation I (anaerobic) | G2_AUD_nc | 2.44622846 | 0088811 |
| PWY-7209 | PWY-7209: superpathway of pyrimidine ribonucleosides degradation | G2_AUD_nc | 2.49042089 | 0.01434508 |
| PWY-6588 | PWY-6588: pyruvate fermentation to acetone | G2_AUD_nc | 2.59088458 | 1.08E-05 |
| PWY-7237 | PWY-7237: myo-, chiro- and scillo-inositol degradation | G2_AUD_nc | 2.63636127 | 0030446 |
| PWY-5304 | PWY-5304: superpathway of sulfur oxidation (Acidianus ambivalens) | G2_AUD_nc | 2.66004228 | 1.17E-09 |
| PWY-7187 | PWY-7187: pyrimidine deoxyribonucleotides de novo biosynthesis II | G2_AUD_nc | 2.81271081 | 0122867 |
| PWY-5367 | PWY-5367: petroselinate biosynthesis | G2_AUD_nc | 2.89433413 | 0742616 |
| PWY-7111 | PWY-7111: pyruvate fermentation to isobutanol (engineered) | G2_AUD_nc | 3.27407253 | 0678556 |
| VALSYN-PWY | VALSYN-PWY: L-valine biosynthesis | G2_AUD_nc | 3.3109217 | 026745 |
| ILEUSYN-PWY | ILEUSYN-PWY: L-isoleucine biosynthesis I (from threonine) | G2_AUD_nc | 3.31099796 | 026745 |
| PWY-1042 | PWY-1042: glycolysis IV (plant cytosol) | G2_AUD_nc | 3.37932527 | 013501 |
| PWY-6121 | PWY-6121: 5-aminoimidazole ribonucleotide biosynthesis I | G2_AUD_nc | 3.50399128 | 3.41E-08 |
| COMPLETE-ARO-PWY | COMPLETE-ARO-PWY: superpathway of aromatic amino acid biosynthesis | G2_AUD_nc | 3.51173324 | 3.06E-10 |
| PWY-6277 | PWY-6277: superpathway of 5-aminoimidazole ribonucleotide biosynthesis | G2_AUD_nc | 3.52835445 | 1.41E-06 |
| PWY-6122 | PWY-6122: 5-aminoimidazole ribonucleotide biosynthesis II | G2_AUD_nc | 3.52839892 | 1.41E-06 |
| PWY-6163 | PWY-6163: chorismate biosynthesis from 3-dehydroquinate | G2_AUD_nc | 3.56009004 | 1.61E-10 |
| ARO-PWY | ARO-PWY: chorismate biosynthesis I | G2_AUD_nc | 3.56118335 | 2.17E-10 |
| PWY-6595 | PWY-6595: superpathway of guanosine nucleotides degradation (plants) | G3_AUD_c | 2.36673769 | 0384424 |
| PWY-7003 | PWY-7003: glycerol degradation to butanol | G3_AUD_c | 2.56450419 | 7.43E-05 |
| CRNFORCAT-PWY | CRNFORCAT-PWY: creatinine degradation I | G3_AUD_c | 2.61361562 | 9.05E-05 |
| PWY-6549 | PWY-6549: L-glutamine biosynthesis III | G3_AUD_c | 2.72571104 | 0403465 |
| PWY66-399 | PWY66-399: gluconeogenesis III | G3_AUD_c | 2.73740883 | 0.01025285 |
| PWY-6606 | PWY-6606: guanosine nucleotides degradation II | G3_AUD_c | 2.74662798 | 8.91E-05 |
| P185-PWY | P185-PWY: formaldehyde assimilation III (dihydroxyacetone cycle) | G3_AUD_c | 2.88948086 | 0.02845267 |
| PWY-5101 | PWY-5101: L-isoleucine biosynthesis II | G3_AUD_c | 2.9203979 | 0033199 |
| PWY-7211 | PWY-7211: superpathway of pyrimidine deoxyribonucleotides de novo biosynthesis | G3_AUD_c | 2.95467927 | 0.02500532 |
| PWY-7371 | PWY-7371: 1,4-dihydroxy-6-naphthoate biosynthesis II | G3_AUD_c | 2.99374184 | 0084273 |
| ANAEROFRUCAT-PWY | ANAEROFRUCAT-PWY: homolactic fermentation | G3_AUD_c | 3.06209685 | 0.02466221 |
| CITRULBIO-PWY | CITRULBIO-PWY: L-citrulline biosynthesis | G3_AUD_c | 3.15757411 | 052275 |
| PWY-4984 | PWY-4984: urea cycle | G3_AUD_c | 3.15814437 | 0164187 |
| PWY-7196 | PWY-7196: superpathway of pyrimidine ribonucleosides salvage | G3_AUD_c | 3.19699878 | 7.25E-05 |
| PWY-7357 | PWY-7357: thiamin formation from pyrithiamine and oxythiamine (yeast) | G3_AUD_c | 3.21063696 | 047574 |
| PYRIDNUCSYN-PWY | PYRIDNUCSYN-PWY: NAD biosynthesis I (from aspartate) | G3_AUD_c | 3.23660306 | 1.67E-07 |
| PWY0-1296 | PWY0-1296: purine ribonucleosides degradation | G3_AUD_c | 3.28196523 | 0436996 |
| PWY-724 | PWY-724: superpathway of L-lysine, L-threonine and L-methionine biosynthesis II | G3_AUD_c | 3.32012292 | 6.50E-07 |
| PWY66-389 | PWY66-389: phytol degradation | G4_AH_F0_2 | 2.42126407 | 0020229 |
| PWY-5138 | PWY-5138: unsaturated, even numbered fatty acid &beta;-oxidation | G4_AH_F0_2 | 2.43600293 | 0284315 |
| KDO-NAGLIPASYN-PWY | KDO-NAGLIPASYN-PWY: superpathway of (Kdo)2-lipid A biosynthesis | G4_AH_F0_2 | 2.47548052 | 0.01695578 |
| P122-PWY | P122-PWY: heterolactic fermentation | G4_AH_F0_2 | 2.48939092 | 0.02796065 |
| AST-PWY | AST-PWY: L-arginine degradation II (AST pathway) | G4_AH_F0_2 | 2.49869613 | 0.01009036 |
| ECASYN-PWY | ECASYN-PWY: enterobacterial common antigen biosynthesis | G4_AH_F0_2 | 2.54304245 | 0.01585635 |
| PWY-6823 | PWY-6823: molybdenum cofactor biosynthesis | G4_AH_F0_2 | 2.54496845 | 0562655 |
| POLYISOPRENSYN-PWY | POLYISOPRENSYN-PWY: polyisoprenoid biosynthesis (E. coli) | G4_AH_F0_2 | 2.59747972 | 037643 |
| PWY-5384 | PWY-5384: sucrose degradation IV (sucrose phosphorylase) | G4_AH_F0_2 | 2.62465877 | 0521009 |
| REDCITCYC | REDCITCYC: TCA cycle VIII (helicobacter) | G4_AH_F0_2 | 2.6609581 | 0.02275089 |
| f-3-HYDROXYPHENYLACETATE-DEGRADATION-PWY | #N/A | G4_AH_F0_2 | 2.66815191 | 0.0245495 |
| FUC-RHAMCAT-PWY | FUC-RHAMCAT-PWY: superpathway of fucose and rhamnose degradation | G4_AH_F0_2 | 2.70491884 | 0720938 |
| PWY-5863 | PWY-5863: superpathway of phylloquinol biosynthesis | G4_AH_F0_2 | 2.72989139 | 001334 |
| PWY-5861 | PWY-5861: superpathway of demethylmenaquinol-8 biosynthesis | G4_AH_F0_2 | 2.79782339 | 0074776 |
| PWY-7269 | PWY-7269: NAD/NADP-NADH/NADPH mitochondrial interconversion (yeast) | G4_AH_F0_2 | 2.80640406 | 0587957 |
| PWY-6891 | PWY-6891: thiazole biosynthesis II (Bacillus) | G4_AH_F0_2 | 2.81603401 | 0.02893419 |
| UBISYN-PWY | UBISYN-PWY: superpathway of ubiquinol-8 biosynthesis (prokaryotic) | G4_AH_F0_2 | 2.86223498 | 0.01964081 |
| PWY-5838 | PWY-5838: superpathway of menaquinol-8 biosynthesis I | G4_AH_F0_2 | 2.88333259 | 0080242 |
| GLUCARGALACTSUPER-PWY | GLUCARGALACTSUPER-PWY: superpathway of D-glucarate and D-galactarate degradation | G4_AH_F0_2 | 2.89982693 | 0584606 |
| GALACTARDEG-PWY | GALACTARDEG-PWY: D-galactarate degradation I | G4_AH_F0_2 | 2.89985702 | 0584606 |
| PWY-5857 | PWY-5857: ubiquinol-10 biosynthesis (prokaryotic) | G4_AH_F0_2 | 2.89992037 | 0197012 |
| PWY-5856 | PWY-5856: ubiquinol-9 biosynthesis (prokaryotic) | G4_AH_F0_2 | 2.89993434 | 0197012 |
| PWY-6708 | PWY-6708: ubiquinol-8 biosynthesis (prokaryotic) | G4_AH_F0_2 | 2.89993769 | 0197012 |
| PWY-5855 | PWY-5855: ubiquinol-7 biosynthesis (prokaryotic) | G4_AH_F0_2 | 2.90000381 | 0197012 |
| PWY0-1241 | PWY0-1241: ADP-L-glycero-&beta;-D-manno-heptose biosynthesis | G4_AH_F0_2 | 2.92732453 | 0019088 |
| PWY-7254 | PWY-7254: TCA cycle VII (acetate-producers) | G4_AH_F0_2 | 2.92755766 | 0.01598437 |
| ENTBACSYN-PWY | ENTBACSYN-PWY: enterobactin biosynthesis | G4_AH_F0_2 | 2.93639286 | 0.04086736 |
| GLUCOSE1PMETAB-PWY | GLUCOSE1PMETAB-PWY: glucose and glucose-1-phosphate degradation | G4_AH_F0_2 | 2.94050784 | 0158956 |
| PWY4FS-8 | PWY4FS-8: phosphatidylglycerol biosynthesis II (non-plastidic) | G4_AH_F0_2 | 2.94105717 | 0.04212552 |
| PWY4FS-7 | PWY4FS-7: phosphatidylglycerol biosynthesis I (plastidic) | G4_AH_F0_2 | 2.94123953 | 0.04212552 |
| PWY-5723 | PWY-5723: Rubisco shunt | G4_AH_F0_2 | 2.94861046 | 0092819 |
| GLUCARDEG-PWY | GLUCARDEG-PWY: D-glucarate degradation I | G4_AH_F0_2 | 2.96297465 | 0159351 |
| FASYN-INITIAL-PWY | FASYN-INITIAL-PWY: superpathway of fatty acid biosynthesis initiation (E. coli) | G4_AH_F0_2 | 2.98475524 | 0057039 |
| GLYCOLYSIS-TCA-GLYOX-BYPASS | GLYCOLYSIS-TCA-GLYOX-BYPASS: superpathway of glycolysis, pyruvate dehydrogenase, TCA, and glyoxylate bypass | G4_AH_F0_2 | 2.98615115 | 0114361 |
| PWY-5173 | PWY-5173: superpathway of acetyl-CoA biosynthesis | G4_AH_F0_2 | 2.99744297 | 3.26E-05 |
| PWY-5083 | PWY-5083: NAD/NADH phosphorylation and dephosphorylation | G4_AH_F0_2 | 2.99919772 | 7.27E-05 |
| PWY-7013 | PWY-7013: L-1,2-propanediol degradation | G4_AH_F0_2 | 3441847 | 0266913 |
| GLYOXYLATE-BYPASS | GLYOXYLATE-BYPASS: glyoxylate cycle | G4_AH_F0_2 | 3548959 | 0035022 |
| TCA-GLYOX-BYPASS | TCA-GLYOX-BYPASS: superpathway of glyoxylate bypass and TCA | G4_AH_F0_2 | 3.01133974 | 00323 |
| PWY0-1261 | PWY0-1261: anhydromuropeptides recycling | G4_AH_F0_2 | 3.01160119 | 0.03222959 |
| NAGLIPASYN-PWY | NAGLIPASYN-PWY: lipid IVA biosynthesis | G4_AH_F0_2 | 3.01330687 | 0106676 |
| ASPASN-PWY | ASPASN-PWY: superpathway of L-aspartate and L-asparagine biosynthesis | G4_AH_F0_2 | 3.01376877 | 0.02329142 |
| GLYCOLYSIS-E-D | GLYCOLYSIS-E-D: superpathway of glycolysis and Entner-Doudoroff | G4_AH_F0_2 | 3.02596705 | 0047112 |
| PWY-7388 | PWY-7388: octanoyl-[acyl-carrier protein] biosynthesis (mitochondria, yeast) | G4_AH_F0_2 | 3.02743528 | 0135468 |
| P105-PWY | P105-PWY: TCA cycle IV (2-oxoglutarate decarboxylase) | G4_AH_F0_2 | 3.04628232 | 0873687 |
| PWY-7197 | PWY-7197: pyrimidine deoxyribonucleotide phosphorylation | G4_AH_F0_2 | 3.06850968 | 0.01834591 |
| BIOTIN-BIOSYNTHESIS-PWY | BIOTIN-BIOSYNTHESIS-PWY: biotin biosynthesis I | G4_AH_F0_2 | 3.07978681 | 0133998 |
| PWY-6519 | PWY-6519: 8-amino-7-oxononanoate biosynthesis I | G4_AH_F0_2 | 3.08398954 | 0110917 |
| PWY-6803 | PWY-6803: phosphatidylcholine acyl editing | G4_AH_F0_2 | 3.08599077 | 8.23E-05 |
| PWY-6630 | PWY-6630: superpathway of L-tyrosine biosynthesis | G4_AH_F0_2 | 3.09298443 | 0030988 |
| PWY-7184 | PWY-7184: pyrimidine deoxyribonucleotides de novo biosynthesis I | G4_AH_F0_2 | 3.10868892 | 0671451 |
| PWY-6282 | PWY-6282: palmitoleate biosynthesis I (from (5Z)-dodec-5-enoate) | G4_AH_F0_2 | 3.10873894 | 0188027 |
| PWY-4041 | PWY-4041: &gamma;-glutamyl cycle | G4_AH_F0_2 | 3.11792995 | 0060118 |
| SO4ASSIM-PWY | SO4ASSIM-PWY: sulfate reduction I (assimilatory) | G4_AH_F0_2 | 3.12588323 | 0.02592768 |
| PWY0-862 | PWY0-862: (5Z)-dodec-5-enoate biosynthesis | G4_AH_F0_2 | 3.12966337 | 0106951 |
| NAD-BIOSYNTHESIS-II | NAD-BIOSYNTHESIS-II: NAD salvage pathway II | G4_AH_F0_2 | 3.13543158 | 0.01188559 |
| PWY-7664 | PWY-7664: oleate biosynthesis IV (anaerobic) | G4_AH_F0_2 | 3.13828634 | 0129779 |
| FASYN-ELONG-PWY | FASYN-ELONG-PWY: fatty acid elongation -- saturated | G4_AH_F0_2 | 3.14704804 | 0131245 |
| PHOSLIPSYN-PWY | PHOSLIPSYN-PWY: superpathway of phospholipid biosynthesis I (bacteria) | G4_AH_F0_2 | 3.15677211 | 0166742 |
| PWY-6125 | PWY-6125: superpathway of guanosine nucleotides de novo biosynthesis II | G4_AH_F0_2 | 3.23581317 | 0096371 |
| PWY0-1297 | PWY0-1297: superpathway of purine deoxyribonucleosides degradation | G4_AH_F0_2 | 3.24741751 | 9.68E-06 |
| PWY-6628 | PWY-6628: superpathway of L-phenylalanine biosynthesis | G4_AH_F0_2 | 3.25720901 | 7.33E-05 |
| PWY-7222 | PWY-7222: guanosine deoxyribonucleotides de novo biosynthesis II | G4_AH_F0_2 | 3.2703135 | 6.98E-05 |
| PWY-7220 | PWY-7220: adenosine deoxyribonucleotides de novo biosynthesis II | G4_AH_F0_2 | 3.27046379 | 6.98E-05 |
| PWY0-1586 | PWY0-1586: peptidoglycan maturation (meso-diaminopimelate containing) | G4_AH_F0_2 | 3.33380164 | 022715 |
| PWY-5747 | PWY-5747: 2-methylcitrate cycle II | G5_AH_F3_4 | 2.20837743 | 0249954 |
| PWY0-42 | PWY0-42: 2-methylcitrate cycle I | G5_AH_F3_4 | 2.22307964 | 0351511 |
| PWY-7039 | PWY-7039: phosphatidate metabolism, as a signaling molecule | G5_AH_F3_4 | 2.30384696 | 0.01418567 |
| PWY-5920 | PWY-5920: superpathway of heme biosynthesis from glycine | G5_AH_F3_4 | 2.39782393 | 0713412 |
| PWY-5656 | PWY-5656: mannosylglycerate biosynthesis I | G5_AH_F3_4 | 2.39810586 | 0.04970525 |
| KETOGLUCONMET-PWY | KETOGLUCONMET-PWY: ketogluconate metabolism | G5_AH_F3_4 | 2.41481619 | 0.03387693 |
| PWY-922 | PWY-922: mevalonate pathway I | G5_AH_F3_4 | 2.41541055 | 0224938 |
| PWY0-1415 | PWY0-1415: superpathway of heme biosynthesis from uroporphyrinogen-III | G5_AH_F3_4 | 2.44744964 | 0.01490008 |
| METHGLYUT-PWY | METHGLYUT-PWY: superpathway of methylglyoxal degradation | G5_AH_F3_4 | 2.48370659 | 0077365 |
| GLYCOL-GLYOXDEG-PWY | GLYCOL-GLYOXDEG-PWY: superpathway of glycol metabolism and degradation | G5_AH_F3_4 | 2.49807215 | 0243071 |
| TEICHOICACID-PWY | TEICHOICACID-PWY: teichoic acid (poly-glycerol) biosynthesis | G5_AH_F3_4 | 2.63946747 | 0.04295855 |
| PWY-6285 | PWY-6285: superpathway of fatty acids biosynthesis (E. coli) | G5_AH_F3_4 | 2.64436734 | 0.02675541 |
| PWY-5918 | PWY-5918: superpathay of heme biosynthesis from glutamate | G5_AH_F3_4 | 2.65142446 | 0754771 |
| PWY-5862 | PWY-5862: superpathway of demethylmenaquinol-9 biosynthesis | G5_AH_F3_4 | 2.68358215 | 0312811 |
| PWY-5860 | PWY-5860: superpathway of demethylmenaquinol-6 biosynthesis I | G5_AH_F3_4 | 2.68374775 | 0312811 |
| HEME-BIOSYNTHESIS-II | HEME-BIOSYNTHESIS-II: heme biosynthesis I (aerobic) | G5_AH_F3_4 | 2.69135321 | 0018217 |
| PWY-7315 | PWY-7315: dTDP-N-acetylthomosamine biosynthesis | G5_AH_F3_4 | 2.69346939 | 0.01748926 |
| PWY-5121 | PWY-5121: superpathway of geranylgeranyl diphosphate biosynthesis II (via MEP) | G5_AH_F3_4 | 2.70702917 | 0.04092152 |
| PWY-5896 | PWY-5896: superpathway of menaquinol-10 biosynthesis | G5_AH_F3_4 | 2.75872094 | 0312811 |
| PWY-5845 | PWY-5845: superpathway of menaquinol-9 biosynthesis | G5_AH_F3_4 | 2.75944263 | 0312811 |
| PWY-5850 | PWY-5850: superpathway of menaquinol-6 biosynthesis I | G5_AH_F3_4 | 2.75949503 | 0312811 |
| PWY-6895 | PWY-6895: superpathway of thiamin diphosphate biosynthesis II | G5_AH_F3_4 | 2.80306412 | 0.04969619 |
| PWY-6629 | PWY-6629: superpathway of L-tryptophan biosynthesis | G5_AH_F3_4 | 2.8286642 | 0449821 |
| PWY-6471 | PWY-6471: peptidoglycan biosynthesis IV (Enterococcus faecium) | G5_AH_F3_4 | 2.85254135 | 0629241 |
| PWY-5840 | PWY-5840: superpathway of menaquinol-7 biosynthesis | G5_AH_F3_4 | 2.86183253 | 0071888 |
| PWY-5791 | PWY-5791: 1,4-dihydroxy-2-naphthoate biosynthesis II (plants) | G5_AH_F3_4 | 2.90984949 | 1.39E-07 |
| PWY-5837 | PWY-5837: 1,4-dihydroxy-2-naphthoate biosynthesis I | G5_AH_F3_4 | 2.91003202 | 1.39E-07 |
| HEXITOLDEGSUPER-PWY | HEXITOLDEGSUPER-PWY: superpathway of hexitol degradation (bacteria) | G5_AH_F3_4 | 2.9106303 | 0.01687074 |
| HEMESYN2-PWY | HEMESYN2-PWY: heme biosynthesis II (anaerobic) | G5_AH_F3_4 | 2.96029207 | 7.59E-05 |
| PWY-3781 | PWY-3781: aerobic respiration I (cytochrome c) | G5_AH_F3_4 | 2.98191912 | 7.95E-05 |
| PWY-5154 | PWY-5154: L-arginine biosynthesis III (via N-acetyl-L-citrulline) | G5_AH_F3_4 | 3.03190522 | 0168818 |
| PWY-4242 | PWY-4242: pantothenate and coenzyme A biosynthesis III | G5_AH_F3_4 | 3.07006193 | 0023077 |
| PWY-5898 | PWY-5898: superpathway of menaquinol-12 biosynthesis | G5_AH_F3_4 | 3.09321573 | 4.89E-07 |
| PWY-5899 | PWY-5899: superpathway of menaquinol-13 biosynthesis | G5_AH_F3_4 | 3.0933305 | 4.89E-07 |
| PWY-5897 | PWY-5897: superpathway of menaquinol-11 biosynthesis | G5_AH_F3_4 | 3.09338829 | 4.89E-07 |
| UDPNAGSYN-PWY | UDPNAGSYN-PWY: UDP-N-acetyl-D-glucosamine biosynthesis I | G5_AH_F3_4 | 3.15550064 | 0.02998291 |
| PWY-1269 | PWY-1269: CMP-3-deoxy-D-manno-octulosonate biosynthesis I | G5_AH_F3_4 | 3.17926491 | 0254668 |
| PWY-7560 | PWY-7560: methylerythritol phosphate pathway II | G5_AH_F3_4 | 3.19144507 | 0152296 |
| PWY-6270 | PWY-6270: isoprene biosynthesis I | G5_AH_F3_4 | 3.19417027 | 0246403 |
| PWY-6126 | PWY-6126: superpathway of adenosine nucleotides de novo biosynthesis II | G5_AH_F3_4 | 3.22667988 | 0766533 |
| PWY-7229 | PWY-7229: superpathway of adenosine nucleotides de novo biosynthesis I | G5_AH_F3_4 | 3.23349344 | 0.02178828 |
| PWY-7400 | PWY-7400: L-arginine biosynthesis IV (archaebacteria) | G5_AH_F3_4 | 3.24854952 | 0.02418284 |
| PWY-7228 | PWY-7228: superpathway of guanosine nucleotides de novo biosynthesis I | G5_AH_F3_4 | 3.25335697 | 0237676 |
| PWY66-409 | PWY66-409: superpathway of purine nucleotide salvage | G5_AH_F3_4 | 3.2564126 | 0017217 |
| ARGSYN-PWY | ARGSYN-PWY: L-arginine biosynthesis I (via L-ornithine) | G5_AH_F3_4 | 3.26417937 | 0899674 |
| GLUTORN-PWY | GLUTORN-PWY: L-ornithine biosynthesis | G5_AH_F3_4 | 3.26859222 | 0026808 |
| PWY-6124 | PWY-6124: inosine-5'-phosphate biosynthesis II | G5_AH_F3_4 | 3.27484722 | 0013016 |
| PWY-5484 | PWY-5484: glycolysis II (from fructose 6-phosphate) | G5_AH_F3_4 | 3.28511506 | 3.35E-05 |
| PWY-6123 | PWY-6123: inosine-5'-phosphate biosynthesis I | G5_AH_F3_4 | 3.3072336 | 0.01056138 |
| GLYCOLYSIS | GLYCOLYSIS: glycolysis I (from glucose 6-phosphate) | G5_AH_F3_4 | 3.30805112 | 2.46E-05 |
| PWY0-1061 | PWY0-1061: superpathway of L-alanine biosynthesis | G5_AH_F3_4 | 3.31140877 | 8.77E-07 |
| PWY66-400 | PWY66-400: glycolysis VI (metazoan) | G5_AH_F3_4 | 3.31695421 | 0021479 |
| ARGSYNBSUB-PWY | ARGSYNBSUB-PWY: L-arginine biosynthesis II (acetyl cycle) | G5_AH_F3_4 | 3.32830504 | 0080614 |

| Table S7. Association between alcohol intake and microbes and microbial pathways | | | | | | | |
| --- | --- | --- | --- | --- | --- | --- | --- |
| name | feature | coef | stderr | N | N.not.0 | pval | qval |
| Lactobacillus_saerimneri | microbe | 5.44E-06 | 2.88E-07 | 38 | 1 | 3.02E-20 | 4.19E-18 |
| Lactobacillus_johnsonii | microbe | 2.47E-05 | 1.31E-06 | 38 | 1 | 3.02E-20 | 4.19E-18 |
| Turicibacter_sanguinis | microbe | 5.29E-06 | 2.80E-07 | 38 | 1 | 3.02E-20 | 4.19E-18 |
| Bifidobacterium_pseudocatenulatum | microbe | 0017229 | 1.55E-05 | 38 | 6 | 3.27E-13 | 3.40E-11 |
| Lachnospiraceae_bacterium_2_1_58FAA | microbe | 0014005 | 1.35E-05 | 38 | 17 | 2.22E-12 | 1.85E-10 |
| Lactobacillus_crispatus | microbe | 0016224 | 1.61E-05 | 38 | 4 | 5.16E-12 | 3.58E-10 |
| Bifidobacterium_longum | microbe | 0030759 | 9.45E-05 | 38 | 33 | 024745 | 0.14705607 |
| Coprococcus_catus | microbe | 2.85E-05 | 9.36E-06 | 38 | 9 | 0427591 | 0.22234726 |
| Lactobacillus_oris | microbe | 2.11E-05 | 7.96E-06 | 38 | 8 | 0.01201504 | 0.55536203 |
| PWY0-1479: tRNA processing | microbial pathway | 6.11E-06 | 1.64E-06 | 38 | 13 | 0065234 | 0.29159627 |
| PWY-6318: L-phenylalanine degradation IV (mammalian, via side chain) | microbial pathway | 3.24E-06 | 1.11E-06 | 38 | 5 | 060655 | 0.99525117 |
| PWY0-166: superpathway of pyrimidine deoxyribonucleotides de novo biosynthesis (E. coli) | microbial pathway | 4.70E-06 | 1.84E-06 | 38 | 38 | 0.01505588 | 0.99525117 |
| URSIN-PWY: ureide biosynthesis | microbial pathway | 9.87E-07 | 3.89E-07 | 38 | 2 | 0.01568406 | 0.99525117 |
| PWY-7184: pyrimidine deoxyribonucleotides de novo biosynthesis I | microbial pathway | 4.21E-06 | 1.75E-06 | 38 | 38 | 0.02138018 | 0.99525117 |
| PWY-4242: pantothenate and coenzyme A biosynthesis III | microbial pathway | -5.27E-06 | 2.27E-06 | 38 | 38 | 0.02621522 | 0.99525117 |

**Table S8. Annotated metabolites in serum and fecal samples**

|  |  |  |  |  |  |  |  |  |  |  |
| --- | --- | --- | --- | --- | --- | --- | --- | --- | --- | --- |
| Metabolite | | | RT/retention index | m/z | InChI Key | | | Species | Platform | |
| Triptolide | | | 1.17 | 361.1614 | DFBIRQPKNDILPW-CIVMWXNOSA-N | | | [M+H]+ | LC-MS | |
| Trileptal | | | 1.30 | 253.0973 | CTRLABGOLIVAIY-UHFFFAOYSA-N | | | [M+H]+ | LC-MS | |
| Trifluoperazine | | | 2.47 | 408.1632 | ZEWQUBUPAILYHI-UHFFFAOYSA-N | | | [M+H]+ | LC-MS | |
| Torasemide | | | 1.50 | 349.1389 | NGBFQHCMQULJNZ-UHFFFAOYSA-N | | | [M+H]+ | LC-MS | |
| Threonine | | | 8.04 | 120.0641 | AYFVYJQAPQTCCC-HRFVKAFMSA-N | | | [M+H]+ | LC-MS | |
| Theophylline | | | 1.64 | 181.0706 | ZFXYFBGIUFBOJW-UHFFFAOYSA-N | | | [M+H]+ | LC-MS | |
| Sucrose | | | 8.23 | 343.1221 | CZMRCDWAGMRECN-UGDNZRGBSA-N | | | [M+H]+ | LC-MS | |
| Serotonin | | | 1.51 | 177.1009 | QZAYGJVTTNCVMB-UHFFFAOYSA-N | | | [M+H]+ | LC-MS | |
| Riboflavin | | | 5.88 | 377.1438 | AUNGANRZJHBGPY-SCRDCRAPSA-N | | | [M+H]+ | LC-MS | |
| Pipecolic acid | | | 7.53 | 130.0846 | HXEACLLIILLPRG-UHFFFAOYSA-N | | | [M+H]+ | LC-MS | |
| Pilocarpine | | | 2.52 | 209.1268 | QCHFTSOMWOSFHM-WPRPVWTQSA-N | | | [M+H]+ | LC-MS | |
| Phenylacetaldehyde | | | 5.37 | 121.0634 | DTUQWGWMVIHBKE-UHFFFAOYSA-N | | | [M+H]+ | LC-MS | |
| Parecoxib | | | 0.97 | 371.0996 | TZRHLKRLEZJVIJ-UHFFFAOYSA-N | | | [M+H]+ | LC-MS | |
| Omeprazole | | | 1.50 | 346.1208 | SUBDBMMJDZJVOS-UHFFFAOYSA-N | | | [M+H]+ | LC-MS | |
| N-Methylvaline | | | 6.86 | 132.1012 | AKCRVYNORCOYQT-UHFFFAOYSA-N | | | [M+H]+ | LC-MS | |
| N-Methylproline | | | 6.98 | 130.0851 | CWLQUGTUXBXTLF-YFKPBYRVSA-N | | | [M+H]+ | LC-MS | |
| N-Methylisoleucine | | | 6.27 | 146.1158 | KSPIYJQBLVDRRI-UHFFFAOYSA-N | | | [M+H]+ | LC-MS | |
| N-Acetylmethionine | | | 3.26 | 192.065 | XUYPXLNMDZIRQH-UHFFFAOYSA-N | | | [M+H]+ | LC-MS | |
| N-Acetylmannosamine | | | 6.98 | 222.0956 | OVRNDRQMDRJTHS-ZTVVOAFPSA-N | | | [M+H]+ | LC-MS | |
| N-Acetylglucosamine | | | 7.06 | 222.0958 | OVRNDRQMDRJTHS-RTRLPJTCSA-N | | | [M+H]+ | LC-MS | |
| N-Acetyl-D-lactosamine | | | 8.20 | 406.13 | HESSGHHCXGBPAJ-ZBELOFFLSA-N | | | [M+Na]+ | LC-MS | |
| N8-Acetylspermidine | | | 8.65 | 188.1743 | FONIWJIDLJEJTL-UHFFFAOYSA-N | | | [M+H]+ | LC-MS | |
| Midazolam | | | 1.31 | 326.0845 | DDLIGBOFAVUZHB-UHFFFAOYSA-N | | | [M+H]+ | LC-MS | |
| Methyltestosterone | | | 1.11 | 303.2299 | GCKMFJBGXUYNAG-HLXURNFRSA-N | | | [M+H]+ | LC-MS | |
| Melibiose | | | 8.90 | 360.1482 | AYRXSINWFIIFAE-GFRRCQKTSA-N | | | [M+NH4]+ | LC-MS | |
| Melatonin | | | 1.22 | 233.1299 | DRLFMBDRBRZALE-UHFFFAOYSA-N | | | [M+H]+ | LC-MS | |
| Kynurenine | | | 6.65 | 209.0902 | YGPSJZOEDVAXAB-QMMMGPOBSA-N | | | [M+H]+ | LC-MS | |
| Kynurenic acid | | | 5.98 | 190.0485 | HCZHHEIFKROPDY-UHFFFAOYSA-N | | | [M+H]+ | LC-MS | |
| Isomaltose | | | 8.96 | 365.1039 | AYRXSINWFIIFAE-OFGVQTFVSA-N | | | [M+Na]+ | LC-MS | |
| Irinotecan | | | 5 | 587.2839 | UWKQSNNFCGGAFS-XIFFEERXSA-N | | | [M+H]+ | LC-MS | |
| Irbesartan | | | 1.34 | 429.2374 | YOSHYTLCDANDAN-UHFFFAOYSA-N | | | [M+H]+ | LC-MS | |
| Indole-3-carboxaldehyde | | | 1.97 | 146.0588 | OLNJUISKUQQNIM-UHFFFAOYSA-N | | | [M+H]+ | LC-MS | |
| Indarubicin | | | 1.11 | 263.08 | CRDNMYFJWFXOCH-BUHFOSPRSA-N | | | [M+H]+ | LC-MS | |
| H-Pro-Hyp-OH | | | 8.37 | 229.1167 | ONPXCLZMBSJLSP-CSMHCCOUSA-N | | | [M+H]+ | LC-MS | |
| Homoveratrylamine | | | 4.96 | 182.1179 | ANOUKFYBOAKOIR-UHFFFAOYSA-N | | | [M+H]+ | LC-MS | |
| H-gamma-glutamyl-glutamine | | | 9.34 | 276.1175 | JBFYFLXEJFQWMU-WDSKDSINSA-N | | | [M+H]+ | LC-MS | |
| Hexamethylene?bisacetamide | | | 1.51 | 201.1586 | BNQSTAOJRULKNX-UHFFFAOYSA-N | | | [M+H]+ | LC-MS | |
| Haloperidol | | | 4.31 | 376.1564 | LNEPOXFFQSENCJ-UHFFFAOYSA-N | | | [M+H]+ | LC-MS | |
| Gly-Val | | | 7.69 | 175.1059 | STKYPAFSDFAEPH-UHFFFAOYSA-N | | | [M+H]+ | LC-MS | |
| Galacto-N-biose | | | 7.93 | 406.1305 | HMQPEDMEOBLSQB-UITYFYQISA-N | | | [M+Na]+ | LC-MS | |
| Finasteride | | | 1.32 | 373.2911 | DBEPLOCGEIEOCV-WSBQPABSSA-N | | | [M+H]+ | LC-MS | |
| D-Turanose | | | 8.23 | 360.1492 | RULSWEULPANCDV-PIXUTMIVSA-N | | | [M+NH4]+ | LC-MS | |
| Diphenoxylate | | | 1.40 | 453.2553 | HYPPXZBJBPSRLK-UHFFFAOYSA-N | | | [M+H]+ | LC-MS | |
| Cyclo(Leu-Pro) | | | 1.28 | 211.1411 | SZJNCZMRZAUNQT-IUCAKERBSA-N | | | [M+H]+ | LC-MS | |
| Cotinine | | | 1.61 | 177.1097 | UIKROCXWUNQSPJ-VIFPVBQESA-N | | | [M+H]+ | LC-MS | |
| Cellotetraose | | | 9.40 | 689.2058 | UYQJCPNSAVWAFU-FKROXMLKSA-N | | | [M+Na]+ | LC-MS | |
| Bestatin | | | 5.78 | 309.1793 | VGGGPCQERPFHOB-RDBSUJKOSA-N | | | [M+H]+ | LC-MS | |
| Alprazolam | | | 1.44 | 309.0856 | VREFGVBLTWBCJP-UHFFFAOYSA-N | | | [M+H]+ | LC-MS | |
| alpha-Keto-gamma-(methylthio)butyric acid | | | 0.98 | 149.022 | SXFSQZDSUWACKX-UHFFFAOYSA-N | | | [M+H]+ | LC-MS | |
| Ala-Val | | | 7.54 | 189.1217 | LIWMQSWFLXEGMA-WDSKDSINSA-N | | | [M+H]+ | LC-MS | |
| Ala-Ile | | | 7.50 | 203.1378 | ZSOICJZJSRWNHX-ACZMJKKPSA-N | | | [M+H]+ | LC-MS | |
| ADMA | | | 8.67 | 203.1491 | YDGMGEXADBMOMJ-LURJTMIESA-N | | | [M+H]+ | LC-MS | |
| Acetominophen | | | 1.36 | 152.0692 | RZVAJINKPMORJF-UHFFFAOYSA-N | | | [M+H]+ | LC-MS | |
| Abietic acid | | | 1.01 | 303.2308 | RSWGJHLUYNHPMX-ONCXSQPRSA-N | | | [M+H]+ | LC-MS | |
| 5-Methyl-3-deoxyuridine | | | 2.66 | 243.0957 | UYUWZFRYAAHPDN-LKEWCRSYSA-N | | | [M+H]+ | LC-MS | |
| 5-Hydroxy-3-indoleacetic acid | | | 1.98 | 192.064 | DUUGKQCEGZLZNO-UHFFFAOYSA-N | | | [M+H]+ | LC-MS | |
| 4-Guanidinobutyric acid | | | 7.42 | 146.0908 | TUHVEAJXIMEOSA-UHFFFAOYSA-N | | | [M+H]+ | LC-MS | |
| 4-Aminomethylcyclohexanecarboxylic acid | | | 6.69 | 158.1159 | GYDJEQRTZSCIOI-LJGSYFOKSA-N | | | [M+H]+ | LC-MS | |
| 4-Acetamidobutyric acid | | | 2.44 | 146.0796 | UZTFMUBKZQVKLK-UHFFFAOYSA-N | | | [M+H]+ | LC-MS | |
| 3-Hydroxyanthranilic acid | | | 1.42 | 154.0488 | WJXSWCUQABXPFS-UHFFFAOYSA-N | | | [M+H]+ | LC-MS | |
| 3.alpha.,6.alpha.-Mannotriose | | | 9.02 | 505.1747 | KJZMZIMBDAXZCX-XNRWUJQLSA-N | | | [M+H]+ | LC-MS | |
| 2'-O-Methylinosine | | | 4.40 | 283.1062 | HPHXOIULGYVAKW-IOSLPCCCSA-N | | | [M+H]+ | LC-MS | |
| 2'-O-Methylcytidine | | | 5.07 | 258.1074 | RFCQJGFZUQFYRF-ZOQUXTDFSA-N | | | [M+H]+ | LC-MS | |
| 2'-Deoxyuridine | | | 3.24 | 229.0795 | MXHRCPNRJAMMIM-SHYZEUOFSA-N | | | [M+H]+ | LC-MS | |
| 2'-Deoxyadenosine | | | 4.11 | 252.1083 | OLXZPDWKRNYJJZ-RRKCRQDMSA-N | | | [M+H]+ | LC-MS | |
| 2-alpha-Mannobiose | | | 9.31 | 343.1223 | HIWPGCMGAMJNRG-VXSGSMIHSA-N | | | [M+H]+ | LC-MS | |
| 1-Methylnicotinamide | | | 6.04 | 137.0694 | LDHMAVIPBRSVRG-UHFFFAOYSA-O | | | [M+H]+ | LC-MS | |
| Zolpidem phenyl-4-carboxylic acid | | | 3.03 | 338.1505 | FELZONDEFBLTSP-UHFFFAOYSA-N | | | [M+H]+ | LC-MS | |
| Val-Lys | | | 9.09 | 246.1798 | JKHXYJKMNSSFFL-IUCAKERBSA-N | | | [M+H]+ | LC-MS | |
| Val-His | | | 8.93 | 255.1407 | BNQVUHQWZGTIBX-IUCAKERBSA-N | | | [M+H]+ | LC-MS | |
| Val-Arg | | | 9.01 | 274.1861 | IBIDRSSEHFLGSD-YUMQZZPRSA-N | | | [M+H]+ | LC-MS | |
| Ursocholic acid | | | 1.56 | 391.2821 | BHQCQFFYRZLCQQ-QPKJLDHASA-N | | | [M+H-H2O]+ | LC-MS | |
| Urobilin | | | 5.19 | 591.3156 | KDCCOOGTVSRCHX-UYMYUHGCSA-N | | | [M+H]+ | LC-MS | |
| Tyr-Val | | | 7.04 | 281.1479 | OYOQKMOWUDVWCR-RYUDHWBXSA-N | | | [M+H]+ | LC-MS | |
| Trp-Val | | | 6.22 | 304.165 | LWFWZRANSFAJDR-JSGCOSHPSA-N | | | [M+H]+ | LC-MS | |
| Trp-Leu | | | 5.79 | 318.1792 | LYMVXFSTACVOLP-ZFWWWQNUSA-N | | | [M+H]+ | LC-MS | |
| Tris(2-butoxyethyl) phosphate | | | 1 | 399.2482 | WTLBZVNBAKMVDP-UHFFFAOYSA-N | | | [M+H]+ | LC-MS | |
| Trimethoprim | | | 4.32 | 291.1467 | IEDVJHCEMCRBQM-UHFFFAOYSA-N | | | [M+H]+ | LC-MS | |
| Trihydroxycholestanoic acid | | | 1.51 | 468.3652 | CNWPIIOQKZNXBB-VCVMUKOKSA-N | | | [M+NH4]+ | LC-MS | |
| Trigonelline | | | 7.23 | 138.0539 | WWNNZCOKKKDOPX-UHFFFAOYSA-N | | | [M+H]+ | LC-MS | |
| Triethanolamine | | | 6.43 | 150.1108 | GSEJCLTVZPLZKY-UHFFFAOYSA-N | | | [M+H]+ | LC-MS | |
| trans-Zeatin | | | 5.63 | 220.1192 | UZKQTCBAMSWPJD-FARCUNLSSA-N | | | [M+H]+ | LC-MS | |
| trans-Nicotine-1'-oxide | | | 6.09 | 179.1164 | RWFBQHICRCUQJJ-TVKKRMFBSA-N | | | [M+H]+ | LC-MS | |
| trans-3'-Hydroxycotinine | | | 2.05 | 193.0959 | XOKCJXZZNAUIQN-DTWKUNHWSA-N | | | [M+H]+ | LC-MS | |
| trans-13-Octadecenoic acid | | | 1.10 | 283.263 | BDLLSHRIFPDGQB-AATRIKPKSA-N | | | [M+H]+ | LC-MS | |
| Thionin | | | 12.95 | 137.0442 | SHHZUHVSERGLNW-UHFFFAOYSA-O | | | [M+H]+ | LC-MS | |
| Thiamine monophosphate | | | 6.62 | 122.0705 | HZSAJDVWZRBGIF-UHFFFAOYSA-N | | | [M+H-C6H10SO4NP]+ | LC-MS | |
| Thiamine | | | 6.63 | 265.1113 | JZRWCGZRTZMZEH-UHFFFAOYSA-N | | | [Cat]+ | LC-MS | |
| Tauroursodeoxycholic acid | | | 5.17 | 500.3027 | BHTRKEVKTKCXOH-OGTVOWCVSA-N | | | [M+H]+ | LC-MS | |
| Stercobilin | | | 5.17 | 595.3473 | TYOWQSLRVAUSMI-UHFFFAOYSA-N | | | [M+H]+ | LC-MS | |
| Stachydrine | | | 6.87 | 144.1014 | CMUNUTVVOOHQPW-UHFFFAOYSA-N | | | [M+H]+ | LC-MS | |
| Sinapine | | | 2.83 | 310.164 | HUJXHFRXWWGYQH-UHFFFAOYSA-O | | | [M]+ | LC-MS | |
| S-Allyl-L-Cysteine | | | 6.83 | 162.0575 | ZFAHNWWNDFHPOH-UHFFFAOYSA-N | | | [M+H]+ | LC-MS | |
| Quinine | | | 4.35 | 325.1893 | LOUPRKONTZGTKE-WZBLMQSHSA-N | | | [M+H]+ | LC-MS | |
| Quetiapine | | | 1.67 | 384.1717 | URKOMYMAXPYINW-UHFFFAOYSA-N | | | [M+H]+ | LC-MS | |
| PyroGlu-Gly-Arg | | | 8.55 | 343.1617 | IWWPPYAHLDRYPH-UHFFFAOYSA-N | | | [M+H]+ | LC-MS | |
| Pyridoxamine | | | 7.78 | 169.0958 | NHZMQXZHNVQTQA-UHFFFAOYSA-N | | | [M+H]+ | LC-MS | |
| Pyridoxal | | | 2.45 | 168.0644 | RADKZDMFGJYCBB-UHFFFAOYSA-N | | | [M+H]+ | LC-MS | |
| Propranolol | | | 3.31 | 260.1634 | AQHHHDLHHXJYJD-UHFFFAOYSA-N | | | [M+H]+ | LC-MS | |
| Phe-Trp | | | 5.62 | 352.1634 | JMCOUWKXLXDERB-WMZOPIPTSA-N | | | [M+H]+ | LC-MS | |
| Phe-Phe | | | 5.57 | 313.1534 | GKZIWHRNKRBEOH-HOTGVXAUSA-N | | | [M+H]+ | LC-MS | |
| Phencyclidine | | | 1.38 | 244.1906 | JTJMJGYZQZDUJJ-UHFFFAOYSA-N | | | [M+H]+ | LC-MS | |
| Phe-Arg | | | 8.49 | 322.186 | OZILORBBPKKGRI-RYUDHWBXSA-N | | | [M+H]+ | LC-MS | |
| Perindopril | | | 1.50 | 369.2257 | IPVQLZZIHOAWMC-LWQJKRBBSA-N | | | [M+H]+ | LC-MS | |
| Palmitoylcarnitine | | | 2.78 | 400.3407 | XOMRRQXKHMYMOC-UHFFFAOYSA-N | | | [M+H]+ | LC-MS | |
| Omeprazole sulfone N-oxide | | | 1.47 | 378.1101 | ZBGMHRIYIGAEGJ-UHFFFAOYSA-N | | | [M+H]+ | LC-MS | |
| Omeprazole sulfone | | | 1.18 | 362.1159 | IXEQEYRTSRFZEO-UHFFFAOYSA-N | | | [M+H]+ | LC-MS | |
| Oleoyl-L-carnitine | | | 2.69 | 426.3556 | IPOLTUVFXFHAHI-WHIOSMTNSA-N | | | [M+H]+ | LC-MS | |
| Ofloxacin | | | 4.36 | 362.15 | GSDSWSVVBLHKDQ-UHFFFAOYSA-N | | | [M+H]+ | LC-MS | |
| Octanoylcarnitine | | | 4.38 | 288.2154 | CXTATJFJDMJMIY-CYBMUJFWSA-N | | | [M+H]+ | LC-MS | |
| Nudifloramide | | | 2.13 | 153.0648 | JLQSXXWTCJPCBC-UHFFFAOYSA-N | | | [M+H]+ | LC-MS | |
| N-Methyltyramine | | | 4.78 | 152.1054 | AXVZFRBSCNEKPQ-UHFFFAOYSA-N | | | [M+H]+ | LC-MS | |
| Neolinustatin | | | 6.67 | 446.1624 | WOSYVGNDRYBQCQ-ANAWWYLGSA-N | | | [M+Na]+ | LC-MS | |
| N-Desmethylcyclobenzaprine | | | 2.63 | 262.1625 | XECQQDXTQRYYBH-UHFFFAOYSA-N | | | [M+H]+ | LC-MS | |
| N-Carboxyethyl-.gamma.-aminobutyric acid | | | 7.89 | 176.0906 | SRGQUICKDUQCKO-UHFFFAOYSA-N | | | [M+H]+ | LC-MS | |
| N-Butylscopolaminium | | | 2.36 | 360.2171 | YBCNXCRZPWQOBR-SFYZFMCISA-N | | | [Cat]+ | LC-MS | |
| Nadolol | | | 5.40 | 310.2015 | VWPOSFSPZNDTMJ-UCWKZMIHSA-N | | | [M+H]+ | LC-MS | |
| N-Acetyltryptophan | | | 2.76 | 247.1077 | DZTHIGRZJZPRDV-GFCCVEGCSA-N | | | [M+H]+ | LC-MS | |
| N-Acetyl-L-carnosine | | | 8.15 | 269.1229 | BKAYIFDRRZZKNF-VIFPVBQESA-N | | | [M+H]+ | LC-MS | |
| N-2-Hydroxycyclopentyladenosine | | | 6.62 | 352.1604 | GYWXTRVEUURNEW-QPIWWKSXSA-N | | | [M+H]+ | LC-MS | |
| N.epsilon.-Methyl-L-lysine | | | 9.08 | 161.1269 | PQNASZJZHFPQLE-UHFFFAOYSA-N | | | [M+H]+ | LC-MS | |
| N.alpha.-Methyl-L-lysine | | | 6.33 | 144.101 | OLYPWXRMOFUVGH-LURJTMIESA-N | | | [M+H-NH3]+ | LC-MS | |
| N.alpha.-Acetyl-L-lysine | | | 7.99 | 189.1223 | VEYYWZRYIYDQJM-ZETCQYMHSA-N | | | [M+H]+ | LC-MS | |
| N-.alpha.-Acetyl-L-arginine | | | 7.86 | 217.1279 | SNEIUMQYRCDYCH-LURJTMIESA-N | | | [M+H]+ | LC-MS | |
| N,N-Dimethylaniline | | | 3.14 | 122.0956 | JLTDJTHDQAWBAV-UHFFFAOYSA-N | | | [M+H]+ | LC-MS | |
| N,N,N-trimethyllysine | | | 9.01 | 189.1585 | MXNRLFUSFKVQSK-UHFFFAOYSA-N | | | [M+H]+ | LC-MS | |
| N,N,N-trimethyl-5-({[(3s,5s,7s)-tricyclo[3.3.1.1~3,7~]decan-1-yl]methyl}amino)pentan-1-aminium | | | 8.28 | 145.1689 | IWYZMMIPAJDJBN-UHFFFAOYSA-N | | | [Cat-C11H16]+ | LC-MS | |
| Metformin | | | 5.40 | 130.1073 | XZWYZXLIPXDOLR-UHFFFAOYSA-N | | | [M+H]+ | LC-MS | |
| Lys-Val | | | 8.93 | 246.1805 | YQAIUOWPSUOINN-IUCAKERBSA-N | | | [M+H]+ | LC-MS | |
| Lys-Ile | | | 8.60 | 260.1955 | FMIIKPHLJKUXGE-GUBZILKMSA-N | | | [M+H]+ | LC-MS | |
| Lys-Ala | | | 9.38 | 218.1482 | QOOWRKBDDXQRHC-BQBZGAKWSA-N | | | [M+H]+ | LC-MS | |
| L-Saccharopine | | | 9.04 | 259.1289 | ZDGJAHTZVHVLOT-UHFFFAOYSA-N | | | [M+H-H2O]+ | LC-MS | |
| Losartancarboxaldehyde | | | 1.40 | 207.0949 | FQZSMTSTFMNWQF-UHFFFAOYSA-N | | | [M+H-C8H11ClN4O]+ | LC-MS | |
| Lithocholylglycine | | | 4.93 | 416.3138 | XBSQTYHEGZTYJE-OETIFKLTSA-N | | | [M+H-H2O]+ | LC-MS | |
| Linustatin | | | 7.05 | 432.1476 | FERSMFQBWVBKQK-RWXSRRFGSA-N | | | [M+Na]+ | LC-MS | |
| Linoleoylcarnitine | | | 2.89 | 424.3394 | MJLXQSQYKZWZCB-DQFWFXSYSA-N | | | [M+H]+ | LC-MS | |
| Levocetirizine | | | 2.81 | 389.1614 | ZKLPARSLTMPFCP-OAQYLSRUSA-N | | | [M+H]+ | LC-MS | |
| L-Cystine | | | 9.69 | 241.0295 | LEVWYRKDKASIDU-IMJSIDKUSA-N | | | [M+H]+ | LC-MS | |
| Lamotrigine | | | 1.84 | 256.015 | PYZRQGJRPPTADH-UHFFFAOYSA-N | | | [M+H]+ | LC-MS | |
| Iohexol | | | 7.25 | 821.8857 | NTHXOOBQLCIOLC-UHFFFAOYSA-N | | | [M+H]+ | LC-MS | |
| Ile-Trp | | | 5.99 | 318.1793 | BVRPESWOSNFUCJ-NZVBXONLSA-N | | | [M+H]+ | LC-MS | |
| Ile-Arg | | | 8.69 | 288.2013 | HYXQKVOADYPQEA-CIUDSAMLSA-N | | | [M+H]+ | LC-MS | |
| Homoarginine | | | 9.13 | 189.133 | QUOGESRFPZDMMT-YFKPBYRVSA-N | | | [M+H]+ | LC-MS | |
| His-Val | | | 8.46 | 255.144 | VLDVBZICYBVQHB-IUCAKERBSA-N | | | [M+H]+ | LC-MS | |
| His-Tyr | | | 8.50 | 319.1392 | HTOOKGDPMXSJSY-STQMWFEESA-N | | | [M+H]+ | LC-MS | |
| Histamine | | | 8.07 | 112.0857 | NTYJJOPFIAHURM-UHFFFAOYSA-N | | | [M+H]+ | LC-MS | |
| His-Pro | | | 5.85 | 235.1178 | LNCFUHAPNTYMJB-IUCAKERBSA-N | | | [M+H-H2O]+ | LC-MS | |
| His-Leu | | | 7.92 | 269.1593 | MMFKFJORZBJVNF-UWVGGRQHSA-N | | | [M+H]+ | LC-MS | |
| His-Ile | | | 8.05 | 269.1596 | IDXZDKMBEXLFMB-HGNGGELXSA-N | | | [M+H]+ | LC-MS | |
| His-Ala | | | 9.11 | 227.1125 | FRJIAZKQGSCKPQ-FSPLSTOPSA-N | | | [M+H]+ | LC-MS | |
| Hexanoyl-L-carnitine | | | 4.80 | 260.1842 | VVPRQWTYSNDTEA-LLVKDONJSA-N | | | [M+H]+ | LC-MS | |
| Hesperidin | | | 5.31 | 611.1938 | QUQPHWDTPGMPEX-UHFFFAOYSA-N | | | [M+H]+ | LC-MS | |
| Glycoursodeoxycholic acid | | | 4.52 | 432.3087 | GHCZAUBVMUEKKP-XROMFQGDSA-N | | | [M+H-H2O]+ | LC-MS | |
| Glycerophosphocholine | | | 8.38 | 280.0914_258.1093 | [M+H]+_[M+Na]+ | LC-MS |  |  |  |  |
| Glu-Val-Lys | | | 9.18 | 375.2217 | ZYRXTRTUCAVNBQ-UHFFFAOYSA-N | | | [M+H]+ | LC-MS | |
| Glu-Leu | | | 7.62 | 261.1429 | YBAFDPFAUTYYRW-YUMQZZPRSA-N | | | [M+H]+ | LC-MS | |
| Glu-Ile-Lys | | | 8.94 | 389.2373 | XTZDZAXYPDISRR-UHFFFAOYSA-N | | | [M+H]+ | LC-MS | |
| Gln-Val | | | 7.84 | 246.1434 | MRVYVEQPNDSWLH-XPUUQOCRSA-N | | | [M+H]+ | LC-MS | |
| Gln-Ala | | | 8.53 | 218.1122 | FAQVCWVVIYYWRR-WHFBIAKZSA-N | | | [M+H]+ | LC-MS | |
| Gabapentin | | | 6 | 172.1315 | UGJMXCAKCUNAIE-UHFFFAOYSA-N | | | [M+H]+ | LC-MS | |
| Eutylone | | | 3.64 | 188.1059 | YERSNXHEOIYEGX-UHFFFAOYSA-N | | | [M+H-CH4O2]+ | LC-MS | |
| Enalapril | | | 1.51 | 377.2016 | GBXSMTUPTTWBMN-XIRDDKMYSA-N | | | [M+H]+ | LC-MS | |
| ectoine | | | 7.60 | 143.08 | WQXNXVUDBPYKBA-YFKPBYRVSA-N | | | [M+H]+ | LC-MS | |
| DL-.alpha.-Aminopimelic acid | | | 8.16 | 176.0905 | JUQLUIFNNFIIKC-UHFFFAOYSA-N | | | [M+H]+ | LC-MS | |
| Creatine | | | 7.85 | 132.0758 | CVSVTCORWBXHQV-UHFFFAOYSA-N | | | [M+H]+ | LC-MS | |
| Chenodeoxycholic acid 24-acyl-.beta.-D-glucuronide | | | 6.34 | 591.3118 | ZTJBLIAPAIPNJE-BWGRGVIUSA-N | | | [M+Na]+ | LC-MS | |
| Ceftazidime | | | 7.75 | 547.1039 | ORFOPKXBNMVMKC-DWVKKRMSSA-N | | | [M+H]+ | LC-MS | |
| Canrenoic acid | | | 1.43 | 341.2072 | PBKZPPIHUVSDNM-WNHSNXHDSA-N | | | [M+H-H2O]+ | LC-MS | |
| Biliverdin | | | 1.51 | 583.2531 | QBUVFDKTZJNUPP-BBROENKCSA-N | | | [M+H]+ | LC-MS | |
| Betonicine | | | 7.46 | 160.0956 | MUNWAHDYFVYIKH-LWOQYNTDSA-N | | | [M+H]+ | LC-MS | |
| Benzyldimethyltetradecylammonium | | | 1.52 | 332.3297 | WNBGYVXHFTYOBY-UHFFFAOYSA-N | | | [Cat]+ | LC-MS | |
| Arg-Val | | | 8.89 | 274.1864 | DAQIJMOLTMGJLO-YUMQZZPRSA-N | | | [M+H]+ | LC-MS | |
| Arg-Tyr | | | 8.98 | 338.1802 | XTWSWDJMIKUJDQ-RYUDHWBXSA-N | | | [M+H]+ | LC-MS | |
| Arg-Phe | | | 8.41 | 322.1851 | PQBHGSGQZSOLIR-RYUDHWBXSA-N | | | [M+H]+ | LC-MS | |
| Arg-Leu | | | 8.43 | 288.2018 | WYBVBIHNJWOLCJ-DTWKUNHWSA-N | | | [M+H]+ | LC-MS | |
| Arg-Ala | | | 9.35 | 246.1552 | WVRUNFYJIHNFKD-WDSKDSINSA-N | | | [M+H]+ | LC-MS | |
| Androsterone | | | 4.26 | 291.2356 | QGXBDMJGAMFCBF-HLUDHZFRSA-N | | | [M+H]+ | LC-MS | |
| Ala-Gln | | | 8.82 | 218.1116 | HJCMDXDYPOUFDY-WHFBIAKZSA-N | | | [M+H]+ | LC-MS | |
| Acetaminophen sulfate | | | 1.48 | 232.026 | IGTYILLPRJOVFY-UHFFFAOYSA-N | | | [M+H]+ | LC-MS | |
| 5-Cholesten-3-beta-7-alpha-diol | | | 2.64 | 403.3591 | OYXZMSRRJOYLLO-RVOWOUOISA-N | | | [M+H]+ | LC-MS | |
| 5-Amino-2-methoxyphenol | | | 5.70 | 140.0695 | BLQFHJKRTDIZLX-UHFFFAOYSA-N | | | [M+H]+ | LC-MS | |
| 4-Hydroxyvalsartan | | | 1.40 | 352.1751 | ICSQZMPILLPFKC-XLDIYJRPSA-N | | | [M+H-C5H8O2]+ | LC-MS | |
| 4-Hydroxymandelonitrile | | | 2.67 | 150.0531 | HOOOPXDSCKBLFG-UHFFFAOYSA-N | | | [M+H]+ | LC-MS | |
| 4-Cholestenone | | | 0.99 | 385.3434 | NYOXRYYXRWJDKP-GYKMGIIDSA-N | | | [M+H]+ | LC-MS | |
| 4,4-Dimethyl-L-glutamic acid | | | 5.17 | 158.0793 | HDVPVAJBJJYYBO-BYPYZUCNSA-N | | | [M+H-H2O]+ | LC-MS | |
| 3-Methylglutarylcarnitine | | | 5.40 | 290.1574 | HFCPFJNSBPQJDP-UHFFFAOYSA-N | | | [M+H]+ | LC-MS | |
| 3-Hydroxyoleylcarnitine | | | 4.45 | 442.3503 | YBCVTTMMURGSEY-LMLDZDDJSA-N | | | [M+H]+ | LC-MS | |
| 3-Hydroxybutyrylcarnitine | | | 7.35 | 248.1476 | UEFRDQSMQXDWTO-YGPZHTELSA-N | | | [M+H]+ | LC-MS | |
| 3-(2-Hydroxyethyl)indole | | | 4.77 | 144.0795 | MBBOMCVGYCRMEA-UHFFFAOYSA-N | | | [M+H-H2O]+ | LC-MS | |
| 2S-Amino-4E-octadecene-1,3S-diol | | | 4.40 | 300.2884 | WWUZIQQURGPMPG-DNWQSSKHSA-N | | | [M+H]+ | LC-MS | |
| 2-Phenylethanol | | | 4.56 | 105.0688 | WRMNZCZEMHIOCP-UHFFFAOYSA-N | | | [M+H-H2O]+ | LC-MS | |
| 2-Methylbutyryl-L-carnitine | | | 5.12 | 246.1687 | IHCPDBBYTYJYIL-QVDQXJPCSA-N | | | [M+H]+ | LC-MS | |
| 2-Imino-4-methylpiperidine | | | 4.64 | 113.1059 | GGDLOMFAKKVDPT-UHFFFAOYSA-N | | | [M+H]+ | LC-MS | |
| 2-Despiperidyl-2-(5-carboxypentylamine)repaglinide | | | 5.54 | 194.1165 | ZOMBGPVQRXZSGW-UHFFFAOYSA-N | | | [M+H-C16H21NO4]+ | LC-MS | |
| 2'-Deoxyinosine | | | 5.15 | 253.0913 | VGONTNSXDCQUGY-JXBXZBNISA-N | | | [M+H]+ | LC-MS | |
| 2-Acetylpyrazine | | | 7.44 | 123.0543 | DBZAKQWXICEWNW-UHFFFAOYSA-N | | | [M+H]+ | LC-MS | |
| 2-Acetamido-2-deoxy-3-O-(.beta.-D-galactopyranosyl)-D-glucopyranose | | | 7.93 | 384.1481 | HMQPEDMEOBLSQB-RPHKZZMBSA-N | | | [M+H]+ | LC-MS | |
| 1-Methylhistamine | | | 7.14 | 126.101 | FHQDWPCFSJMNCT-UHFFFAOYSA-N | | | [M+H]+ | LC-MS | |
| 1-Acetyl-3-piperidinamine | | | 7.31 | 143.1164 | IXINAEREEWKDJW-UHFFFAOYSA-N | | | [M+H]+ | LC-MS | |
| 12-Ketodeoxycholic acid | | | 1.35 | 391.2843 | CVNYHSDFZXHMMJ-VPUMZWJWSA-N | | | [M+H]+ | LC-MS | |
| 1,5-Pentanediamine | | | 8.80 | 103.122 | VHRGRCVQAFMJIZ-UHFFFAOYSA-N | | | [M+H]+ | LC-MS | |
| .beta.-Hyodeoxycholic acid | | | 1.49 | 375.2869 | DGABKXLVXPYZII-MMTMODRTSA-N | | | [M+H-H2O]+ | LC-MS | |
| .alpha.,.alpha.'-Dilaurin | | | 0.97 | 439.3769 | KUVAEMGNHJQSMH-UHFFFAOYSA-N | | | [M+H-H2O]+ | LC-MS | |
| (S)-Desmethylcitalopram | | | 2.69 | 311.153 | PTJADDMMFYXMMG-IBGZPJMESA-N | | | [M+H]+ | LC-MS | |
| (S)-1-Methylnicotinium | | | 5.73 | 177.1354 | HIOROZIUERMMRQ-LLVKDONJSA-N | | | [Cat]+ | LC-MS | |
| (R)-Butyrylcarnitine | | | 5.51 | 232.1533 | QWYFHHGCZUCMBN-SECBINFHSA-N | | | [M+H]+ | LC-MS | |
| (3-Carboxypropyl)trimethylammonium | | | 7.54 | 146.1166 | JHPNVNIEXXLNTR-UHFFFAOYSA-O | | | [Cat]+ | LC-MS | |
| (2R)-3-Hydroxyisovaleroylcarnitine | | | 6.98 | 262.1637 | IGLHHSKNBDXCEY-SECBINFHSA-N | | | [M+H]+ | LC-MS | |
| Valsartan | | | 1.86 | 436.234 | ACWBQPMHZXGDFX-QFIPXVFZSA-N | | | [M+H]+ | LC-MS | |
| Tryptamine | | | 4.77 | 161.1055 | APJYDQYYACXCRM-UHFFFAOYSA-N | | | [M+H]+ | LC-MS | |
| Trimethylamine N-oxide | | | 5.39 | 76.0749 | UYPYRKYUKCHHIB-UHFFFAOYSA-N | | | [M+H]+ | LC-MS | |
| Taurocholic acid | | | 5.94 | 533.3234_516.2976 | WBWWGRHZICKQGZ-HZAMXZRMSA-N | | | [M+H]+_[M+NH4]+ | LC-MS | |
| Synephrine | | | 5.48 | 168.1006 | YRCWQPVGYLYSOX-UHFFFAOYSA-N | | | [M+H]+ | LC-MS | |
| Rifaximin | | | 1.26 | 786.3597 | NZCRJKRKKOLAOJ-IIBMFGDOSA-N | | | [M+H]+ | LC-MS | |
| Propionylcarnitine | | | 6.15 | 218.138 | UFAHZIUFPNSHSL-UHFFFAOYSA-N | | | [M+H]+ | LC-MS | |
| Piperine | | | 1.07 | 286.1427 | MXXWOMGUGJBKIW-YPCIICBESA-N | | | [M+H]+ | LC-MS | |
| Phenylethanolamine | | | 5.37 | 138.0908 | ULSIYEODSMZIPX-UHFFFAOYSA-N | | | [M+H]+ | LC-MS | |
| Phenylalanine | | | 6.63 | 166.0854_149.058 | COLNVLDHVKWLRT-UHFFFAOYSA-N | | | [M+H]+_[M+H-NH3]+ | LC-MS | |
| Phenylacetylglutamine | | | 5.72 | 265.1174_287.0987 | JFLIEFSWGNOPJJ-JTQLQIEISA-N | | | [M+H]+_[M+Na]+ | LC-MS | |
| Pantoprazole | | | 1.31 | 384.0807 | IQPSEEYGBUAQFF-UHFFFAOYSA-N | | | [M+H]+ | LC-MS | |
| Norleucine | | | 6.78 | 132.1006 | LRQKBLKVPFOOQJ-YFKPBYRVSA-N | | | [M+H]+ | LC-MS | |
| N-omega-Acetylhistamine | | | 5.86 | 154.0942 | XJWPISBUKWZALE-UHFFFAOYSA-N | | | [M+H]+ | LC-MS | |
| N-Methylhistidine | | | 9.03 | 170.091 | BRMWTNUJHUMWMS-LURJTMIESA-N | | | [M+H]+ | LC-MS | |
| N-Acetylhistidine | | | 7.89 | 198.085 | KBOJOGQFRVVWBH-ZETCQYMHSA-N | | | [M+H]+ | LC-MS | |
| N2,N2-Dimethylguanosine | | | 5.40 | 312.1288 | RSPURTUNRHNVGF-IOSLPCCCSA-N | | | [M+H]+ | LC-MS | |
| N,N-Dimethylarginine | | | 8.73 | 203.1489 | NWGZOALPWZDXNG-UHFFFAOYSA-N | | | [M+H]+ | LC-MS | |
| Metronidazole | | | 1.26 | 172.0718 | VAOCPAMSLUNLGC-UHFFFAOYSA-N | | | [M+H]+ | LC-MS | |
| Linezolide | | | 1.26 | 338.157 | TYZROVQLWOKYKF-ZDUSSCGKSA-N | | | [M+H]+ | LC-MS | |
| Glutamine | | | 8.37 | 147.0752_130.0489 | ZDXPYRJPNDTMRX-UHFFFAOYSA-N | | | [M+H]+_[M+H-NH3]+ | LC-MS | |
| gamma-Glutamylleucine | | | 7.77 | 261.1433 | MYFMARDICOWMQP-YUMQZZPRSA-N | | | [M+H]+ | LC-MS | |
| Esomeprazole | | | 1.45 | 346.1216 | SUBDBMMJDZJVOS-DEOSSOPVSA-N | | | [M+H]+ | LC-MS | |
| Diazepam | | | 1.07 | 285.0775 | AAOVKJBEBIDNHE-UHFFFAOYSA-N | | | [M+H]+ | LC-MS | |
| Dehydrocholic acid | | | 1.40 | 403.247 | OHXPGWPVLFPUSM-KLRNGDHRSA-N | | | [M+H]+ | LC-MS | |
| Cytidine | | | 6.71 | 244.0915 | UHDGCWIWMRVCDJ-XVFCMESISA-N | | | [M+H]+ | LC-MS | |
| Carnitine | | | 7.55 | 162.1118 | PHIQHXFUZVPYII-ZCFIWIBFSA-N | | | [M+H]+ | LC-MS | |
| Carbamazepine | | | 1.22 | 237.1052 | FFGPTBGBLSHEPO-UHFFFAOYSA-N | | | [M+H]+ | LC-MS | |
| Caffeine | | | 1.26 | 195.087 | RYYVLZVUVIJVGH-UHFFFAOYSA-N | | | [M+H]+ | LC-MS | |
| Biotin | | | 2.96 | 245.0955 | YBJHBAHKTGYVGT-ZKWXMUAHSA-N | | | [M+H]+ | LC-MS | |
| Betaine | | | 7 | 118.0861 | KWIUHFFTVRNATP-UHFFFAOYSA-N | | | [M+H]+ | LC-MS | |
| Arginine | | | 9.23 | 175.1185 | ODKSFYDXXFIFQN-BYPYZUCNSA-N | | | [M+H]+ | LC-MS | |
| Amiloride | | | 5.37 | 230.0541 | XSDQTOBWRPYKKA-UHFFFAOYSA-N | | | [M+H]+ | LC-MS | |
| alpha-Methylhistidine | | | 8.87 | 170.0914 | HRRYYCWYCMJNGA-UHFFFAOYSA-N | | | [M+H]+ | LC-MS | |
| Ala-Ala | | | 8.11 | 161.0907 | DEFJQIDDEAULHB-QWWZWVQMSA-N | | | [M+H]+ | LC-MS | |
| Acetylcarnitine | | | 6.96 | 204.1228 | [M+H]+ | | | | LC-MS |  |
| 3-Methyl-histidine | | | 9.19 | 170.0914 | JDHILDINMRGULE-LURJTMIESA-N | | | [M+H]+ | LC-MS | |
| 2'-Deoxycytidine | | | 5.83 | 228.0962 | CKTSBUTUHBMZGZ-SHYZEUOFSA-N | | | [M+H]+ | LC-MS | |
| 1-Methylguanosine | | | 5.62 | 298.1133 | UTAIYTHAJQNQDW-KQYNXXCUSA-N | | | [M+H]+ | LC-MS | |
| 1-Methyladenosine | | | 7.17 | 282.1184 | GFYLSDSUCHVORB-IOSLPCCCSA-N | | | [M+H]+ | LC-MS | |
| 1,7-Dimethyluric acid | | | 4.30 | 197.0654 | NOFNCLGCUJJPKU-UHFFFAOYSA-N | | | [M+H]+ | LC-MS | |
| stearic acid | | | 787622 | 117 | QIQXTHQIDYTFRH-UHFFFAOYSA-N | | | | GC-MS | |
| ornithine | | | 527113 | 142 | AHLPHDHHMVZTML-BYPYZUCNSA-N | | | | GC-MS | |
| oleic acid | | | 779120 | 339 | ZQPPMHVWECSIRJ-KTKRTIGZSA-N | | | | GC-MS | |
| heptadecanoic acid | | | 751309 | 117 | KEMQGTRYUADPNZ-UHFFFAOYSA-N | | | | GC-MS | |
| capric acid | | | 452386 | 229 | GHVNFZFCNZKVNT-UHFFFAOYSA-N | | | | GC-MS | |
| pelargonic acid | | | 399229 | 117 | FBUKVWPVBMHYJY-UHFFFAOYSA-N | | | | GC-MS | |
| isothreonic acid | | | 489385 | 292 | JPIJQSOTBSSVTP-GBXIJSLDSA-N | | | | GC-MS | |
| glycolic acid | | | 227215 | 177 | AEMRFAOFKBGASW-UHFFFAOYSA-N | | | | GC-MS | |
| cholesterol | | | 1078536 | 129 | HVYWMOMLDIMFJA-DPAQBDIFSA-N | | | | GC-MS | |
| xylose | | | 543267 | 103 | SRBFZHDQGSBBOR-IOVATXLUSA-N | | | | GC-MS | |
| malic acid | | | 463180 | 233 | BJEPYKJPYRNKOW-UHFFFAOYSA-N | | | | GC-MS | |
| linoleic acid | | | 777414 | 150 | OYHQOLUKZRVURQ-HZJYTTRNSA-N | | | | GC-MS | |
| fumaric acid | | | 390775 | 245 | VZCYOOQTPOCHFL-OWOJBTEDSA-N | | | | GC-MS | |
| fructose | | | 641863 | 307 | RFSUNEUAIZKAJO-ARQDHWQXSA-N | | | | GC-MS | |
| adipic acid | | | 474435 | 111 | WNLRTRBMVRJNCN-UHFFFAOYSA-N | | | | GC-MS | |
| 2-hydroxyglutaric acid | | | 506306 | 247 | HWXBTNAVRSUOJR-UHFFFAOYSA-N | | | | GC-MS | |
| deoxycholic acid | | | 1106875 | 255 | KXGVEGMKQFWNSR-LLQZFEROSA-N | | | | GC-MS | |
| myristic acid | | | 634414 | 285 | TUNFSRHWOTWDNC-UHFFFAOYSA-N | | | | GC-MS | |
| glycerol-alpha-phosphate | | | 590747 | 357 | AWUCVROLDVIAJX-UHFFFAOYSA-N | | | | GC-MS | |
| glyceric acid | | | 377495 | 189 | RBNPOMFGQQGHHO-UWTATZPHSA-N | | | | GC-MS | |
| oxoproline | | | 485935 | 156 | ODHCTXKNWHHXJC-VKHMYHEASA-N | | | | GC-MS | |
| glycerol | | | 344466 | 205 | PEDCQBHIVMGVHV-UHFFFAOYSA-N | | | | GC-MS | |
| arachidic acid | | | 856421 | 117 | VKOBVWXKNCXXDE-UHFFFAOYSA-N | | | | GC-MS | |
| glutaric acid | | | 421260 | 261 | JFCQEDHGNNZCLN-UHFFFAOYSA-N | | | | GC-MS | |
| octadecanol | | | 755409 | 327 | GLDOVTGHNKAZLK-UHFFFAOYSA-N | | | | GC-MS | |
| tocopherol gamma- | | | 1026121 | 223 | QUEDXNHFTDJVIY-DQCZWYHMSA-N | | | | GC-MS | |
| threonic acid | | | 497572 | 292 | JPIJQSOTBSSVTP-STHAYSLISA-N | | | | GC-MS | |
| ribonic acid | | | 599680 | 292 | QXKAIJAYHKCRRA-BXXZVTAOSA-N | | | | GC-MS | |
| pentadecanoic acid | | | 674647 | 117 | WQEPLUUGTLDZJY-UHFFFAOYSA-N | | | | GC-MS | |
| uracil | | | 385735 | 241 | ISAKRJDGNUQOIC-UHFFFAOYSA-N | | | | GC-MS | |
| isoheptadecanoic acid NIST | | | 737927 | 117 | IIUXHTGBZYEGHI-UHFFFAOYSA-N | | | | GC-MS | |
| tagatose | | | 636627 | 307 | LKDRXBCSQODPBY-OEXCPVAWSA-N | | | | GC-MS | |
| succinic acid | | | 370608 | 247 | KDYFGRWQOYBRFD-UHFFFAOYSA-N | | | | GC-MS | |
| quinic acid | | | 632897 | 345 | AAWZDTNXLSGCEK-LNVDRNJUSA-N | | | | GC-MS | |
| phosphate | | | 345365 | 314 | NBIIXXVUZAFLBC-UHFFFAOYSA-N | | | | GC-MS | |
| lithocholic acid | | | 1104161 | 215 | SMEROWZSTRWXGI-HVATVPOCSA-N | | | | GC-MS | |
| tocopherol alpha- | | | 1067809 | 237 | NCYCYZXNIZJOKI-OVSJKPMPSA-N | | | | GC-MS | |
| thymine | | | 420133 | 255 | RWQNBRDOKXIBIV-UHFFFAOYSA-N | | | | GC-MS | |
| threonine | | | 409568 | 218 | AYFVYJQAPQTCCC-GBXIJSLDSA-N | | | | GC-MS | |
| ribose | | | 553135 | 217 | HMFHBZSHGGEWLO-SOOFDHNKSA-N | | | | GC-MS | |
| lauric acid | | | 547906 | 117 | POULHZVOKOAJMA-UHFFFAOYSA-N | | | | GC-MS | |
| glutamic acid | | | 529100 | 246 | WHUUTDBJXJRKMK-VKHMYHEASA-N | | | | GC-MS | |
| 4-hydroxyphenylacetic acid | | | 542795 | 179 | XQXPVVBIMDBYFF-UHFFFAOYSA-N | | | | GC-MS | |
| 1-hexadecanol | | | 679596 | 299 | BXWNKGSJHAJOGX-UHFFFAOYSA-N | | | | GC-MS | |
| valine | | | 309359 | 144 | KZSNJWFQEVHDMF-BYPYZUCNSA-N | | | | GC-MS | |
| tyrosine | | | 671252 | 218 | OUYCCCASQSFEME-QMMMGPOBSA-N | | | | GC-MS | |
| leucine | | | 346101 | 158 | ROHFNLRQFUQHCH-YFKPBYRVSA-N | | | | GC-MS | |
| isoleucine | | | 359251 | 158 | AGPKZVBTJJNPAG-WHFBIAKZSA-N | | | | GC-MS | |
| phenylalanine | | | 537804 | 218 | COLNVLDHVKWLRT-QMMMGPOBSA-N | | | | GC-MS | |
| N-acetylglutamate | | | 604748 | 216 | RFMMMVDNIPUKGG-YFKPBYRVSA-N | | | | GC-MS | |
| n-acetyl-d-hexosamine | | | 746341 | 319 | OVRNDRQMDRJTHS-FMDGEEDCSA-N | | | | GC-MS | |
| lysine | | | 663483 | 156 | KDXKERNSBIXSRK-YFKPBYRVSA-N | | | | GC-MS | |
| hypoxanthine | | | 619107 | 265 | FDGQSTZJBFJUBT-UHFFFAOYSA-N | | | | GC-MS | |
| aspartic acid | | | 480387 | 232 | CKLJMWTZIZZHCS-REOHCLBHSA-N | | | | GC-MS | |
| isopentadecanoic acid | | | 663518 | 117 | ZOCYQVNGROEVLU-UHFFFAOYSA-N | | | | GC-MS | |
| cellobiose | | | 932179 | 204 | GUBGYTABKSRVRQ-QUYVBRFLSA-N | | | | GC-MS | |
| 5-aminovaleric acid | | | 540425 | 174 | JJMDCOVWQOJGCB-UHFFFAOYSA-N | | | | GC-MS | |
| 4-hydroxybenzoate | | | 537925 | 223 | FJKROLUGYXJWQN-UHFFFAOYSA-N | | | | GC-MS | |
| 3,4-dihydroxycinnamic acid | | | 748847 | 219 | QAIPRVGONGVQAS-DUXPYHPUSA-N | | | | GC-MS | |
| 1-monopalmitin | | | 901749 | 129 | QHZLMUACJMDIAE-UHFFFAOYSA-N | | | | GC-MS | |
| serine | | | 395020 | 218 | MTCFGRXMJLQNBG-REOHCLBHSA-N | | | | GC-MS | |
| proline | | | 364523 | 142 | ONIBWKKTOPOVIA-BYPYZUCNSA-N | | | | GC-MS | |
| beta-alanine | | | 435564 | 248 | UCMIRNVEIXFBKS-UHFFFAOYSA-N | | | | GC-MS | |
| lyxose | | | 541086 | 217 | SRBFZHDQGSBBOR-AGQMPKSLSA-N | | | | GC-MS | |
| uric acid | | | 730691 | 441 | LEHOTFFKMJEONL-UHFFFAOYSA-N | | | | GC-MS | |
| thymidine | | | 349402 | 170 | IQFYYKKMVGJFEH-XLPZGREQSA-N | | | | GC-MS | |
| xanthine | | | 701688 | 353 | LRFVTYWOQMYALW-UHFFFAOYSA-N | | | | GC-MS | |
| pantothenic acid | | | 690887 | 291 | GHOKWGTUZJEAQD-ZETCQYMHSA-N | | | | GC-MS | |
| glycine | | | 368707 | 248 | DHMQDGOQFOQNFH-UHFFFAOYSA-N | | | | GC-MS | |
| 3,6-anhydro-D-galactose | | | 588886 | 231 | WZYRMLAWNVOIEX-BGPJRJDNSA-N | | | | GC-MS | |
| lignoceric acid | | | 978597 | 132 | QZZGJDVWLFXDLK-UHFFFAOYSA-N | | | | GC-MS | |
| 1-monoolein | | | 955584 | 129 | RZRNAYUHWVFMIP-KTKRTIGZSA-N | | | | GC-MS | |
| inosine | | | 897184 | 230 | UGQMRVRMYYASKQ-KQYNXXCUSA-N | | | | GC-MS | |
| hydroquinone | | | 422583 | 239 | QIGBRXMKCJKVMJ-UHFFFAOYSA-N | | | | GC-MS | |
| indole-3-acetate | | | 684929 | 202 | SEOVTRFCIGRIMH-UHFFFAOYSA-N | | | | GC-MS | |
| nicotinic acid | | | 366992 | 180 | PVNIIMVLHYAWGP-UHFFFAOYSA-N | | | | GC-MS | |
| methionine sulfoxide | | | 637588 | 128 | QEFRNWWLZKMPFJ-YGVKFDHGSA-N | | | | GC-MS | |
| citramalic acid | | | 456203 | 247 | XFTRTWQBIOMVPK-UHFFFAOYSA-N | | | | GC-MS | |
| phenol | | | 218927 | 151 | ISWSIDIOOBJBQZ-UHFFFAOYSA-N | | | | GC-MS | |
| ketohexose | | | 627414 | 307 | BJHIKXHVCXFQLS-PUFIMZNGSA-N | | | | GC-MS | |
| galacturonic acid | | | 669400 | 333 | AEMOLEFTQBMNLQ-DTEWXJGMSA-N | | | | GC-MS | |
| 3,4-dihydroxyhydrocinnamic  acid NIST | | | 673176 | 179 | DZAUWHJDUNRCTF-UHFFFAOYSA-N | | | | GC-MS | |
| ferulic acid | | | 732779 | 338 | KSEBMYQBYZTDHS-HWKANZROSA-N | | | | GC-MS | |
| 4-hydroxybutyric acid | | | 325027 | 233 | SJZRECIVHVDYJC-UHFFFAOYSA-N | | | | GC-MS | |
| putrescine | | | 588119 | 174 | KIDHWZJUCRJVML-UHFFFAOYSA-N | | | | GC-MS | |
| 4-pyridoxic acid | | | 673225 | 309 | HXACOUQIXZGNBF-UHFFFAOYSA-N | | | | GC-MS | |
| maltose | | | 946601 | 204 | GUBGYTABKSRVRQ-PICCSMPSSA-N | | | | GC-MS | |
| beta-glutamic acid | | | 525547 | 232 | BBJIPMIXTXKYLZ-UHFFFAOYSA-N | | | | GC-MS | |
| behenic acid | | | 920648 | 117 | UKMSUNONTOPOIO-UHFFFAOYSA-N | | | | GC-MS | |
| propane-1,3-diol NIST | | | 214380 | 177 | YPFDHNVEDLHUCE-UHFFFAOYSA-N | | | | GC-MS | |
| cis-gondoic acid | | | 847372 | 367 | BITHHVVYSMSWAG-KTKRTIGZSA-N | | | | GC-MS | |
| sulfurol | | | 408609 | 103 | BKAWJIRCKVUVED-UHFFFAOYSA-N | | | | GC-MS | |
| pseudo uridine | | | 813899 | 217 | PTJWIQPHWPFNBW-GBNDHIKLSA-N | | | | GC-MS | |
| methionine | | | 483560 | 176 | FFEARJCKVFRZRR-BYPYZUCNSA-N | | | | GC-MS | |
| alanine | | | 243971 | 116 | QNAYBMKLOCPYGJ-REOHCLBHSA-N | | | | GC-MS | |
| maltotriose | | | 1176044 | 361 | FYGDTMLNYKFZSV-DZOUCCHMSA-N | | | | GC-MS | |
| glutamine | | | 600000 | 156 | ZDXPYRJPNDTMRX-VKHMYHEASA-N | | | | GC-MS | |
| urocanic acid | | | 699866 | 267 | LOIYMIARKYCTBW-OWOJBTEDSA-N | | | | GC-MS | |
| pyruvic acid | | | 213805 | 174 | LCTONWCANYUPML-UHFFFAOYSA-N | | | | GC-MS | |
| pipecolinic acid | | | 404121 | 156 | HXEACLLIILLPRG-RXMQYKEDSA-N | | | | GC-MS | |
| levoglucosan | | | 569637 | 204 | TWNIBLMWSKIRAT-VFUOTHLCSA-N | | | | GC-MS | |
| adenosine | | | 918039 | 236 | OIRDTQYFTABQOQ-KQYNXXCUSA-N | | | | GC-MS | |
| guanosine | | | 954962 | 324 | NYHBQMYGNKIUIF-UUOKFMHZSA-N | | | | GC-MS | |
| orotic acid | | | 586317 | 254 | PXQPEWDEAKTCGB-UHFFFAOYSA-N | | | | GC-MS | |
| citric acid | | | 617342 | 273 | KRKNYBCHXYNGOX-UHFFFAOYSA-N | | | | GC-MS | |
| alanine-alanine | | | 523110 | 116 | DEFJQIDDEAULHB-IMJSIDKUSA-N | | | | GC-MS | |
| 3-hydroxypropionic acid | | | 269265 | 177 | ALRHLSYJTWAHJZ-UHFFFAOYSA-N | | | | GC-MS | |
| hydroxycarbamate NIST | | | 325948 | 278 | DRAJWRKLRBNJRQ-UHFFFAOYSA-M | | | | GC-MS | |
| cholic acid | | | 1109517 | 253 | BHQCQFFYRZLCQQ-OELDTZBJSA-N | | | | GC-MS | |
| adenine | | | 646701 | 264 | GFFGJBXGBJISGV-UHFFFAOYSA-N | | | | GC-MS | |
| 3,4-dihydroxybenzoic acid | | | 620200 | 193 | YQUVCSBJEUQKSH-UHFFFAOYSA-N | | | | GC-MS | |
| tryptophan | | | 780482 | 202 | QIVBCDIJIAJPQS-VIFPVBQESA-N | | | | GC-MS | |
| pyridoxine | | | 653546 | 280 | LXNHXLLTXMVWPM-UHFFFAOYSA-N | | | | GC-MS | |
| N-acetylornithine | | | 694651 | 174 | JRLGPAXAGHMNOL-LURJTMIESA-N | | | | GC-MS | |
| aminomalonate | | | 455754 | 218 | JINBYESILADKFW-UHFFFAOYSA-N | | | | GC-MS | |
| phytanic acid | | | 770604 | 159 | RLCKHJSFHOZMDR-GUDVDZBRSA-N | | | | GC-MS | |
| parabanic acid NIST | | | 464991 | 100 | ZFLIKDUSUDBGCD-UHFFFAOYSA-N | | | | GC-MS | |
| glucose | | | 678811 | 217 |  | | |  | GC-MS | |
| 2'-deoxyguanosine | | | 959764 | 280 | YKBGVTZYEHREMT-KVQBGUIXSA-N | | | | GC-MS | |
| pimelic acid | | | 523205 | 155 | WLJVNTCWHIRURA-UHFFFAOYSA-N | | | | GC-MS | |
| lactic acid | | | 217657 | 191 | JVTAAEKCZFNVCJ-UHFFFAOYSA-N | | | | GC-MS | |
| guanine | | | 744307 | 352 | UYTPUPDQBNUYGX-UHFFFAOYSA-N | | | | GC-MS | |
| tartaric acid | | | 534291 | 292 | FEWJPZIEWOKRBE-JCYAYHJZSA-N | | | | GC-MS | |
| UDP-glucuronic acid | | | 587601 | 217 | HDYANYHVCAPMJV-LXQIFKJMSA-N | | | | GC-MS | |
| tyrosol | | | 510842 | 179 | YCCILVSKPBXVIP-UHFFFAOYSA-N | | | | GC-MS | |
| nonadecanoic acid | | | 822782 | 117 | ISYWECDDZWTKFF-UHFFFAOYSA-N | | | | GC-MS | |
| fucose | | | 584895 | 117 | SHZGCJCMOBCMKK-FPRJBGLDSA-N | | | | GC-MS | |
| arachidonic acid | | | 834339 | 91 | YZXBAPSDXZZRGB-DOFZRALJSA-N | | | | GC-MS | |
| 3-hydroxypalmitic acid | | | 774930 | 233 | CBWALJHXHCJYTE-OAHLLOKOSA-N | | | | GC-MS | |
| 2-monoolein | | | 941794 | 129 | UPWGQKDVAURUGE-KTKRTIGZSA-N | | | | GC-MS | |
| oxalic acid | | | 260513 | 190 | MUBZPKHOEPUJKR-UHFFFAOYSA-N | | | | GC-MS | |
| hexadecylglycerol NIST | | | 867593 | 205 | OOWQBDFWEXAXPB-UHFFFAOYSA-N | | | | GC-MS | |
| 6-hydroxynicotinic acid | | | 510614 | 268 | BLHCMGRVFXRYRN-UHFFFAOYSA-N | | | | GC-MS | |
| 2-hydroxyhexanoic acid | | | 323093 | 159 | NYHNVHGFPZAZGA-UHFFFAOYSA-N | | | | GC-MS | |
| homoserine | | | 443878 | 218 | UKAUYVFTDYCKQA-VKHMYHEASA-N | | | | GC-MS | |
| 3-hydroxybutyric acid | | | 278632 | 191 | WHBMMWSBFZVSSR-GSVOUGTGSA-N | | | | GC-MS | |
| 3-(4-hydroxyphenyl)propionic acid | | | 598147 | 179 | NMHMNPHRMNGLLB-UHFFFAOYSA-N | | | | GC-MS | |
| raffinose | | | 1120886 | 361 | MUPFEKGTMRGPLJ-ZQSKZDJDSA-N | | | | GC-MS | |
| mannitol | | | 663215 | 319 | FBPFZTCFMRRESA-KVTDHHQDSA-N | | | | GC-MS | |
| cysteine | | | 500158 | 220 | XUJNEKJLAYXESH-REOHCLBHSA-N | | | | GC-MS | |
| phenylacetic acid | | | 368081 | 164 | WLJVXDMOQOGPHL-UHFFFAOYSA-N | | | | GC-MS | |
| chenodeoxycholic acid | | | 1114867 | 255 | RUDATBOHQWOJDD-BSWAIDMHSA-N | | | | GC-MS | |
| urea | | | 328888 | 189 | XSQUKJJJFZCRTK-UHFFFAOYSA-N | | | | GC-MS | |
| 3-phenyllactic acid | | | 516011 | 193 | VOXXWSYKYCBWHO-UHFFFAOYSA-N | | | | GC-MS | |
| 3-hydroxyphenylacetic acid | | | 527648 | 164 | FVMDYYGIDFPZAX-UHFFFAOYSA-N | | | | GC-MS | |
| xanthosine | | | 926133 | 325 | UBORTCNDUKBEOP-UUOKFMHZSA-N | | | | GC-MS | |
| mannose | | | 645856 | 205 | WQZGKKKJIJFFOK-QTVWNMPRSA-N | | | | GC-MS | |
| enolpyruvate NIST | | | 234394 | 217 | DTBNBXWJWCWCIK-UHFFFAOYSA-N | | | | GC-MS | |
| 5'-deoxy-5'-methylthioadenosine | | | 967036 | 236 | WUUGFSXJNOTRMR-IOSLPCCCSA-N | | | | GC-MS | |
| 3-(3-hydroxyphenyl)propionic acid | | | 583925 | 192 | QVWAEZJXDYOKEH-UHFFFAOYSA-N | | | | GC-MS | |
| 2-methylglutaric acid | | | 427677 | 172 | XJMMNTGIMDZPMU-UHFFFAOYSA-N | | | | GC-MS | |
| ethanolamine | | | 344719 | 174 | HZAXFHJVJLSVMW-UHFFFAOYSA-N | | | | GC-MS | |
| docosahexaenoic acid | | | 902819 | 91 | MBMBGCFOFBJSGT-KUBAVDMBSA-N | | | | GC-MS | |
| cerotinic acid | | | 1033286 | 145 | XMHIUKTWLZUKEX-UHFFFAOYSA-N | | | | GC-MS | |
| 2-hydroxybutanoic acid | | | 258161 | 131 | AFENDNXGAFYKQO-VKHMYHEASA-N | | | | GC-MS | |
| 2-deoxyerythritol | | | 355045 | 117 | ARXKVVRQIIOZGF-UHFFFAOYSA-N | | | | GC-MS | |
| N-acetylputrescine | | | 595523 | 174 | KLZGKIDSEJWEDW-UHFFFAOYSA-N | | | | GC-MS | |
| lanosterol | | | 1129573 | 393 | CAHGCLMLTWQZNJ-BQNIITSRSA-N | | | | GC-MS | |
| hydrocinnamic acid | | | 434422 | 104 | XMIIGOLPHOKFCH-UHFFFAOYSA-N | | | | GC-MS | |
| cytosin | | | 486724 | 254 | OPTASPLRGRRNAP-UHFFFAOYSA-N | | | | GC-MS | |
| 3-hydroxy-3-methylglutaric acid | | | 521554 | 247 | NPOAOTPXWNWTSH-UHFFFAOYSA-N | | | | GC-MS | |
| 2-monopalmitin | | | 890356 | 129 | BBNYCLAREVXOSG-UHFFFAOYSA-N | | | | GC-MS | |
| hexuronic acid | | | 673957 | 333 | IAJILQKETJEXLJ-UHFFFAOYSA-N | | | | GC-MS | |
| trans-4-hydroxy-L-proline | | | 459475 | 158 | PMMYEEVYMWASQN-DMTCNVIQSA-N | | | | GC-MS | |
| glucose-1-phosphate | | | 594647 | 217 | HXXFSFRBOHSIMQ-VFUOTHLCSA-N | | | | GC-MS | |
| gluconic acid | | | 693148 | 333 | RGHNJXZEOKUKBD-QTBDOELSSA-N | | | | GC-MS | |
| galactonic acid | | | 690882 | 292 | RGHNJXZEOKUKBD-MGCNEYSASA-N | | | | GC-MS | |
| 3-aminoisobutyric acid | | | 452655 | 248 | QCHPKSFMDHPSNR-UHFFFAOYSA-N | | | | GC-MS | |
| pyrogallol | | | 495011 | 239 | WQGWDDDVZFFDIG-UHFFFAOYSA-N | | | | GC-MS | |
| maleic acid | | | 363611 | 245 | VZCYOOQTPOCHFL-UPHRSURJSA-N | | | | GC-MS | |
| lactitol | | | 959541 | 361 | VQHSOMBJVWLPSR-JVCRWLNRSA-N | | | | GC-MS | |
| dihydrocholesterol | | | 1082070 | 215 | QYIXCDOBOSTCEI-FBVYSKEZSA-N | | | | GC-MS | |
| D-erythro-sphingosine | | | 859500 | 204 | WWUZIQQURGPMPG-KRWOKUGFSA-N | | | | GC-MS | |
| creatinine | | | 502599 | 115 | DDRJAANPRJIHGJ-UHFFFAOYSA-N | | | | GC-MS | |
| 2-deoxytetronic acid | | | 433456 | 189 | DZAIOXUZHHTJKN-UHFFFAOYSA-N | | | | GC-MS | |
| 1,2,4-benzenetriol | | | 521803 | 239 | GGNQRNBDZQJCCN-UHFFFAOYSA-N | | | | GC-MS | |
| xylitol | | | 567437 | 217 | HEBKCHPVOIAQTA-NGQZWQHPSA-N | | | | GC-MS | |
| uridine | | | 861508 | 217 | DRTQHJPVMGBUCF-XVFCMESISA-N | | | | GC-MS | |
| phenylethylamine | | | 510327 | 174 | BHHGXPLMPWCGHP-UHFFFAOYSA-N | | | | GC-MS | |
| malonic acid | | | 305372 | 233 | OFOBLEOULBTSOW-UHFFFAOYSA-N | | | | GC-MS | |
| glycerol-3-galactoside | | | 800205 | 204 | NHJUPBDCSOGIKX-NTXXKDEISA-N | | | | GC-MS | |
| glucose-6-phosphate | | | 818275 | 387 | NBSCHQHZLSJFNQ-GASJEMHNSA-N | | | | GC-MS | |
| conduritol-beta-epoxide | | | 675635 | 318 | ZHMWOVGZCINIHW-SPHYCDKFSA-N | | | | GC-MS | |
| butyrolactam NIST | | | 277199 | 142 | HNJBEVLQSNELDL-UHFFFAOYSA-N | | | | GC-MS | |
| 3,4-dihydroxyphenylacetic acid | | | 625046 | 179 | CFFZDZCDUFSOFZ-UHFFFAOYSA-N | | | | GC-MS | |
| threitol | | | 467595 | 217 | UNXHWFMMPAWVPI-QWWZWVQMSA-N | | | | GC-MS | |
| piperidone | | | 275603 | 156 | XUWHAWMETYGRKB-UHFFFAOYSA-N | | | | GC-MS | |
| phthalic acid | | | 567345 | 147 | XNGIFLGASWRNHJ-UHFFFAOYSA-N | | | | GC-MS | |
| maltotriitol | | | 1216941 | 361 |  | | |  | GC-MS | |
| beta-gentiobiose | | | 965404 | 160 | DLRVVLDZNNYCBX-LIZSDCNHSA-N | | | | GC-MS | |
| arabitol | | | 570685 | 217 | HEBKCHPVOIAQTA-QWWZWVQMSA-N | | | | GC-MS | |
| xylonic acid isomer | | | 590775 | 189 | QXKAIJAYHKCRRA-UHFFFAOYSA-N | | | | GC-MS | |
| N-acetylaspartic acid | | | 548028 | 158 | OTCCIMWXFLJLIA-BYPYZUCNSA-N | | | | GC-MS | |
| mucic acid | | | 709842 | 333 | DSLZVSRJTYRBFB-DUHBMQHGSA-N | | | | GC-MS | |
| mannonic acid NIST | | | 689527 | 333 | RGHNJXZEOKUKBD-MBMOQRBOSA-N | | | | GC-MS | |
| isomaltose | | | 983199 | 160 | DLRVVLDZNNYCBX-RTPHMHGBSA-N | | | | GC-MS | |
| glycyl-proline | | | 691662 | 174 | KZNQNBZMBZJQJO-YFKPBYRVSA-N | | | | GC-MS | |
| galactinol | | | 1017580 | 204 | VCWMRQDBPZKXKG-ZNVDUFQESA-N | | | | GC-MS | |
| cyclohexylamine | | | 225912 | 128 | PAFZNILMFXTMIY-UHFFFAOYSA-N | | | | GC-MS | |
| caprylic acid | | | 343457 | 201 | WWZKQHOCKIZLMA-UHFFFAOYSA-N | | | | GC-MS | |
| asparagine | | | 553078 | 188 | DCXYFEDJOCDNAF-REOHCLBHSA-N | | | | GC-MS | |
| vanillic acid | | | 597845 | 297 | WKOLLVMJNQIZCI-UHFFFAOYSA-N | | | | GC-MS | |
| phosphoethanolamine | | | 603912 | 299 | SUHOOTKUPISOBE-UHFFFAOYSA-N | | | | GC-MS | |
| 5-hydroxymethyl-2-furoic acid NIST | | | 497561 | 123 | PCSKKIUURRTAEM-UHFFFAOYSA-N | | | | GC-MS | |
| spermidine | | | 792924 | 144 | ATHGHQPFGPMSJY-UHFFFAOYSA-N | | | | GC-MS | |
| pinitol | | | 622466 | 260 | DSCFFEYYQKSRSV-FEPQRWDDSA-N | | | | GC-MS | |
| monomyristin | | | 838305 | 343 | DCBSHORRWZKAKO-UHFFFAOYSA-N | | | | GC-MS | |
| indole-3-propionic acid | | | 732249 | 202 | GOLXRNDWAUTYKT-UHFFFAOYSA-N | | | | GC-MS | |
| glucoheptulose | | | 828606 | 217 | HSNZZMHEPUFJNZ-SHUUEZRQSA-N | | | | GC-MS | |
| 6-deoxyglucose | | | 573663 | 117 | SHZGCJCMOBCMKK-DVKNGEFBSA-N | | | | GC-MS | |
| 5-methoxytryptamine | | | 864466 | 174 | JTEJPPKMYBDEMY-UHFFFAOYSA-N | | | | GC-MS | |
| 4-methylcatechol | | | 416586 | 268 | ZBCATMYQYDCTIZ-UHFFFAOYSA-N | | | | GC-MS | |
| 2-deoxypentitol NIST | | | 521785 | 231 | ZDAWZDFBPUUDAY-UHFFFAOYSA-N | | | | GC-MS | |
| xylonolactone NIST | | | 535176 | 217 | XXBSUZSONOQQGK-FLRLBIABSA-N | | | | GC-MS | |
| tyramine | | | 664737 | 174 | DZGWFCGJZKJUFP-UHFFFAOYSA-N | | | | GC-MS | |
| tranexamic acid NIST | | | 565931 | 174 | GYDJEQRTZSCIOI-UHFFFAOYSA-N | | | | GC-MS | |
| sorbitol | | | 667922 | 217 | FBPFZTCFMRRESA-JGWLITMVSA-N | | | | GC-MS | |
| phytosphingosine | | | 910976 | 204 | AERBNCYCJBRYDG-KSZLIROESA-N | | | | GC-MS | |
| montanic acid | | | 1087377 | 117 | UTOPWMOLSKOLTQ-UHFFFAOYSA-N | | | | GC-MS | |
| linolenic acid | | | 780376 | 108 | DTOSIQBPPRVQHS-PDBXOOCHSA-N | | | | GC-MS | |
| inositol-4-monophosphate | | | 845976 | 315 | INAPMGSXUVUWAF-GFWFORPUSA-N | | | | GC-MS | |
| 2-aminophenol | | | 438445 | 150 | CDAWCLOXVUBKRW-UHFFFAOYSA-N | | | | GC-MS | |
| 2,8-dihydroxyquinoline | | | 626989 | 290 | ZXZKYYHTWHJHFT-UHFFFAOYSA-N | | | | GC-MS | |
| saccharopine | | | 858549 | 357 | ZDGJAHTZVHVLOT-YUMQZZPRSA-N | | | | GC-MS | |
| p-hydroxylphenyllactic acid | | | 654036 | 308 | JVGVDSSUAVXRDY-UHFFFAOYSA-N | | | | GC-MS | |
| mannose-6-phosphate NIST | | | 822643 | 387 | NBSCHQHZLSJFNQ-QTVWNMPRSA-N | | | | GC-MS | |
| isoribose | | | 558730 | 217 | SRBFZHDQGSBBOR-SOOFDHNKSA-N | | | | GC-MS | |
| indole-3-lactate | | | 764586 | 202 | XGILAAMKEQUXLS-UHFFFAOYSA-N | | | | GC-MS | |
| 2-hydroxyvaleric acid | | | 309587 | 131 | JRHWHSJDIILJAT-UHFFFAOYSA-N | | | | GC-MS | |
| 2-aminobutyric acid | | | 285825 | 130 | QWCKQJZIFLGMSD-UHFFFAOYSA-N | | | | GC-MS | |
| 2,3-dihydro-8-methoxyfuro(2,3-b)quinoline NIST | | | 525108 | 200 |  | | |  | GC-MS | |
| sulfuric acid | | | 285430 | 227 | QAOWNCQODCNURD-UHFFFAOYSA-N | | | | GC-MS | |
| saccharic acid | | | 699211 | 333 | DSLZVSRJTYRBFB-LLEIAEIESA-N | | | | GC-MS | |
| ribitol | | | 575497 | 217 | HEBKCHPVOIAQTA-ZXFHETKHSA-N | | | | GC-MS | |
| melezitose | | | 1146704 | 361 | QWIZNVHXZXRPDR-WSCXOGSTSA-N | | | | GC-MS | |
| isolinoleic acid NIST | | | 794629 | 156 | ZMKDEQUXYDZSNN-UTJQPWESSA-N | | | | GC-MS | |
| deoxypentitol | | | 528774 | 231 | FJGNTEKSQVNVTJ-UHFFFAOYSA-N | | | | GC-MS | |
| alpha-aminoadipic acid | | | 573295 | 260 | OYIFNHCXNCRBQI-BYPYZUCNSA-N | | | | GC-MS | |
| 7-methylguanine NIST | | | 768706 | 294 | FZWGECJQACGGTI-UHFFFAOYSA-N | | | | GC-MS | |
| 4-aminobutyric acid | | | 488730 | 304 | BTCSSZJGUNDROE-UHFFFAOYSA-N | | | | GC-MS | |
| 2-picolinic acid | | | 383668 | 180 | SIOXPEMLGUPBBT-UHFFFAOYSA-N | | | | GC-MS | |
| trehalose | | | 948197 | 191 | HDTRYLNUVZCQOY-LIZSDCNHSA-N | | | | GC-MS | |
| tocopherol delta- NIST | | | 997765 | 208 | GZIFEOYASATJEH-VHFRWLAGSA-N | | | | GC-MS | |
| taurine | | | 556690 | 326 | XOAAWQZATWQOTB-UHFFFAOYSA-N | | | | GC-MS | |
| sophorose | | | 965726 | 307 | HIWPGCMGAMJNRG-BTLHAWITSA-N | | | | GC-MS | |
| shikimic acid | | | 611100 | 204 | JXOHGGNKMLTUBP-HSUXUTPPSA-N | | | | GC-MS | |
| salicylic acid | | | 480699 | 267 | YGSDEFSMJLZEOE-UHFFFAOYSA-N | | | | GC-MS | |
| oleamide NIST | | | 849710 | 144 | FATBGEAMYMYZAF-KTKRTIGZSA-N | | | | GC-MS | |
| lactobionic acid | | | 972139 | 204 | JYTUSYBCFIZPBE-AMTLMPIISA-N | | | | GC-MS | |
| galactose-6-phosphate | | | 828102 | 387 | NBSCHQHZLSJFNQ-SVZMEOIVSA-N | | | | GC-MS | |
| cystine | | | 804619 | 218 | LEVWYRKDKASIDU-UHFFFAOYSA-N | | | | GC-MS | |
| cis-caffeic acid | | | 688023 | 219 | QAIPRVGONGVQAS-RQOWECAXSA-N | | | | GC-MS | |
| arabinose | | | 550621 | 217 | SRBFZHDQGSBBOR-ZRMNMSDTSA-N | | | | GC-MS | |
| 2-methylglyceric acid NIST | | | 372491 | 219 | DGADNPLBVRLJGD-UHFFFAOYSA-N | | | | GC-MS | |
| 2-hydroxyadipic acid | | | 549663 | 261 | OTTXIFWBPRRYOG-UHFFFAOYSA-N | | | | GC-MS | |
| zymosterol | | | 1088064 | 129 | CGSJXLIKVBJVRY-XTGBIJOFSA-N | | | | GC-MS | |
| tocopherol beta NIST | | | 1022815 | 223 | WGVKWNUPNGFDFJ-CBIUGAAKSA-N | | | | GC-MS | |
| salicylaldehyde | | | 406586 | 193 | SMQUZDBALVYZAC-UHFFFAOYSA-N | | | | GC-MS | |
| pentitol | | | 563801 | 307 | HEBKCHPVOIAQTA-UHFFFAOYSA-N | | | | GC-MS | |
| methylmaleic acid | | | 418804 | 259 | HNEGQIOMVPPMNR-IHWYPQMZSA-N | | | | GC-MS | |
| isohexonic acid | | | 698302 | 333 | RGHNJXZEOKUKBD-UHFFFAOYSA-N | | | | GC-MS | |
| gluconic acid lactone | | | 645815 | 220 | PHOQVHQSTUBQQK-SQOUGZDYSA-N | | | | GC-MS | |
| erythrose | | | 443306 | 205 | FMAORJIQYMIRHF-HERZVMAMSA-N | | | | GC-MS | |
| elaidic acid | | | 784781 | 129 | ZQPPMHVWECSIRJ-MDZDMXLPSA-N | | | | GC-MS | |
| dehydroabietic acid | | | 850374 | 239 | NFWKVWVWBFBAOV-MISYRCLQSA-N | | | | GC-MS | |
| citrulline | | | 589262 | 184 | RHGKLRLOHDJJDR-BYPYZUCNSA-N | | | | GC-MS | |
| beta-glycerolphosphate | | | 574470 | 243 | DHCLVCXQIBBOPH-UHFFFAOYSA-N | | | | GC-MS | |
| allantoic acid | | | 723539 | 259 | NUCLJNSWZCHRKL-UHFFFAOYSA-N | | | | GC-MS | |
| 4-aminobenzoicacid | | | 630439 | 266 |  | | | | GC-MS | |
| 3-hydroxybenzoic acid | | | 507599 | 267 | IJFXRHURBJZNAO-UHFFFAOYSA-N | | | | GC-MS | |
| 2,4-hexadienedioic acid NIST | | | 550289 | 271 | TXXHDPDFNKHHGW-CCAGOZQPSA-N | | | | GC-MS | |
| tocopherol acetate | | | 1081080 | 164 | ZAKOWWREFLAJOT-CEFNRUSXSA-N | | | | GC-MS | |
| nicotinamide | | | 471602 | 179 | DFPAKSUCGFBDDF-UHFFFAOYSA-N | | | | GC-MS | |
| maleimide | | | 245118 | 154 | PEEHTFAAVSWFBL-UHFFFAOYSA-N | | | | GC-MS | |
| isocitric acid | | | 616323 | 245 | ODBLHEXUDAPZAU-ZAFYKAAXSA-N | | | | GC-MS | |
| epicatechin | | | 979335 | 368 | PFTAWBLQPZVEMU-UKRRQHHQSA-N | | | | GC-MS | |
| daidzein | | | 1013259 | 398 | ZQSIJRDFPHDXIC-UHFFFAOYSA-N | | | | GC-MS | |
| adenosine-5-monophosphate | | | 1038688 | 169 | UDMBCSSLTHHNCD-KQYNXXCUSA-N | | | | GC-MS | |
| 6-deoxyhexitol NIST | | | 593784 | 319 |  | | |  | GC-MS | |
| 2,3-dihydroxybutanoic acid NIST | | | 384796 | 292 | LOUGYXZSURQALL-UHFFFAOYSA-N | | | | GC-MS | |
| 1-kestose | | | 1123027 | 361 | VAWYEUIPHLMNNF-OESPXIITSA-N | | | | GC-MS | |
| melibiose | | | 988497 | 361 | DLRVVLDZNNYCBX-ABXHMFFYSA-N | | | | GC-MS | |
| lyxitol | | | 573587 | 217 | HEBKCHPVOIAQTA-IMJSIDKUSA-N | | | | GC-MS | |
| p-cresol | | | 280360 | 165 | WVDDGKGOMKODPV-UHFFFAOYSA-N | | | | GC-MS | |
| methanolphosphate | | | 289520 | 241 | CAAULPUQFIIOTL-UHFFFAOYSA-N | | | | GC-MS | |
| histidine | | | 664395 | 154 | HNDVDQJCIGZPNO-YFKPBYRVSA-N | | | | GC-MS | |
| 3-(3-hydroxyphenyl)-3-hydroxypropionic acid nist | | | 632357 | 267 | KHTAGVZHYUZYMF-UHFFFAOYSA-N | | | | GC-MS | |
| 1-methylhydantoin | | | 381440 | 100 | RHYBFKMFHLPQPH-UHFFFAOYSA-N | | | | GC-MS | |
| pyrophosphate | | | 547021 | 451 | XPPKVPWEQAFLFU-UHFFFAOYSA-N | | | | GC-MS | |
| N-acetylglycine NIST | | | 356109 | 174 | OKJIRPAQVSHGFK-UHFFFAOYSA-N | | | | GC-MS | |
| hydroxyproline dipeptide NIST | | | 879596 | 156 | HXWNRESJYUCPRA-UHFFFAOYSA-N | | | | GC-MS | |
| glutamyl-valine | | | 760894 | 156 | SITLTJHOQZFJGG-XPUUQOCRSA-N | | | | GC-MS | |
| epsilon-caprolactam | | | 353069 | 170 | JBKVHLHDHHXQEQ-UHFFFAOYSA-N | | | | GC-MS | |
| butylamine | | | 249493 | 174 | HQABUPZFAYXKJW-UHFFFAOYSA-N | | | | GC-MS | |
| biuret | | | 571466 | 171 | OGNSCSPNOLGXSM-VKHMYHEASA-N | | | | GC-MS | |
| 5-hydroxynorvaline NIST | | | 494077 | 142 | CZWARROQQFCFJB-UHFFFAOYSA-N | | | | GC-MS | |
| 1,5-anhydroglucitol | | | 633603 | 217 | MPCAJMNYNOGXPB-SLPGGIOYSA-N | | | | GC-MS | |

| Table S9. Significantly altered metabolites in alcoholic hepatitis patients with cirrhosis compared with alcohol use disorder patients with cirrhosis | | | | |
| --- | --- | --- | --- | --- |
| compound_names | p_value | p_value_adj | FoldChange | log2FoldChange |
| Diazepam | 0.02380952 | 0.14606742 | 0371479 | -8.0725026 |
| oleamide NIST | 0.02380952 | 0.14606742 | 0.05079817 | -4.2990796 |
| trans-Nicotine-1'-oxide | 0.02380952 | 0.14606742 | 0.07990141 | -3.6456353 |
| trans-3'-Hydroxycotinine | 0.02380952 | 0.14606742 | 0.08197059 | -3.6087499 |
| gamma-Glutamylleucine | 0.02380952 | 0.14606742 | 0.11034654 | -3.1798867 |
| indole-3-propionic acid | 0.02380952 | 0.14606742 | 0.11331957 | -3.1415311 |
| 3-(4-hydroxyphenyl)propionic acid | 0.02753189 | 0.16163886 | 0.11650485 | -3.101538 |
| isolinoleic acid NIST | 0.02380952 | 0.14606742 | 0.11876833 | -3.0737779 |
| N-acetylglycine NIST | 0.02380952 | 0.14606742 | 0.12966829 | -2.9471024 |
| Phe-Phe | 0.02380952 | 0.14606742 | 0.13272214 | -2.9135191 |
| Phe-Arg | 0.02380952 | 0.14606742 | 0.1424224 | -2.811752 |
| Arg-Phe | 0.02380952 | 0.14606742 | 0.14259928 | -2.8099614 |
| deoxycholic acid | 0.02380952 | 0.14606742 | 0.1617284 | -2.6283551 |
| Serotonin | 0.02380952 | 0.14606742 | 0.2093916 | -2.2557245 |
| .beta.-Hyodeoxycholic acid | 0.02380952 | 0.14606742 | 0.22367516 | -2.1605231 |
| Triptolide | 0.02380952 | 0.14606742 | 0.2266147 | -2.1416867 |
| Phe-Trp | 0.02380952 | 0.14606742 | 0.22673358 | -2.14093 |
| Norleucine | 0.04761905 | 0.20634921 | 0.26985531 | -1.889742 |
| 1,5-anhydroglucitol | 0.04761905 | 0.20634921 | 0.2723414 | -1.8765118 |
| Perindopril | 0.02380952 | 0.14606742 | 0.2761578 | -1.8564352 |
| 3-(3-hydroxyphenyl)-3-hydroxypropionic  acid nist | 0.02380952 | 0.14606742 | 0.30669145 | -1.7051401 |
| maleimide | 0.02380952 | 0.14606742 | 0.31136989 | -1.6832987 |
| tocopherol alpha- | 0.02380952 | 0.14606742 | 0.32197021 | -1.6350009 |
| 4-Guanidinobutyric acid | 0.04761905 | 0.20634921 | 0.33615085 | -1.5728193 |
| 4-aminobenzoicacid | 0.04761905 | 0.20634921 | 0.36281588 | -1.4626905 |
| deoxypentitol | 0.04761905 | 0.20634921 | 0.36930609 | -1.4371111 |
| valine | 0.02380952 | 0.14606742 | 0.37892218 | -1.4000265 |
| leucine | 0.04761905 | 0.20634921 | 0.4225447 | -1.2428241 |
| phytanic acid | 0.02380952 | 0.14606742 | 0.42673797 | -1.2285776 |
| 2-aminobutyric acid | 0.04761905 | 0.20634921 | 0.43064958 | -1.2154137 |
| Dehydrocholic acid | 0.02380952 | 0.14606742 | 0.45058824 | -1.1501184 |
| 3-Methylglutarylcarnitine | 0.02380952 | 0.14606742 | 0.45710928 | -1.129389 |
| epsilon-caprolactam | 0.02380952 | 0.14606742 | 0.47233864 | -1.0821065 |
| Trileptal | 0.02380952 | 0.14606742 | 0.4852071 | -1.0433274 |
| parabanic acid NIST | 0.02380952 | 0.14606742 | 0.52026567 | -0.9426796 |
| histidine | 0.02380952 | 0.14606742 | 0.52748586 | -0.9227957 |
| tryptophan | 0.04761905 | 0.20634921 | 0.56261799 | -0.8297724 |
| 4-Hydroxyvalsartan | 0.02380952 | 0.14606742 | 0.56533575 | -0.8228202 |
| 2-hydroxyvaleric acid | 0.02380952 | 0.14606742 | 0.57140444 | -0.8074158 |
| daidzein | 0.02753189 | 0.16163886 | 0.5754717 | -0.7971831 |
| Homoarginine | 0.02380952 | 0.14606742 | 0.59898824 | -0.7394004 |
| Losartancarboxaldehyde | 0.02380952 | 0.14606742 | 0.60906516 | -0.7153315 |
| N-Butylscopolaminium | 0.02380952 | 0.14606742 | 0.636 | -0.6529013 |
| salicylic acid | 0.02380952 | 0.14606742 | 0.67805755 | -0.5605204 |
| thymidine | 0.04761905 | 0.20634921 | 0.68352192 | -0.5489405 |
| phosphate | 0.02380952 | 0.14606742 | 0.68612976 | -0.5434466 |
| 2-methylglutaric acid | 0.04761905 | 0.20634921 | 0.71374829 | -0.4865127 |
| ectoine | 0.04761905 | 0.20634921 | 0.72762148 | -0.45874 |
| Carbamazepine | 0.02380952 | 0.14606742 | 0.77308294 | -0.3713049 |
| 2-Despiperidyl-2-(5-carboxypentylamine)repaglinide | 0.02380952 | 0.14606742 | 0.87037037 | -0.2002987 |
| Zolpidem phenyl-4-carboxylic acid | 0.02380952 | 0.14606742 | 0.90088106 | -0.1505915 |
| Eutylone | 0.02380952 | 0.14606742 | 1.121875 | 0.16591194 |
| Nadolol | 0.02380952 | 0.14606742 | 1.165 | 0.22032996 |
| cholesterol | 0.04761905 | 0.20634921 | 1.27168903 | 0.34674593 |
| N-Methyltyramine | 0.02380952 | 0.14606742 | 1.28639706 | 0.36333601 |
| Phenylethanolamine | 0.02380952 | 0.14606742 | 1.32516892 | 0.40617627 |
| 2S-Amino-4E-octadecene-1,3S-diol | 0.02380952 | 0.14606742 | 1.44078947 | 0.52685955 |
| xylose | 0.04761905 | 0.20634921 | 1.47386578 | 0.55960514 |
| glycolic acid | 0.02380952 | 0.14606742 | 1.47634788 | 0.56203271 |
| Neolinustatin | 0.02753189 | 0.16163886 | 1.54761905 | 0.63005039 |
| trans-4-hydroxy-L-proline | 0.02380952 | 0.14606742 | 1.59221822 | 0.67103807 |
| isothreonic acid | 0.04761905 | 0.20634921 | 1.63953488 | 0.7132866 |
| pantothenic acid | 0.02380952 | 0.14606742 | 1.64010989 | 0.71379248 |
| uracil | 0.02380952 | 0.14606742 | 1.732 | 0.79243893 |
| 7-methylguanine NIST | 0.02753189 | 0.16163886 | 1.77314815 | 0.82631308 |
| 2-hydroxyadipic acid | 0.04761905 | 0.20634921 | 1.84408602 | 0.88290596 |
| Melibiose | 0.02380952 | 0.14606742 | 1.88666667 | 0.91583955 |
| Isomaltose | 0.04761905 | 0.20634921 | 1.89568345 | 0.92271808 |
| 2-Acetylpyrazine | 0.02380952 | 0.14606742 | 1.93865031 | 0.95505259 |
| lactic acid | 0.02380952 | 0.14606742 | 2.04391681 | 1.03133648 |
| Thionin | 0.04761905 | 0.20634921 | 2.13430851 | 1.09376873 |
| ribitol | 0.02380952 | 0.14606742 | 2.13565163 | 1.09467633 |
| Ile-Trp | 0.02380952 | 0.14606742 | 2.15934066 | 1.11059086 |
| hexadecylglycerol NIST | 0.02380952 | 0.14606742 | 2.19503546 | 1.13424425 |
| pseudo uridine | 0.04761905 | 0.20634921 | 2.20163308 | 1.13857405 |
| cystine | 0.02380952 | 0.14606742 | 2.36244248 | 1.2402792 |
| n-acetyl-d-hexosamine | 0.02380952 | 0.14606742 | 2.36628849 | 1.24262598 |
| 4-hydroxyphenylacetic acid | 0.02380952 | 0.14606742 | 2.40561225 | 1.26640412 |
| Galacto-N-biose | 0.04761905 | 0.20634921 | 2.53571429 | 1.3423922 |
| 3-phenyllactic acid | 0.02380952 | 0.14606742 | 2.5550887 | 1.35337338 |
| ribose | 0.02380952 | 0.14606742 | 2.55520118 | 1.35343688 |
| 3,4-dihydroxybenzoic acid | 0.02380952 | 0.14606742 | 2.5613155 | 1.35688497 |
| 1,5-Pentanediamine | 0.04761905 | 0.20634921 | 2.625 | 1.39231742 |
| arabinose | 0.02380952 | 0.14606742 | 2.67821101 | 1.42126963 |
| p-hydroxylphenyllactic acid | 0.02380952 | 0.14606742 | 2.68767429 | 1.42635831 |
| lyxitol | 0.02380952 | 0.14606742 | 2.69424674 | 1.42988198 |
| fucose | 0.04761905 | 0.20634921 | 2.72089947 | 1.44408365 |
| D-Turanose | 0.02380952 | 0.14606742 | 2.73768473 | 1.45295632 |
| threitol | 0.02380952 | 0.14606742 | 2.75243349 | 1.4607077 |
| Sucrose | 0.04761905 | 0.20634921 | 2.91752577 | 1.5447454 |
| hypoxanthine | 0.02380952 | 0.14606742 | 2.95925495 | 1.56523399 |
| Lys-Val | 0.04761905 | 0.20634921 | 2.99247312 | 1.58133829 |
| Betaine | 0.02380952 | 0.14606742 | 3.04135288 | 1.60471322 |
| 5'-deoxy-5'-methylthioadenosine | 0.02380952 | 0.14606742 | 3.88114754 | 1.95648328 |
| mannitol | 0.04761905 | 0.20634921 | 3.93913694 | 1.97787957 |
| methionine sulfoxide | 0.02380952 | 0.14606742 | 3.94460274 | 1.97988001 |
| Ala-Ile | 0.02380952 | 0.14606742 | 3.97860035 | 1.99226099 |
| putrescine | 0.04761905 | 0.20634921 | 4.0515528 | 2.01847494 |
| dihydrocholesterol | 0.02380952 | 0.14606742 | 4.13366337 | 2.04742091 |
| cellobiose | 0.02380952 | 0.14606742 | 4.37755102 | 2.13012399 |
| glutamine | 0.04761905 | 0.20634921 | 4.48888759 | 2.16635797 |
| Val-His | 0.04761905 | 0.20634921 | 5.02877698 | 2.33020757 |
| 2'-O-Methylcytidine | 0.04761905 | 0.20634921 | 5.03385417 | 2.33166342 |
| Trihydroxycholestanoic acid | 0.02380952 | 0.14606742 | 5.1910299 | 2.3760208 |
| Riboflavin | 0.04761905 | 0.20634921 | 5.30476191 | 2.407288 |
| Glu-Val-Lys | 0.02380952 | 0.14606742 | 5.47307692 | 2.45235213 |
| UDP-glucuronic acid | 0.02380952 | 0.14606742 | 5.5 | 2.45943162 |
| 5-hydroxymethyl-2-furoic acid NIST | 0.02380952 | 0.14606742 | 6.06557377 | 2.60064412 |
| N8-Acetylspermidine | 0.02380952 | 0.14606742 | 6.14819495 | 2.62016291 |
| hexuronic acid | 0.02380952 | 0.14606742 | 6.59621657 | 2.72163877 |
| isoribose | 0.02380952 | 0.14606742 | 6.94085028 | 2.79511241 |
| Glycoursodeoxycholic acid | 0.02380952 | 0.14606742 | 7.43292722 | 2.89393048 |
| Glu-Ile-Lys | 0.04761905 | 0.20634921 | 8.07462687 | 3.01339559 |
| Irinotecan | 0.02380952 | 0.14606742 | 8.53097345 | 3.09271037 |
| 2'-O-Methylinosine | 0.02380952 | 0.14606742 | 8.85051546 | 3.14576148 |
| sorbitol | 0.02380952 | 0.14606742 | 9.8753221 | 3.30382781 |
| N,N,N-trimethyl-5-({[(3s,5s,7s)-  tricyclo[3.3.1.1~3,7~]decan-1-yl]methyl}amino)pentan-1-aminium | 0.02380952 | 0.14606742 | 10 | 3.3219281 |
| tartaric acid | 0.02380952 | 0.14606742 | 12.0903615 | 3.59578547 |
| galacturonic acid | 0.02380952 | 0.14606742 | 15.825304 | 3.98416131 |
| 2-monopalmitin | 0.02380952 | 0.14606742 | 23.3938172 | 4.54805538 |
| phenylethylamine | 0.02380952 | 0.14606742 | 39.2008772 | 5.29281403 |
| Tauroursodeoxycholic acid | 0.02380952 | 0.14606742 | 61.5587211 | 5.94389135 |
| Taurocholic acid | 0.02380952 | 0.14606742 | 92.8796582 | 6.53729076 |
| Betonicine | 0.04761905 | 0.20634921 | 117.685083 | 6.87878765 |
| Omeprazole sulfone N-oxide | 0.04761905 | 0.20634921 | 197.026316 | 7.62224453 |
| Omeprazole sulfone | 0.04761905 | 0.20634921 | 561.776316 | 9.13385199 |

| Table S10. Significantly different metabolites in alcholic hepatitis patients with cirrhosis compared with alcoholic hepatitis without cirrhosis | | | | |
| --- | --- | --- | --- | --- |
| compound_names | p_value | p_value_adj | FoldChange | log2FoldChange |
| Omeprazole_sulfone | 0102519 | 0.17792254 | 0804044 | -6.9585101 |
| Omeprazole_sulfone N_oxide | 0.01320656 | 0.25326733 | 0.07899461 | -3.6621019 |
| Gly_Val | 0214855 | 0.17792254 | 0.14345288 | -2.8013512 |
| 1_Stearoyl_2_docosahexaenoyl  _sn_glycero_3_phosphocholine | 0.04205903 | 0.45482438 | 0.34692085 | -1.5273215 |
| PC_36_0 | 0.04865185 | 0.50273578 | 0.37907983 | -1.3994264 |
| Glutamic_acid | 0145429 | 0.17792254 | 0.47354722 | -1.0784198 |
| glutamic_acid | 0346872 | 0.17792254 | 0.47585155 | -1.0714165 |
| Norfenfluramine | 0075771 | 0.17792254 | 0.48684211 | -1.0384741 |
| 1_Stearoyl_2_arachidonoyl_sn  _glycero_3_phospho_1_myo_inositol | 0364267 | 0.17792254 | 0.4997982 | -105824 |
| His_Ala | 0.0132081 | 0.25326733 | 0.54330709 | -0.8801602 |
| N_Octadecanoylsphing_4_enine  _1_phosphocholine | 0.021804 | 0.31683931 | 0.55081401 | -0.8603628 |
| N_Oleoylethanolamine | 0.01212152 | 0.25326733 | 0.63533835 | -0.654403 |
| Ser_Ala | 0.021804 | 0.31683931 | 0.65815279 | -0.6035055 |
| Isoleucine | 0284325 | 0.17792254 | 0.66607801 | -0.5862369 |
| 5_Heptenoic_acid | 0929009 | 0.25326733 | 0.66666667 | -0.5849625 |
| Hydrocortisone | 0.03905362 | 0.45399838 | 0.67070009 | -0.5762603 |
| isoleucine | 0850566 | 0.25326733 | 0.67283354 | -0.5716785 |
| N_Methylvaline | 0.01439732 | 0.25326733 | 0.71473214 | -0.4845254 |
| leucine | 0.0236308 | 0.32159112 | 0.77824249 | -0.3617083 |
| N_Acetyl_tryptophan | 0.0245149 | 0.32159112 | 1.22 | 0.28688115 |
| Glutamine | 0.01969323 | 0.30524501 | 1.2217034 | 0.28889408 |
| malic_acid | 0.0236308 | 0.32159112 | 1.22443044 | 0.29211081 |
| Carvedilol | 0.04035792 | 0.45482438 | 1.28 | 0.35614381 |
| Lauric_acid_diethanolamide | 0.04525607 | 0.47827436 | 1.28036176 | 0.35655149 |
| trans_Piceid | 0.02515522 | 0.32159112 | 1.33333333 | 0.4150375 |
| N_Acetylphenylalanine | 0.01469586 | 0.25326733 | 1.35275081 | 0.4358961 |
| Lansoprazole | 0.01561788 | 0.2593684 | 1.35294118 | 0.43609912 |
| L_Citrulline_L_Citrulline_L_Citrulline | 0.01265893 | 0.25326733 | 1.42983436 | 0.51584802 |
| L_Citrulline_6_2_Aminopropylbenzofuran | 0364324 | 0.17792254 | 1.45113887 | 0.53718559 |
| Glu_Gln | 0972937 | 0.25326733 | 1.46145251 | 0.54740295 |
| sucrose | 0.02558897 | 0.32159112 | 1.57228431 | 0.65286212 |
| 3_Aminohexanoic_acid | 0.01186082 | 0.25326733 | 1.60263654 | 0.68044727 |
| Turanose | 0.01851589 | 0.29689274 | 1.63288136 | 0.70741997 |
| tyrosine | 0442569 | 0.18708609 | 1.66882583 | 0.73883339 |
| citric_acid | 0.02768597 | 0.33878881 | 1.74858506 | 0.80618798 |
| Tyrosine | 0850511 | 0.25326733 | 1.75659575 | 0.81278221 |
| Promethazine_N_oxide | 0.01470585 | 0.25326733 | 1.85546875 | 0.8917837 |
| Biliverden | 0.01086109 | 0.25326733 | 1.88604967 | 0.91536767 |
| p_hydroxylphenyllactic_acid | 0588623 | 0.22809158 | 1.95065699 | 0.96396011 |
| hexitol | 0.03905362 | 0.45399838 | 2.07276967 | 1.05155981 |
| Anthranilic_acid | 0692425 | 0.24767523 | 2.30409357 | 1.20419931 |
| Caffeine | 0382629 | 0.17792254 | 3.36042992 | 1.74864582 |
| Pantoprazole | 0.01110493 | 0.25326733 | 28.4262295 | 4.82915085 |
| Theophylline | 0.04204181 | 0.45482438 | 49.928401 | 5.6417888 |
| Alliin | 0208208 | 0.17792254 | 121 | 6.91886324 |

# Table S11. High levels of methionine and urea are associated with increased 30-Day mortality in patients with alcoholic hepatitis

| Dependent: 30 Day Mortality | | Survival, % (95% CI) | HR (univariable), (95% CI, *P*-value) | HR (multivariable), (95% CI, *P*-value*) |
| --- | --- | --- | --- | --- |
| Indole-3-propioic acid | < 99 | 66.7 (37.5-84.6) | - | - |
|  | ≥ 99 | 87.1 (79.1-92.1) | 0.36 (0.13-0.99, ***P*=0.048**) | 0.25 (0.06-1.01, *P*=0.052) |
| Methionine | < 456 | 97.3 (82.3-99.6) | - | - |
|  | ≥ 456 | 79.4 (69.3-86.5) | 8.01 (1.07-60.02, ***P*=0.043**) | 12.09 (1.39-105.06, ***P*=0.024**) |
| Urea | < 115876 | 93.8 (86.8-97.2) | - | - |
|  | ≥ 115876 | 48.0 (27.8-65.6) | 11.92 (4.52-31.48, ***P*=5.61e-07**) | 10.72 (3.14-36.60, ***P*=1.52e-04**) |

*p-value adjusted for MELD score, antibiotics, steroids, pentoxifylline, and geographic origin. Bold font indicates significance (p-value < 0.05). HR, hazard ratio; CI, confidence interval.

| Table S12. Association of gender and microbes and microbial pathways | | | | | | |  |  |
| --- | --- | --- | --- | --- | --- | --- | --- | --- |
| Variable | feature | gender | coef | stderr | N | N.not.0 | pval | qval |
| Microbes | Anaerostipes_caccae | male | -0.0063 | 0.0020 | 78 | 5 | 0.00237 | 0.65750 |
| Microbes | Odoribacter_unclassified | male | -0.0032 | 0.0012 | 78 | 7 | 0.00731 | 0.65750 |
| Microbes | Parasutterella_excrementihominis | male | -0.0076 | 0.0028 | 78 | 8 | 0.00778 | 0.65750 |
| Microbes | Bacteroides_vulgatus | male | -0.0845 | 0.0316 | 78 | 75 | 0.00920 | 0.65750 |
| Microbes | Burkholderiales_bacterium_1_1_47 | male | -0.0115 | 0.0046 | 78 | 9 | 0.01390 | 0.65750 |
| Microbes | Lactobacillus_phage_Lc_Nu | male | -0.0155 | 0.0062 | 78 | 3 | 0.01539 | 0.65750 |
| Microbes | Gardnerella_vaginalis | male | -0.0113 | 0.0048 | 78 | 3 | 0.02135 | 0.65750 |
| Microbes | Dorea_formicigenerans | male | 0.0257 | 0.0113 | 78 | 47 | 0.02545 | 0.65750 |
| Microbes | Prevotella_timonensis | male | -0.0016 | 0.0007 | 78 | 3 | 0.02600 | 0.65750 |
| Microbes | Clostridiaceae_bacterium_JC118 | male | -0.0056 | 0.0025 | 78 | 12 | 0.02633 | 0.65750 |
| Microbes | Veillonella_parvula | male | -0.0357 | 0.0162 | 78 | 45 | 0.03008 | 0.65750 |
| Microbes | Prevotella_salivae | male | -0.0020 | 0.0009 | 78 | 3 | 0.03107 | 0.65750 |
| Microbes | Bacteroides_uniformis | male | -0.0675 | 0.0311 | 78 | 77 | 0.03321 | 0.65750 |
| Microbes | Clostridium_innocuum | male | -0.0045 | 0.0022 | 78 | 19 | 0.04282 | 0.65750 |
| Microbes | Acidaminococcus_unclassified | male | -0.0285 | 0.0139 | 78 | 12 | 0.04385 | 0.65750 |
| Microbial pathways | HSERMETANA.PWY | male | 0.0026 | 0.0009 | 78 | 73 | 0.00412 | 0.95571 |
| Microbial pathways | PWY.7211 | male | 0.0027 | 0.0010 | 78 | 73 | 0.00751 | 0.95571 |
| Microbial pathways | DENOVOPURINE2.PWY | male | 0.0021 | 0.0009 | 78 | 75 | 0.01748 | 0.95571 |
| Microbial pathways | NONOXIPENT.PWY | male | 0.0032 | 0.0013 | 78 | 77 | 0.01840 | 0.95571 |
| Microbial pathways | PWY.7196 | male | 0.0027 | 0.0011 | 78 | 68 | 0.01856 | 0.95571 |
| Microbial pathways | PWY0.162 | male | 0.0024 | 0.0010 | 78 | 77 | 0.02172 | 0.95571 |
| Microbial pathways | PYRIDNUCSAL.PWY | male | 0.0021 | 0.0009 | 78 | 67 | 0.02265 | 0.95571 |
| Microbial pathways | PYRIDNUCSYN.PWY | male | 0.0029 | 0.0012 | 78 | 75 | 0.02285 | 0.95571 |
| Microbial pathways | PWY.5136 | male | 0.0012 | 0.0005 | 78 | 67 | 0.02560 | 0.95571 |
| Microbial pathways | FAO.PWY | male | 0.0012 | 0.0005 | 78 | 67 | 0.02609 | 0.95571 |
| Microbial pathways | PWY.1042 | male | 0.0029 | 0.0013 | 78 | 77 | 0.02611 | 0.95571 |
| Microbial pathways | PWY.7200 | male | -0.0015 | 0.0007 | 78 | 18 | 0.02796 | 0.95571 |
| Microbial pathways | COMPLETE.ARO.PWY | male | 0.0026 | 0.0012 | 78 | 78 | 0.03238 | 0.95571 |
| Microbial pathways | PWY.6737 | male | 0.0040 | 0.0018 | 78 | 78 | 0.03265 | 0.95571 |
| Microbial pathways | ARO.PWY | male | 0.0027 | 0.0012 | 78 | 78 | 0.03580 | 0.95571 |
| Microbial pathways | PRPP.PWY | male | 0.0019 | 0.0009 | 78 | 73 | 0.03676 | 0.95571 |
| Microbial pathways | PWY.6317 | male | 0.0023 | 0.0011 | 78 | 78 | 0.03692 | 0.95571 |
| Microbial pathways | PWY.6545 | male | 0.0020 | 0.0010 | 78 | 74 | 0.04016 | 0.95571 |
| Microbial pathways | PWY.7187 | male | 0.0015 | 0.0008 | 78 | 75 | 0.04808 | 0.95571 |

| Table S13. Association between gender and serum and fecal metabolites | | | | | | | | |
| --- | --- | --- | --- | --- | --- | --- | --- | --- |
| metadata | Serum Metabolites | gender | coef | stderr | N | N.not.0 | pval | qval |
| Serum | Creatine | male | -0.03548 | 0.00920092 | 50 | 50 | 0.000343 | 0.187033 |
| Serum | lyxose | male | -0.00754 | 0.00253927 | 50 | 50 | 0.004668 | 0.743523 |
| Serum | X3.4.dihydroxyphenylacetic.acid | male | 0.000872 | 0.00029926 | 50 | 48 | 0.005403 | 0.743523 |
| Serum | xylonolactone.NIST | male | -0.00875 | 0.00305041 | 50 | 50 | 0.006137 | 0.743523 |
| Serum | Pantoprazole | male | -0.01666 | 0.00597947 | 50 | 49 | 0.007623 | 0.743523 |
| Serum | lyxitol | male | -0.00446 | 0.00164102 | 50 | 50 | 0.009158 | 0.743523 |
| Serum | glycerol | male | -0.02589 | 0.00987746 | 50 | 50 | 0.011712 | 0.743523 |
| Serum | ribitol | male | -0.00365 | 0.00139694 | 50 | 50 | 0.011904 | 0.743523 |
| Serum | X1.monoolein | male | -0.0064 | 0.00248519 | 50 | 50 | 0.01318 | 0.743523 |
| Serum | tartaric.acid | male | -0.00279 | 0.00110286 | 50 | 50 | 0.014846 | 0.743523 |
| Serum | pentitol | male | -0.00438 | 0.00174054 | 50 | 50 | 0.015354 | 0.743523 |
| Serum | xylitol | male | -0.01034 | 0.00415322 | 50 | 50 | 0.016341 | 0.743523 |
| Serum | X6.deoxyglucose | male | -0.00486 | 0.00200395 | 50 | 50 | 0.018999 | 0.797964 |
| Serum | X2.deoxypentitol.NIST | male | -0.00562 | 0.0023536 | 50 | 50 | 0.020891 | 0.798023 |
| Serum | N.Acetyltryptophan | male | -0.00212 | 0.00089942 | 50 | 50 | 0.022717 | 0.798023 |
| Serum | arabitol | male | -0.0047 | 0.00200652 | 50 | 50 | 0.023431 | 0.798023 |
| Serum | isoleucine | male | 0.016948 | 0.0073163 | 50 | 50 | 0.024847 | 0.798023 |
| Serum | N2.N2.Dimethylguanosine | male | -0.00086 | 0.0003847 | 50 | 50 | 0.02927 | 0.867429 |
| Serum | phthalic.acid | male | -0.00983 | 0.0044005 | 50 | 50 | 0.030185 | 0.867429 |
| Serum | gluconic.acid.lactone | male | -0.00449 | 0.00206951 | 50 | 50 | 0.035159 | 0.878973 |
| Serum | Biotin | male | -0.0007 | 0.00032538 | 50 | 50 | 0.036607 | 0.878973 |
| Serum | beta.alanine | male | 0.002461 | 0.00116888 | 50 | 50 | 0.040515 | 0.878973 |
| Serum | leucine | male | 0.022305 | 0.01081029 | 50 | 50 | 0.044517 | 0.878973 |
| Serum | Lamotrigine | male | -0.00828 | 0.00403504 | 50 | 50 | 0.045528 | 0.878973 |
| Serum | valine | male | 0.023069 | 0.01133603 | 50 | 50 | 0.047394 | 0.878973 |
| Serum | X1.Methylguanosine | male | -0.00059 | 0.00028975 | 50 | 50 | 0.048563 | 0.878973 |
| Serum | Levocetirizine | male | -0.00442 | 0.00218303 | 50 | 50 | 0.048616 | 0.878973 |
| Feces | N.Methylproline | male | -0.02859 | 0.00945792 | 50 | 50 | 0.004006 | 0.881412 |
| Feces | glucose | male | -0.02437 | 0.00852203 | 50 | 50 | 0.006256 | 0.881412 |
| Feces | pyrogallol | male | -0.00285 | 0.00100423 | 50 | 50 | 0.00662 | 0.881412 |
| Feces | lactic.acid | male | -0.03074 | 0.01096316 | 50 | 50 | 0.007265 | 0.881412 |
| Feces | X4.Acetamidobutyric.acid | male | -0.00327 | 0.00118402 | 50 | 50 | 0.008072 | 0.881412 |
| Feces | behenic.acid | male | -0.00592 | 0.00223452 | 50 | 50 | 0.010903 | 0.962211 |
| Feces | X1.2.4.benzenetriol | male | -0.00259 | 0.00103476 | 50 | 50 | 0.015613 | 0.962211 |
| Feces | conduritol.beta.epoxide | male | -0.01634 | 0.00652055 | 50 | 50 | 0.015643 | 0.962211 |
| Feces | Metformin | male | -0.04555 | 0.01909342 | 50 | 50 | 0.021043 | 0.962211 |
| Feces | levoglucosan | male | -0.00352 | 0.00148091 | 50 | 50 | 0.021358 | 0.962211 |
| Feces | mucic.acid | male | -0.00287 | 0.00123561 | 50 | 50 | 0.02448 | 0.962211 |
| Feces | Stachydrine | male | -0.0427 | 0.01862153 | 50 | 50 | 0.026273 | 0.962211 |
| Feces | X2.3.dihydro.8.methoxyfuro.2.3.b.quinoline.NIST | male | -0.00432 | 0.00189283 | 50 | 50 | 0.026993 | 0.962211 |
| Feces | urea | male | 0.006399 | 0.00283558 | 50 | 50 | 0.028607 | 0.962211 |
| Feces | X2.monoolein | male | -0.02983 | 0.01358904 | 50 | 50 | 0.033041 | 0.962211 |
| Feces | phytosphingosine | male | -0.01069 | 0.00496924 | 50 | 50 | 0.036547 | 0.962211 |
| Feces | glucoheptulose | male | -0.00309 | 0.00147872 | 50 | 50 | 0.042094 | 0.962211 |
| Feces | tyrosol | male | -0.00317 | 0.00152186 | 50 | 50 | 0.042689 | 0.962211 |
| Feces | X4.aminobutyric.acid | male | 0.003375 | 0.00163339 | 50 | 50 | 0.044226 | 0.962211 |
| Feces | Metronidazole | male | -0.0022 | 0.00106616 | 50 | 50 | 0.044772 | 0.962211 |
| Feces | lysine | male | 0.017403 | 0.00845407 | 50 | 50 | 0.044989 | 0.962211 |
| Feces | cellobiose | male | -0.04163 | 0.02029726 | 50 | 50 | 0.045734 | 0.962211 |
| Feces | X3..3.hydroxyphenyl..3.hydroxypropionic.acid.nist | male | -0.00378 | 0.00185511 | 50 | 50 | 0.047307 | 0.962211 |
| Feces | Betonicine | male | -0.02303 | 0.01137436 | 50 | 50 | 0.048446 | 0.962211 |
| Feces | Synephrine | male | -0.00503 | 0.00249959 | 50 | 50 | 0.049944 | 0.962211 |

# Supplementary Figure Legends

**Supplementary Figure S1. Study design of multi-omics integration of metabolomics and metagenomics.** Untargeted metabolomic analysis was first performed on a discovery cohort for the significant test between alcoholic hepatitis patients, alcohol use disorder patients and the non-alcoholic controls, and further performed on a validation cohort of alcoholic hepatitis for the purpose of correlation analysis and prediction model development.

**Supplementary Figure S2. Principal component analysis of center effect on metabolomic and metagenomic data.** *(A)* No significant effect was observed on the metabolomic data. *(B)* No significant effect was observed on the metagenomic data.

**Supplementary Figure S3. Principal component analysis of antibiotics effect on metabolomic and metagenomic data.** *(A)* No significant effect was observed on the metabolomic data. *(B)* No significant effect was observed on the metagenomic data. No: patients didn’t receive antibiotics treatment; Yes: patients received antibiotics treatment; NA: information not available.

**Supplementary Figure S4. Principal component analysis of steroids effect on metabolomic and metagenomic data.** *(A)* No significant effect was observed on the metabolomic data. *(B)* No significant effect was observed on the metagenomic data. No: patients didn’t receive steroids treatment; Yes: patients received steroids treatment; NA: information not available.

**Supplementary Figure S5. Spearman correlation of key metabolites with clinical outcomes in patients with alcoholic hepatitis.** *: adjusted p-value < 0.05, **: adjusted p-value < 0.01, ***: adjusted p-value < 01. GGT: gamma-glutamyl-transferase; AST: aspartate aminotransferase; ALT: alanine aminotransferase; INR: international normalized ratio; MELD: model for end-stage liver disease.

**Methods**

**Inclusion and exclusion criteria of patients with alcoholic hepatitis**

Inclusion criteria for alcoholic hepatitis were: 1. active alcohol use (> 50 g/day for men and > 40 g/day for women) in the last 3 months; 2. aspartate aminotransferase (AST) > alanine aminotransferase (ALT) and total bilirubin > 3 mg/dl in the past 3 months; 3. liver biopsy and/or clinical picture consistent with alcoholic hepatitis. Exclusion criteria were: 1. autoimmune liver disease (ANA > 1/320); 2. chronic viral hepatitis; 3. hepatocellular carcinoma; 4. complete portal vein thrombosis; 5. extrahepatic terminal disease; 6. Pregnancy; 7. lack of signed informed consent.

**Metabolomics Data Acquisition**

The sample extraction was performed as reported previously (1). Primary metabolites were analyzed by gas chromatography-time of flight mass spectrometry (GC-TOF MS) as published previously (1). A 0.5 µL sample was injected on an Agilent 6890 GC (Agilent Technologies, Santa Clara, CA, USA) with 25 s splitless time. A Restek Rtx-5Sil MS column (30 m x 0.25 mm x 0.25 µm) with 10 m guard column (10 m x 0.25 mm x 0.25 µm) was used. Detailed parameters about GC and MS settings were reported in our previous study (2). Biogenic amines, methylated metabolites, dipeptides, and other polar metabolites were analyzed using hydrophilic interaction liquid chromatography (HILIC) with quadrupole orbital ion trap high field mass spectrometry (Q-Exactive HF MS). A 5 µL re-suspended sample was injected onto an Acquity UPLC BEH Amide column (150 mm x 2.1 mm x 1.7 µm) coupled with an Acquity VanGuard BEH Amide pre-column (5 mm x 2.1 mm x 1.7 µm, Waters, Milford, MA). A Vanquish UHPLC system (Thermo Scientific, Waltham, MA, USA) was used. Spectra was collected in ESI positive mode using a ThermoFisher Q-Exactive HF with a HESI-II ion source (Thermo Scientific, Waltham, MA, USA) with a data-dependent MS/MS spectra acquisition method. Detailed parameters about LC and MS settings were reported in our previous study (2). As quality control samples, human plasma (BioIVT, Westbury, NY, USA) and method blank samples were injected at the beginning of the run and every ten samples throughout the run.

**Metabolomics Data Processing**

GC-MS raw data were processed by ChromaTOF version 4.50 and Binbase version 5.0.3 (3, 4). Detailed parameters used in ChromaTOF and Binbase have been reported in our previous study (2). LC-MS raw data files were converted to ABF files using ABF converter (https://www.reifycs.com/AbfConverter/) and then processed by MS-DIAL version 2.94 (5) and MS-FLO (6). Detailed parameters used in MS-DIAL and MS-FLO have been reported in our previous study (2). For compound identification in HILIC dataset, retention time - m/z libraries and MS/MS spectra databases were used, which were uploaded to MassBank of North America (7).

**Shotgun Metagenomics Analysis**

DNA from human stool samples was extracted using FastDNA Spin Kit for Soil (MP-Biomedicals). Whole-genome shotgun metagenomic sequencing was performed on Illumina HiSeq 4000 generating 150bp paired-end reads. Quality control of shotgun metagenomic reads was performed using KneadData version 0.7.2. Metagenomic Phylogenetic Analysis 2 (MetaPhlAn2) version 2.7.7 was used for the profiling of the composition of the microbial communities (8). The HMP Unified Metabolic Analysis Network 2 (HUMAnN2) version 0.11.1 was used for the profiling of microbial pathways (9). MetaCyc database was used for microbial pathway analysis (10). Each of the HUMAnN2 abundance output was normalized into relative abundance (the counts for each sample sum to 100). Linear discriminant analysis effect size (LEfSe) was used for the biomarker discovery, which considers statistical significance with biological consistency as well as effect size estimation (11). Sequence data were deposited in the European Nucleotide Archive under accession numbers ERP106878.

**References**

1. Fiehn O. Metabolomics by Gas Chromatography-Mass Spectrometry: Combined Targeted and Untargeted Profiling. Current protocols in molecular biology 2016;114:30.34.31-30.34.32.

2. Gao B, Lue H-W, Podolak J, Fan S, Zhang Y, Serawat A, Alumkal JJ, et al. Multi-Omics Analyses Detail Metabolic Reprogramming in Lipids, Carnitines, and Use of Glycolytic Intermediates between Prostate Small Cell Neuroendocrine Carcinoma and Prostate Adenocarcinoma. Metabolites 2019;9:82.

3. Skogerson K, Wohlgemuth G, Barupal DK, Fiehn O. The volatile compound BinBase mass spectral database. BMC bioinformatics 2011;12:321-321.

4. Kind T, Wohlgemuth G, Lee DY, Lu Y, Palazoglu M, Shahbaz S, Fiehn O. FiehnLib: mass spectral and retention index libraries for metabolomics based on quadrupole and time-of-flight gas chromatography/mass spectrometry. Analytical chemistry 2009;81:10038-10048.

5. Tsugawa H, Cajka T, Kind T, Ma Y, Higgins B, Ikeda K, Kanazawa M, et al. MS-DIAL: data-independent MS/MS deconvolution for comprehensive metabolome analysis. Nature methods 2015;12:523-526.

6. DeFelice BC, Mehta SS, Samra S, Čajka T, Wancewicz B, Fahrmann JF, Fiehn O. Mass Spectral Feature List Optimizer (MS-FLO): A Tool To Minimize False Positive Peak Reports in Untargeted Liquid Chromatography–Mass Spectroscopy (LC-MS) Data Processing. Analytical Chemistry 2017;89:3250-3255.

7. Barupal DK, Zhang Y, Shen T, Fan S, Roberts BS, Fitzgerald P, Wancewicz B, et al. A Comprehensive Plasma Metabolomics Dataset for a Cohort of Mouse Knockouts within the International Mouse Phenotyping Consortium. Metabolites 2019;9:101.

8. Truong DT, Franzosa EA, Tickle TL, Scholz M, Weingart G, Pasolli E, Tett A, et al. MetaPhlAn2 for enhanced metagenomic taxonomic profiling. Nature Methods 2015;12:902.

9. Franzosa EA, McIver LJ, Rahnavard G, Thompson LR, Schirmer M, Weingart G, Lipson KS, et al. Species-level functional profiling of metagenomes and metatranscriptomes. Nature methods 2018;15:962-968.

10. Caspi R, Billington R, Fulcher CA, Keseler IM, Kothari A, Krummenacker M, Latendresse M, et al. The MetaCyc database of metabolic pathways and enzymes. Nucleic acids research 2018;46:D633-D639.

11. Segata N, Izard J, Waldron L, Gevers D, Miropolsky L, Garrett WS, Huttenhower C. Metagenomic biomarker discovery and explanation. Genome biology 2011;12:R60-R60.
